# Sequence specificity between interacting and non-interacting homologs identifies interface residues – a homodimer and monomer use case

**DOI:** 10.1186/s12859-015-0758-y

**Published:** 2015-10-08

**Authors:** Qingzhen Hou, Bas E. Dutilh, Martijn A. Huynen, Jaap Heringa, K. Anton Feenstra

**Affiliations:** 1 Center for Integrative Bioinformatics VU (IBIVU), Vrije University Amsterdam, De Boelelaan 1081A, 1081 HV Amsterdam, The Netherlands; 2 Theoretical Biology and Bioinformatics, Utrecht University, Padualaan 8, 3584 CH Utrecht, The Netherlands; 3 Centre for Molecular and Biomolecular Informatics, Radboud Institute for Molecular Life Sciences, Radboud University Medical Centre, Geert Grooteplein 28, 6525 GA Nijmegen, The Netherlands; 4 Department of Marine Biology, Institute of Biology, Federal University of Rio de Janeiro, Rio de Janeiro, Brazil

**Keywords:** Protein-protein Interaction, Sequence specificity, Non-interacting homologs, Sequence-based interface prediction, Sequence harmony

## Abstract

**Background:**

Protein families participating in protein-protein interactions may contain sub-families that have different binding characteristics, ranging from right binding to showing no interaction at all. Composition differences at the sequence level in these sub-families are often decisive to their differential functional interaction. Methods to predict interface sites from protein sequences typically exploit conservation as a signal. Here, instead, we provide proof of concept that the sequence specificity between interacting versus non-interacting groups can be exploited to recognise interaction sites.

**Results:**

We collected homodimeric and monomeric proteins and formed homologous groups, each having an interacting (homodimer) subgroup and a non-interacting (monomer) subgroup. We then compiled multiple sequence alignments of the proteins in the homologous groups and identified compositional differences between the homodimeric and monomeric subgroups for each of the alignment positions. Our results show that this specificity signal distinguishes interface and other surface residues with 40.9 % recall and up to 25.1 % precision.

**Conclusions:**

To our best knowledge, this is the first large scale study that exploits sequence specificity between interacting and non-interacting homologs to predict interaction sites from sequence information only. The performance obtained indicates that this signal contains valuable information to identify protein-protein interaction sites.

**Electronic supplementary material:**

The online version of this article (doi:10.1186/s12859-015-0758-y) contains supplementary material, which is available to authorized users.

## Background

Protein-protein interactions (PPIs) play a central role in virtually all cellular processes. Proteins interact with other proteins to accomplish specific biological functions, such as DNA replication or RNA transcription, gene translation, gene regulation and protein transport, as well as signal transduction. Identification of interaction sites between two binding proteins is essential to understand complex formation and investigate their function (e.g., [13, 20, 53]). In particular, information about specific amino acid residues that play essential roles in protein interactions usually has a wide range of applications such as design of the targets of drugs and antimicrobials (e.g., [40]).

Despite continual improvement, certainly over the last decade, experimental techniques for large scale determination of PPIs are not yet able to provide comprehensive coverage over all PPIs in the detail needed to allow better understanding of the evolutionary and physical forces that govern them (e.g., [13, 23, 24]).

During the past decades, several types of computational methods have been developed for protein interaction prediction. Docking and modeling approaches that rely mainly on surface complementarity and electrostatics to predict structural complexes. These approaches fit together two known structures through their interacting surfaces, or predict protein–protein interaction sites from known monomer structures [13, 23, 41, 47]. However, these methods require structure information of proteins, which remains relatively scarce and expensive. Therefore with the increasing amount of sequence data from sequencing initiatives, a method that only uses sequence information without known structures to predict protein-protein interaction sites is becoming increasingly attractive.

Several such computational methods aim to predict the possibility of interaction between two proteins [15, 35, 52]. Perhaps the most well-known technique for predicting PPIs from sequence data is the ‘mirror tree’ method (e.g., [38]). This method infers interactions from the correlation of evolutionary patterns, as seen in phylogenetic trees representing each of the interaction partners. However, this correlation may instead arise from functional relatedness as well as a number of other general evolutionary mechanisms [8, 29].

Predicting intra-protein and inter-protein residue-residue contacts from sequence covariation has recently revived [19, 22, 32–34, 51, 55]. This is directly due to the availability of large amounts of sequence data and the recent development of so-called direct-coupling methods (e.g., [55]. The idea has been studied in the eighties (e.g., [1]) and nineties (e.g., [16, 27]). The main limitation of these methods is that, typically, five times more sequences than the alignment length (the ‘5 L’ rule) are required [21, 22, 32, 33, 51]. For most proteins, this is not available. In addition, the application to inter-protein residue contacts is hampered by the need to construct large *correlated* alignments. Here, for each sequence an ortholog in the other alignment must be included, so that positional variations of the alignment of one interaction partner may be correlated with those of the other protein.

For identifying protein-protein interaction (PPI) sites, often conservation measures on sequence features are used [45]. For example, ISIS by Ofran and Rost combine PSI-blast profiles and predicted solvent accessibility and secondary structure to predict interface sites [35, 36]. SPPIDER [43] uses in addition several structure-derived features in an elaborate Machine learning approach. In addition, sequence and network features [12, 48], as well as conservation in combination with specificity [31] are also used to predict interaction sites. Several findings indicate that the interface rim tends to be more conserved than the interface core (e.g., [5, 18, 44]), while localized conservation of single residues can indicate interaction hot spots [9, 35, 50]. At the level of PPI networks, mixed results are being reported. Some conserved PPI network motifs appear related to conserved sequence motifs [12, 48]. Overall conservation patterns, however, are found to be weak and mostly not significant (e.g., [28, 42]).

Although progress has been made in predicting binding sites from sequence information, the problem remains far from solved and several limitations persist. First, extracting evolutionary information from sequence data critically depends on sequence alignments containing large numbers of sequences. Second, most methods rely on a combination of structural and sequence features (e.g., [52, 54]). While combined methods can achieve high prediction performance, the performance of sequence-only methods remains modest [35, 37, 42].

Specificity of interaction, i.e. differences between groups of homologs that display different interactions has previously been reported. Pirovano et al. [39] identified interface residues by comparing homologs with different binding partners. Manning et al. [31] predicted positions which define sequence subfamily specificity, where some of these positions were binding sites. Based on a dataset of yeast interaction data and fungal ortholog groups, it has been suggested that, in addition, specificity between non-interacting and heteromeric interacting protein pairs might be used to detect the interaction sites [14]. Interestingly, here only up to one hundred sequences were needed to detect the specificity signal between binding and non-binding groups, far fewer than the ‘5 L’ needed for covariation-based methods. However, the performance of their predictions is only just above random, indicating a need for a cleaner dataset for obtaining proof of principle.

In this paper, we investigate whether specificity between interacting and non-interacting subgroups can be used to predict interaction sites. To address this question, we chose homodimers as a use case to construct interacting subgroups and monomers to constitute non-interacting subgroups. In this way, we can confirm that all sequences in the interacting subgroup physically interact, and that we have a sub-group of monomers known not to (self) interact. Furthermore, the specificity signal is from compositional differences of one chain rather than multiple chains as would be the case when comparing heteromeric interacting groups with non-interacting groups. All homodimers and monomers were obtained from PISA which is a resource for exploring marcromolecular interfaces [26]. We compiled a new database derived entirely from crystallized proteins in the PDB [4], and compared homodimers with homologous non-interacting monomers in a multiple sequence alignment. Starting with 9152 homodimers and 13,355 monomers, we constructed 1,592 pair groups for which we predicted putative interface residues. We found that the compositional differences between interacting and non-interacting subgroups pinpoint interface positions. We also found that various filters on the input sequences yielded a stronger specificity signal and a better prediction performance. Finally, we relate our method with a sequence-only method, SPPIDER [43].

## Methods

### Datasets

**Fig. 1:**
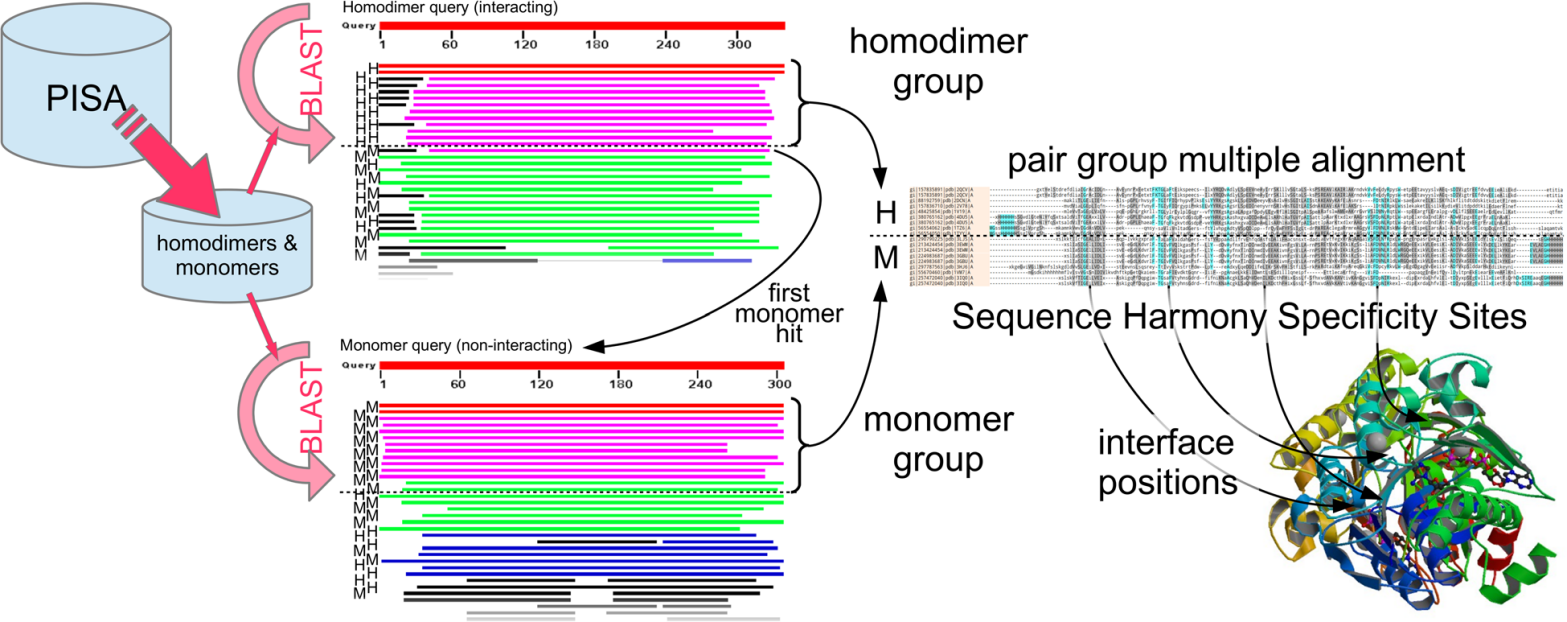
Scheme of building interacting and non-interacting subgroups. This figure shows how we construct and align the subgroups for detecting interface positions from specificity, and validation based on structure. Details are in main text

To investigate conservation difference between interacting and non-interacting homologs, a comprehensive, and consistent dataset describing interactions, non-interactions, homology and interfaces was needed (Fig. 1). For this reason, we selected homodimers as interacting and monomers as non-interacting proteins from the PISA database [26]. We created two test sets to evaluate prediction performance. Test-set 1 includes a comprehensive list of 9152 (interacting) homodimer sequences and 13,355 (non-interacting) monomer sequences (before year 2013) and Test-set 2 contains 1416 homodimers and 2453 monomers (2013 and later). All of the sequences also satisfy the following criteria:

1. The length of the sequences is at least 50 amino acids.
2. None of two sequences in either groups is identical.
3. In addition to the PISA annotation, all homodimeric and monomeric proteins are also defined as homodimers and monomers respectively in PDB.

A list of the selected homodimers and monomers can be found in the Additional file 1: Table S8 and 9).

For Test-set 1, we constructed 10 datasets based on the sequence identity (% ID) filtering ranging from 40 % to 100 % ID (*e.g.*, 40 % means no two sequences in the dataset have more than 40 % identity): <40 %, <50 %, <60 %, <70 %, <80 %, <90 %, <95 %, <98 %, <99 %, <100 % (i.e., non-identical sequences). The filtering was done by using CD-hit [30]. Only the longest sequence was retained of a set of sequences above the sequence identity threshold. For Test-set 2, we only use the <100 % dataset.

Structural information of the homodimers from the description of interface region by PISA was employed to identify interface, surface and buried residues. We used Accessible Surface Area (ASA) before association and Buried Surface Area (BSA) during the association as defined by PISA. ASA indicates the solvent-accessible surface area of the corresponding residues and BSA means the solvent-accessible surface area of the residue that is buried upon interface formation. We use these two parameters to divide the residue positions into the three categories:

1. Interface sites (interacting residues): ASA > 0 and BSA > 0
2. Surface residues (Solvent-accessible residues): ASA > 0 and BSA = 0
3. Buried residues (Inaccessible residues): ASA = 0 and BSA = 0

### Interacting and non-interacting homologs

To investigate conservation differences of interaction positions between interacting and non-interacting homologs, knowing the homologous relationship between a set of interacting homologs and a set of non-interacting homologs is essential for grouping the sequences. Using the sequence sets defined above, we created paired homodimer-monomer alignments. The paired alignments derived from the <100 (non-identical) sequence set, we refer to as the 'complete' set. Figure 1 summarizes the scheme to get homologous groups of interacting and non-interacting subgroups. We did this for each of the 10 sequence datasets of Test-set 1.

First, BLASTP [2] is used to detect homologous relationships in an All-against-All comparison in our custom database of all 9152 (homodimer) and 13,355 (monomer) sequences combined. For each homodimer query sequence, we search for the nearest non-interacting (monomer) homolog first. The set of interacting (homodimer) sequences that are closer (lower BLAST e-values so up in the list) than the nearest monomer sequence constitute the interacting subgroup. A minimum of five homologs is required to form a subgroup. Subsequently, the first monomer hit is also used as query, and, symmetrically, all monomer hits closer than the first homodimer hit constitute the non-interacting subgroup (again with a minimum of five sequences). These two subgroups together then compose an interacting and non-interacting pair group for conservation and specificity analysis. The requirement that each sub-group has at least five sequences ensure the necessary evolutionary information is obtained for analysis. MUSCLE [11], a fast alignment method, was used with default parameters to build the approximately 20,000 alignments for all the sequences in the pair group. Default parameters are used when running BLASTP and MUSCLE.

For Test-set 2, all 1416 homodimers and 2453 monomers are used to obtain homologous groups of interacting and non-interacting subgroups following the method described above. The difference is we use all the sequences (Test-set 1+ Test-set 2) as a blast database to get enough sequence information.

Two filtering conditions were applied on our custom sequence database to test whether these might influence the specificity signal of the interface positions:

1. interacting (homodimer) protein length cut-off ranging from 50 to 600 amino acids as minimum sequence length.
2. High scoring Segment Pair (HSP) length from BLAST between the homodimer and its first homologous monomer hit ranging from 25 to 200 amino acids as minimum length cut-offs. The HSP length between a homodimer and its first monomer hit was used as reported by BLAST when finding the interacting and non-interacting homologs.

### Scoring for conservation

To describe the degree of conservation, sequence entropy [46, 49] is used to indicate the differences between varied positions. The formula of entropy calculation for a column *i* is expressed as:

$$ {S}_i\kern1em =\kern1em -{\displaystyle \sum_x{p}_{i,x}} \log {p}_{i,x} $$

where *p*_*i,x*_ means the fraction of amino acid *x* at the *i*-th position of the sequence; the sum is over all 20 amino acids. A low sequence entropy *S*_*i*_ represents higher evolutionary conservation. We calculated average entropies for each pair group for comparison among varied positions.

### Detecting Specificity signal

Sequence Harmony [6, 39], an entropy based method, is applied to detect the compositional differences between subgroups. The program is accessible as a web-server from: http://www.ibi.vu.nl/programs/seqharmwww/.

With a multiple alignment with sequence groups labelled, Sequence Harmony (SH) measures the overlap in amino acid frequencies between labeled groups at a certain alignment position *i* as follows:

$$ S{H}_i\kern1em =\kern1em -1/2{\displaystyle \sum_x{p}_{i,x}^H} \log \frac{p_{i,x}^H}{p_{i,x}^M+{p}_{i,x}^H}-\kern1em 1/2{\displaystyle \sum_x{p}_{i,x}^M} \log \frac{p_{i,x}^M}{p_{i,x}^H+{p}_{i,x}^M} $$

where *p* ^*H*^_*i,x*_ indicates the observed frequency in homodimer group H for amino acid type *x* at position *i* in the sequence, and analogously for monomer group M, and the sum is over all 20 amino acids . Therefore, an SH score of 0 indicates an amino acid position that has no co-occurring residues in the two groups, indicating complete specificity between the two sequence groups, whereas an SH score of 1 indicates a complete compositional overlap between the two groups at this amino acid position. SH scores were calculated between the interacting (H) and non-interacting (M) pair group at each position in the alignment. The lower-scoring sites are then predicted to constitute the interface region.

### Detecting Specificity signal

Using the interface sites as defined above, we analyse how predictive the specificity signal is to recognize the interaction sites. We used the area under the curve (AUC) of the receiver operator characteristic (ROC; true positive rate vs. false positive rate) plot to evaluate the performance of the specificity signal. Average AUC is calculated for each of the filtered datasets described above. We also used our SH-score as a two-state prediction method by applying a cut-off of SH ≤ 0.2 (0.2 was recommended in [39]). True positives (TP) are true interface residues predicted correctly, false positives (FP) denote false prediction of interaction sites, true negatives (TN) designate non-interacting sites that the specificity signal recognized, and false negatives (FN) are true binding sites which were not predicted. Precision and recall were also calculated for comparison with other tools. The formulas used are as follows:

- Recall (True Positive Rate, Sensitivity or Coverage) = TP/(TP + FN)
- False Positive Rate (FPR) = FP/(TN + FP)
- Precision (Positive Predictive Value) = TP/(TP + FP)

### Comparison with other method

SPPIDER [43] is a machine learning-based methods that can predict interaction sites using sequence information only, although, support for sequence-only prediction in SPPIDER is experimental. We tested the performance of the SPPIDER web server using the same datasets as we used for our method. Precision and Recall were calculated for each method to compare performance.

## Results

We applied our analysis on the 10 pair-group sets created based on sequence identity (i.e., the complete set, <99 %, <98 %, <95 %, <90 %, <80 %, <70 %, <60 %, <50 % and <40 %) for Test-set 1. The number of pair-group alignments in each of the sets can be found in Additional file 1: Table S1. With lower sequence identity cut-off values, the numbers of homodimer and monomer sequences within each of the groups and the total number of groups are reduced. The complete set (Test-set 1) consists of 1593 pair-group alignments, each containing on average 25 homodimer and 14 monomer sequences per group.

### Conservation differs for buried sites

**Fig. 2:**
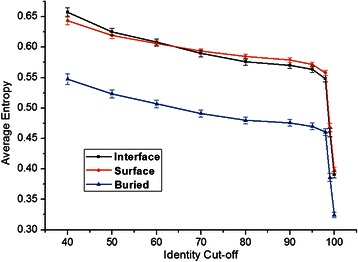
Conservation differs for buried sites. The conservation differences in interacting (homodimer) sub-group between the three types of positions with sequence identity cut-off (Test-set 1). Average entropies were calculated to describe the difference. ‘Interface’, ‘Surface’ and ‘Buried’ stand for Interaction sites, other Surface and Buried inaccessible residues, respectively. Buried sites show distinctly lower entropy (higher conservation), but differences between surface and interface are negligible

From the alignments created, for each position the entropy was calculated to quantify the evolutionary conservation pattern. Figure 2 shows the conservation difference between three types of positions: interface, other surface and buried residues in the homodimer subgroup. For each alignment, the overall average entropies for each of the three types of positions were calculated for the homodimer subgroup.

As described in methods, interface sites stand for the residues for which accessible surface area becomes buried during association; surface residues are solvent-accessible, but not interface; and buried residues are inaccessible. When filtering on maximum sequence identity from <40 % to <100 % (<100 % yielding the complete dataset), average entropies went down because more similar sequences were included in the datasets. It was not surprising that the buried (inaccessible) sites were more conserved than the other two (interface and other surface sites). During evolution, proteins usually conserve their hydrophobic core to keep structural stability. However, the conservation differences between interface and the rest of the protein surface were small over the whole range of % ID cut-offs.

In summary, it is easy to separate the buried sites from surface residues because the conservation pattern differs. However, it is virtually impossible to distinguish the interaction sites from other surface sites using sequence conservation information only, because differences in conservation are generally negligible.

### Specificity differs between interface, surface and buried positions

Next, we calculated the specificity between interacting and non-interacting groups for the three aforementioned types of positions using Sequence Harmony. In our hypothesis, the lower SH scores should be located at the interface positions. We took the complete dataset to show the specificity. Sequence Harmony detected compositional differences between interacting and non-interacting sub-groups for the three different types of positions. The overall average SH value for interface is 0.358, for other surface 0.375 and buried position 0.380 (*p*-value ≤ 0.05 between interface and other surface using two-sided Student's *t*-test). Indeed, the interface positions show the lowest SH scores. In other words, there is signal present in the specificity.

**Fig. 3:**
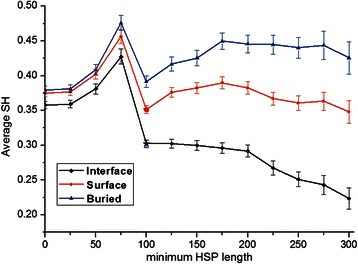
The different trends of SH scores at three kinds of positions. The different trends of SH scores at the three kinds of positions with increasing minimal HSP length (0 to 300 aa) from BLAST (Test-set 1). 0 means all sequences (longer than 0 HSP length. The SH of interacting sites was lowest and separation with the other two increased with increasing HSP length. 'Star' in plot means from 100 aa HSP length, the differences between interface and surface become significant (*p*-value <10^−8^)

We investigated whether there was a subset of our pair-groups which could score much stronger SH signals. Two parameters were used to make different dataset selections. First is the length of the High-Scoring Segment Pair (HSP) between query homodimer and its first monomer hit as given by BLAST. The HSP length cut-off was used to exclude hits which only consisted of small local alignments from the BLAST results. Our pair groups should reflect homologous relationships between two groups of homologs: the homodimer group which conserves interaction, and the monomer group which conserves non-interaction. Since longer HSPs reflect stronger evidence for a homologous relation, we consider longer HSP hits reported by BLAST as more relevant. With increasing HSP-length, the difference between the average SH scores of the different positions grows steadily, as can be seen in Fig. 3. This is caused by the SH scores of the interaction sites decreasing more rapidly than the scores for the two other types of positions. The differences of average SH values between interface and surface become even more significant from 100 aa to 300 aa minimal HSP length (*p*-value ≤ 10^−5^ using two-sided Student's *t*-test). In other words, the specificity signal appears to be able to separate interaction sites out of both the other surface sites and buried residues, and better for the more significant, longer HSP pair groups. The numbers of pair groups retained after filtering on HSP-length and sequence length are reported in Additional file 1: Table S2 and S3.

The second selection parameter was the interacting (homodimer) sequence length. Additional file 1: Figure S1 shows the changes of average SH score with increasing sequence length (of the interacting protein). The figure shows that with increasing sequence length, the differences between the SH scores of the three groups remained rather stable, unlike the diverging trend observed for HSP-length selection (Fig. 3.).

### Interface prediction depends on HSP length and % ID filtering

In light of the observed trends in differences of SH scores, we tested if the specificity signal could be used to predict interaction sites. Predictions were validated against interaction site annotations obtained from the PISA database (see Methods for details). For all pair groups, the Area Under the Curve (AUC) of Receiver Operator Characteristic (ROC) plots was calculated as a measure of performance.

**Fig. 4:**
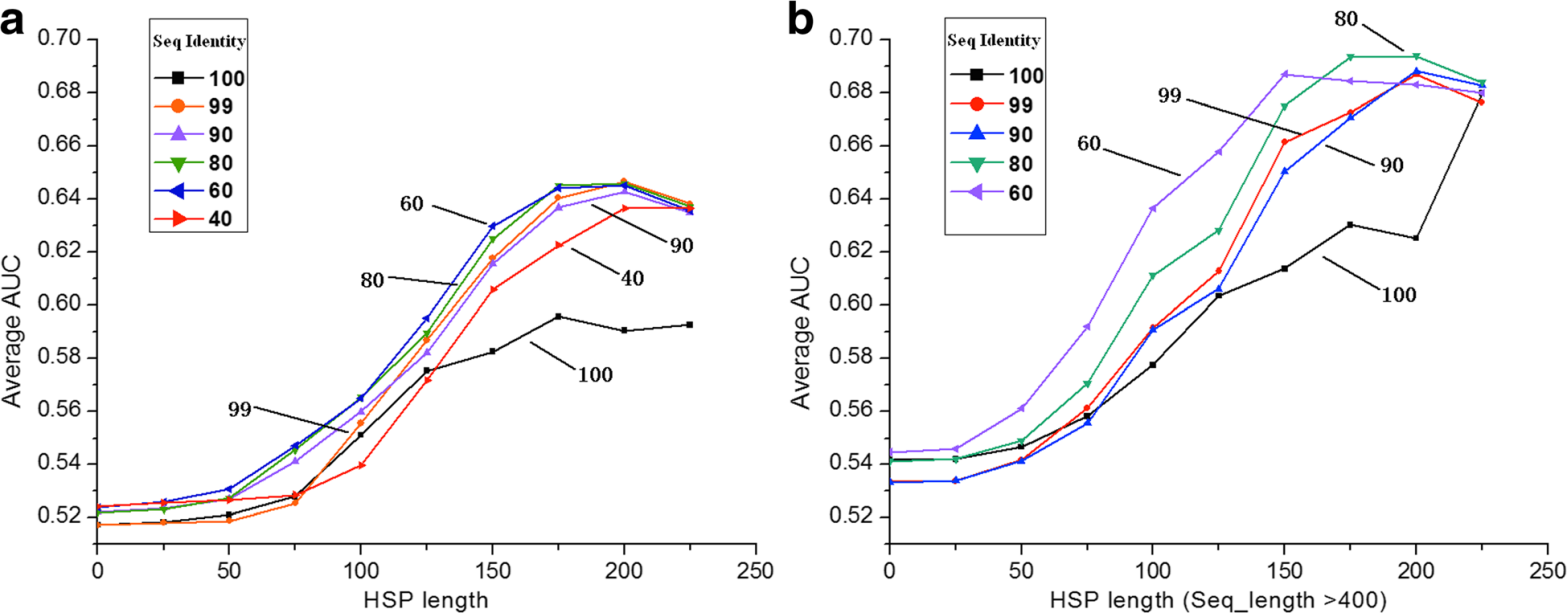
Interface prediction depends on HSP length and % ID filtering. **a** Average AUC with the increasing HSP-length from 0 to 225. Each line shows one dataset with identity cut-off. **b** Same as **a** but with minimal sequence length 400. The AUC with the dataset filtering at 40 % is not shown because a limited number of groups remain. The chart which shows the full set of sequence identity cut-offs can be seen in Additional file 1: Figure S2. The dataset used here is Test-set 1

Figure 4 presents the average AUC versus increasing HSP-length. As can be seen, the performance is rising with increasing HSP length, as expected based on Fig. 3. Moreover, the performance also increases with lower sequence identity cut-offs. For the dataset filtered at 50-99 % ID, the average AUC reaches a maximum of around 0.64-0.65, a feasible range for prediction purposes. Figure 4B shows that the AUC values may be increased further by combining the sequence identity cut-off with a minimum sequence length. Interestingly, the <100 % ID set performs worse than the others at higher HSP length cut-offs. This set contains many single-residue mutants that appear to bias the scoring. The highest average AUC increased to about 0.7 with the dataset selected by sequence identity <70-98 %, sequence length >400 aa and HSP length between interacting and non-interacting group >200 aa. The AUC with the datasets filtered at a 40 % identity cut-off, or at HSP-length >250 aa are not shown because too few pair-groups remain (see also Additional file 1: Table S3).

**Fig. 5:**
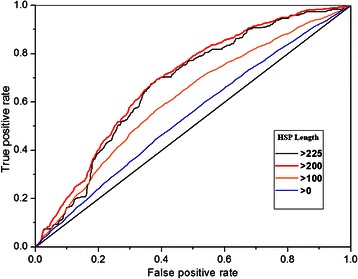
Interface prediction performance. ROC-plots measuring performance of interface prediction at different HSP-length with minimal sequence length 400 (<80 % ID dataset). The diagonal line represents a random prediction. The chart showing all HSP length thresholds used can be seen in Additional file 1: Figure S3. The dataset used here is Test-set 1

Thus sequence length and HSP length were used in combination to select a final dataset. In this part we select the dataset filtered at 80 % ID for performance reasons. Figure 5 shows the ROC plots using different HSP cut-off values and a minimal length of 400 aa. All ROC plots show a better-than-random performance of prediction. Also with minimal sequence length 400 aa, the average AUC was improving when the HSP length was increasing. Here, we could achieve an AUC of 0.7 using an HSP length of 175 aa or 200 aa.

### Interface prediction is a challenging problem

Since we could achieve reasonably good AUC values for our SH method, we compared our approach with SPPIDER [43], using the same datasets. Table 1 shows precision and recall for our method and SPPIDER. For comparison, both the complete and selected datasets are used. Because there is no scriptable version available for SPPIDER, we use SPPIDER only for the smaller Test-set 2 and the selected Test-set 1 (HSP-length > 200 aa and sequence length >400 aa). With our complete Test-set 2, the precision of SPPIDER is slightly better than our method (24.8 % vs 22.8 %). However, our method (SH ≤ 0.2) covered much more interacting sites (recall 41.3 % versus 8.9 % for SPPIDER). With the selected Test-set 1, both the precision and recall (coverage) of our method are the better of the two methods, with recall for our method far exceeding that of SPPIDER. For Test-set 2, an independent dataset, we get very similar prediction performance as for Test-set 1 using both the Complete and Selected datasets.

**Table 1:** Comparison of performance between SPPIDER and our SH

	Size	SPPIDER		SH	
		Prec	Recall	Prec	Recall
Complete set1 : <100 % ID (Test-set 1)	1592	1)		22.3 %	41.7 %
Complete set2 : <100 % ID (Test-set 2)	40	**24.8 %**	8.9 %	22.8 %	**41.3 %**
Selected Test-set 1: 200HSP +400length	52	24.9 %	6.9 %	**25.1 %**	**40.9 %**
Selected Test-set 2 : 200HSP +400length	1	2)		23.1 %	50.5 %

Table 1 Four datasets: The full set at 100 % ID and a selected set by using minimal HSP-length 200 and sequence length 400. Higher Precision and Recall for each dataset in bold. 1) SPPIDER not run 2) Only one protein family and SPPIDER gave no correct prediction

### Predicting the interface that stabilizes the ligand binding regions in a phosphatase and a kinase family

To illustrate the impact of accurate interface prediction, we here show details of two protein families with the highest AUC in ROC performance in our dataset, indicating the specificity signal strongly correlates with the interface region.

**Fig. 6:**
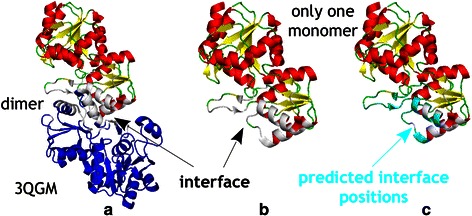
Interface prediction example. **a** Secondary stucture of two chains of PDB 3QGM (chain C and D). The interface is in white, chain C is in blue and different colors in chain D show helices (red), sheet (yellow) and loop (green). **b** The interface (in white) for one monomer. **c** The interface sites (cyan) we predicted correctly

The first example is chronophin, a cofilin-activating phosphatase of the haloacid dehalogenase (HAD) super-families, directly dephosphorylates cofilin with high specificity [17]. C2a-type HAD Hydrolases, usually show interaction between two chains, froming a homooligomer chronophin. This phosphatase subfamily is only active as oligomer, because the interactions are crucial for substrate specificity loop positioning, while other subfamilies may not all require oligomerization [25]. We constructed pair groups using 3QGM as query sequence following the scheme described in methods. The interacting group contains all four dimeric C2a-type HAD Hydrolases (3QGM, 1ZJJ, 1YV9, 2OYC), and the non-interacting groups contains all 11 monomeric C1-type HAD Hydrolase group (2NYV, 2HSZ, 2HI0, 2AH5, 4EX6, 3MC1, 3D6J, 3KBB, 3KZX, 2HDO, 3SD7). The AUC of ROC for prediction is 0.756 (Additional file 1: Figure S4). With the SH value cut-off <0.2, our method identifies 85 positions, including 21 binding sites out of all 31 interface sites: Recall 67.7 %, FPR 27.7 %, Precision 24.7 %. Fig. 6 shows the interface and predicted interface sites in the structure. The binding site is clearly delineated by the amino acids correctly predicted to be interacting.

The second example is Amino-imidazole riboside kinase (1TZ6), which has a homodimeric structure with one active site per monomer. The active site is covered by a lid which is supposed to be a morphological marker for evolution within the ribokinase superfamily [3, 56]. The homodimeric structure is formed through lid-to-lid interactions [3, 56]. The query monomer sequence found is 2ABS, adenosine kinase, another member of the ribokinase superfamily which can be active as a monomer. We identify 53 positions and detect 13 interacting sites out of all 24 binding sites below the SH value cut-off 0.2: Recall 54.2 %, FPR 14.7 %, Precision 24.5 %. The AUC of ROC reaches 0.746 (Additional file 1: Figure S4.)

## Discussion

Interaction site prediction using sequence information alone remains a challenging problem. It is particularly important in the context of increasing protein sequence data and given the relative paucity of structure information which always needs expensive and time-consuming experiments. We demonstrate that sequence specificity information from interacting proteins and their non-interacting homologs is able to detect interaction sites. To the best of our knowledge, this is the first time that predicting interaction sites using subfamily specificity by including non-interacting information is performed at a scale beyond a few protein families.

Our results show that prediction is well beyond random: The SH signal is able to obtain ROC values greater than 0.6 AUC. The AUC increases to 0.7 if the dataset is filtered on sequence length and HSP-length between interacting and non-interacting groups. HSPs are formed by BLAST local alignments based upon residue similarity, thus residues should be similar. However, this goes against looking for differences between two subgroups, which is the hallmark of SH scoring. Since shorter HSPs should have more similar alignment positions to get a high-enough BLAST score, longer HSPs may contain less similar positions; i.e., that differ more between the subgroups, here implying interface residues that confer interaction specificity. Additionally, alignments comprising shorter HSPs contain larger regions of higher divergence, which will likely lead to more false positive predictions.

For our analysis, on average only 25 homodimer and 14 monomer sequences were used in a group. Furthermore, we do not lose prediction performance with low numbers of sequences, above the minimum of 5 required in our analysis. This is a vast improvement to covariance-based methods that require an estimated five times more sequences than the length of the alignment to detect interacting sites. Thus, this opens up the possibility of deriving interaction signals from genomes with little sequence data available, or from sparsely sampled protein families.

We observe that conservation of interaction sites is indistinguishable from other surface sites (Fig. 2), which corroborates observations by others (e.g., [7, 28]). This also helps explain why predicting interaction sites only using sequence conservation information still remains a very difficult problem. We also observe low correlation (R = 0.22) between AUC of ROC plots and sequence length of query homodimers, which suggests that the size of protein and its interface are unimportant factors in interaction. That is similar to what Dhole et al. [10] reported recently.

The definition of 'non-interacting' in our manuscript is not that the monomer can not interact with all the other proteins but the monomer loses the interaction with another monomer (itself). It is reported that, the same protein involved in different interaction might have different binding sites [35]. If the monomer in our non-interacting group also binds to other proteins, the interaction sites might be different. Then, the sequence specificity between the interacting sub-group (homodimer) and monomer sub-group can still be used to pinpoint the homodimer interaction sites.

Our dataset is obtained from PISA and PDB and might include any bias which the PDB has. To test this, we map the homodimer query sequences from each group in Test-set 1 (both Complete and Selected) onto CATH superfamilies and calculate the overall performance of our method (Average area Under Curve, AUC) for each superfamily. Our Test-set 1 covers all four main classes, 62.5 % (25/40) architectures, 11.5 % (158/1375) topologies, 8 % (219/2738) superfamilies in CATH while only comprising 1593 out of 69058 (2.3 %) total proteins in CATH. Our method does not only predict superfamiles which are enriched in PDB but also those for which few (homodimer) structures are solved. For the selected dataset, the sequences map to 7 superfamilies. Interestingly, we also selected one superfamily which our method can not predict well. The results of this can be found in Additional file 1: Figure S7 and S8.

**Fig. 7:**
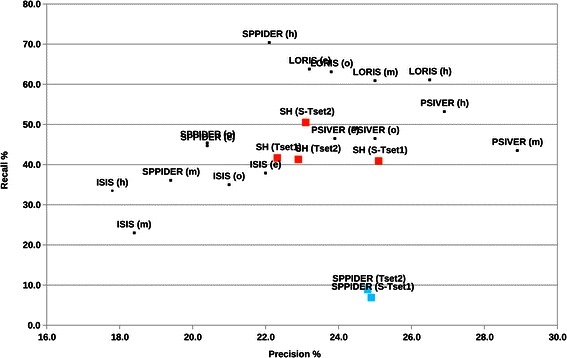
Comparison with other methods on different data. Precision versus Recall, dots in dark blue are cf. Dhole et al. [10], ‘e’, ‘m’, ‘h’ and ‘o’ stand for easy, medium, hard and overall, respectively; see Dhole et al. for details. In red, we show our method (SH) on Test-set 1 and the independent Test-set 2 (both complete and selected). In light blue we show SPPIDER on Test-set 2 and Test-set 1 selected. See also Table 1

In order to show that we exploit useful information from sequence (sequence specificity between interacting and non-interacting homologs) to predict interaction sites, we compared our results with other sequence-based methods. A clean comparison is difficult, since methods may rely on a particular way in which the data is prepared (e.g., particular database, or % identity filtering), and, in our case the inclusion of a non-interacting group. Figure 7 shows the prediction performance of other interacting site prediction methods using their own dataset from a recent benchmark [10]. Note that, due to the lack of a non-interacting group, we cannot apply our method to the Dhole et al. benchmark dataset. For comparison, performance of our method and SPPIDER using our dataset are also shown (cf. Table 1). On our dataset we achieve similar precision, but considerable higher recall than SPPIDER, and we get similar precision and recall compared to the best ones reported by Dhole et al. [10]. Overall, we conclude that our method performs well compared to counterparts, although alternative methods may perform better on individual datasets.

**Fig. 8:**
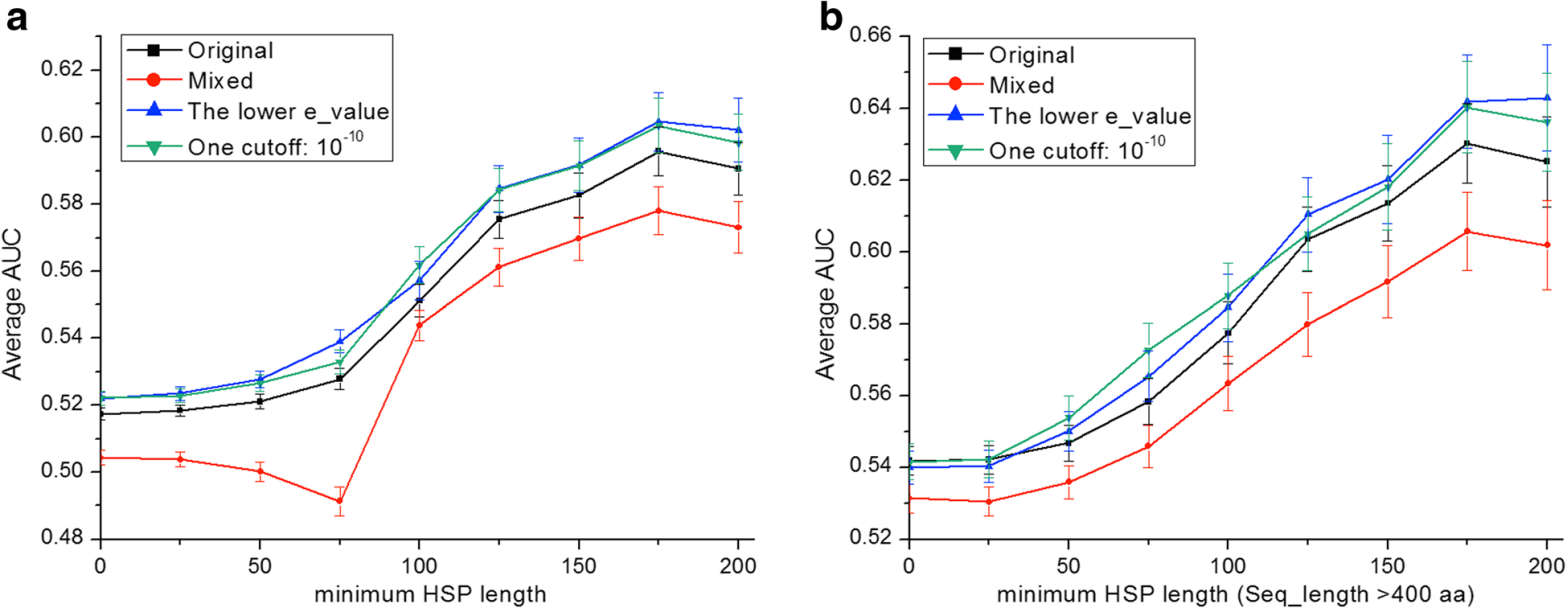
Exploring performance of different e-value cut-offs. **a** Average AUC with increasing HSP length. Each line represents a choice of setting the e-value threshold to define pair groups. 'Mixed' means pair groups mixed in at most 20 % monomer sequences to the interacting sub-group and at most 20 % homodimers to monomer sub-group (20 % homodimers, 80 % monomers); 'the lower e-value' represent using the lower e-value of the two (as described in the main text) as the same cut-off to separate H/M and M/H to form new groups. **b** Same as **a** with minimal sequence length 400. Performance seems insensitive to the threshold scheme chosen, but 'Mixed' performs slightly worse than the other three

We tested the robustness of our interface prediction to changes in the selection of homodimer and monomer sequences. For selecting sequences into either subgroup, homodimer or monomer, we use an effective e-value cut-off which is based on the first occurrence of a sequence of the other class in the BLAST results; i.e. the first monomer found for a homodimer query determines the e-value cut-off for creating this homodimer group (and vice versa). By changing this e-value cut-off we changed the selection of sequences and thereby test the robustness. We created three variations: 1) By taking the lowest of the two e-values for the homodimer and monomer groups in a pair-group, we obtain a stricter selection; this tests the effect of missing data. 2) By relaxing the threshold up to the point where 20 % of the other class was mixed in, we obtain a looser selection; this tests the effects of polluting the data. 3) By taking a fixed, strict but arbitrary e-value cut-off of 10E-10, we test the case where no complete annotation of interaction status of the sequences is available. Figure 8 summarizes the results for these three variations. For comparison, also the original selections are shown, and *p*-values for the comparisons are listed in Additional file 1: Table S7. A stricter threshold (variation 1) does not affect results, and neither does a fixed 10E-10 cut-off (variation 3). The polluted or 'mixed' data (variation 2) does yield a lower AUC, but only slightly so. This means our method for predicting interfaces is stable with respect to missing data, incorrect annotations, as well as to a particular choice of e-value cut-off. This opens up the possibility of extending the method to use a single e-value cut-off to delineate both groups, which means a full annotation of interaction status of all proteins may not be required.

Although our results demonstrate an advance in predicting interaction sites from sequence information alone, it is clear that, like other approaches, our approach also holds some limitations. First, detection of functionally conserved homologs is still a difficult problem. Thus, care should be taken in selecting homologous proteins that are likely to conserve interaction. Initially, our procedure was based on ortholog clustering databases, such as COG and OrthoMCL, but unfortunately, interaction appears to be an ill conserved property within these orthologous groups [28]. This introduces considerable noise into the specificity signal [14]. Second, for our prediction we need homologous sets of interacting and non-interacting groups. Not for all interacting proteins will one be able to identify non-interacting homologs. Proteins interacting with different partners might be used instead [14, 39], but that remains to be investigated further.

## Conclusion

We have shown it is possible to predict interaction sites out of all residues by combining sequence and group specificity information. When used as a prediction method in its current form, on homodimer versus monomer data the Sequence Harmony specificity signal yields similar precision as other signals but may obtain higher coverage.

## Additional file

Additional file 1:
**Contains all the figures and tables that supplement the descriptions of the experiment in the main text.** (PDF 1116 kb)

## References

[1] Altschuh D, Lesk AM, Bloomer AC, Klug A (1987). Correlation of co-ordinated amino acid substitutions with function in viruses related to tobacco mosaic virus. J Mol Biol.

[2] Altschul SF, Gish W, Miller W, Myers EW, Lipman DJ (1990). Basic local alignment search tool. J Mol Biol.

[3] Baez M, Cabrera R, Pereira HM, Blanco A, Villalobos P, Babul J (2013). A Ribokinase Family Conserved Monovalent Cation Binding Site Enhances the MgATP-induced Inhibition in E. coli Phosphofructokinase-2. Biophysical journal.

[4] Berman HM, Westbrook JD, Feng Z, Gilliland G, Bhat TN, Weissig H (2000). The Protein Data Bank. Nucleic Acids Research.

[5] Bouvier B, Grunberg R, Nilges M, Cazals F (2009). Shelling the Voronoi interface of protein-protein complexes reveals patterns of residue conservation, dynamics, and composition. Proteins.

[6] Brandt BW, Feenstra KA, Heringa J. Multi-Harmony: detecting functional specificity from sequence alignment. Nucleic Acids Res. 2010, 38 (Web Server issue):W35–40 10.1093/nar/gkq415 PMC2896201 20525785

[7] Caffrey DR, Somaroo S, Hughes JD, Mintseris J, Huang ES (2004). Are protein--protein interfaces more conserved in sequence than the rest of the protein surface?. Protein Science.

[8] De Juan D, Pazos F, Valencia A (2013). Emerging methods in protein co-evolution. Nat Rev Genet.

[9] De Vries SJ, van Dijk AD, Bonvin AM (2006). WHISCY: what information does surface conservation yield? Application to data-driven docking. Proteins.

[10] Dhole K, Singh G, Pai PP, Mondal S (2014). Sequence-based prediction of protein-protein interaction sites with L1-logreg classifier. J Theor Biol.

[11] Edgar RC (2004). MUSCLE: a multiple sequence alignment method with reduced time and space complexity. BMC Bioinformatics.

[12] Evlampiev K, Isambert H (2008). Conservation and topology of protein interaction networks under duplication-divergence evolution. Proc Natl Acad Sci USA.

[13] Ezkurdia I, Bartoli L, Fariselli P, Casasio R, Valencia A, Tress ML (2009). Progress and challenges in predicting protein-protein interaction sites. Brief Bioinformatics.

[14] Feenstra KA, Bastianelli G, Heringa J (2008). Predicting Protein Interactions from Functional Specificity.

[15] Gallet X, Charloteaux B, Thomas A, Brasseur R (2000). A fast method to predict protein interaction sites from sequences. J Mol Biol..

[16] Gobel U, Sander C, Schneider R, Valencia A (1994). Correlated mutations and residue contacts in proteins. Proteins.

[17] Gohla A, Birkenfeld J, Bokoch GM (2005). Chronophin, a novel HAD-type serine protein phosphatase, regulates cofilin-dependent actin dynamics. Nat Cell Biol.

[18] Guharoy M, Chakrabarti P (2005). Conservation and relative importance of residues across protein-protein interfaces. Proc Natl Acad Sci USA.

[19] Jones DT, Buchan DWA, Cozzetto D, Pontil M (2012). PSICOV: precise structural contact prediction using sparse inverse covariance estimation on large multiple sequence alignments. Bioinformatics.

[20] Jones S, Thornton JM (1996). Principles of protein-protein interactions. Proc Natl Acad Sci USA.

[21] Juan D, Pazos F, Valencia A (2008). Co-evolution and co-adaptation in protein networks. FEBS Lett.

[22] Kamisetty H, Ovchinnikov S, Baker D (2013). Assessing the utility of coevolution-based residue-residue contact predictions in a sequence- and structure-rich era. Proc Natl Acad Sci USA.

[23] Kastritis PL, Bonvin AM (2010). Are scoring functions in protein-protein docking ready to predict interactomes? Clues from a novel binding affinity benchmark. J Proteome Res.

[24] Katz C, Levy-Beladev L, Rotem-Bamberger S, Rito T, Rudiger SG, Friedler A (2011). Studying protein-protein interactions using peptide arrays. Chem Soc Rev.

[25] Kestler C, Knobloch G, Tessmer I, Jeanclos E, Schindelin H, Gohla A (2014). Chronophin dimerization is required for proper positioning of its substrate specificity loop. J Biol Chem.

[26] Krissinel E, Henrick K (2007). Inference of macromolecular assemblies from crystalline state. J Mol Biol.

[27] Lapedes AS, Giraud B, Liu L, Stormo GD, Seillier-Moiseiwitsch Fco (1999). Correlated mutations in models of protein sequences: phylogenetic and structural effects. Statistics in molecular biology and genetics Volume 33.

[28] Lewis AC, Jones NS, Porter MA, Deane CM (2012). What evidence is there for the homology of protein-protein interactions?. PLoS Comput Biol.

[29] Lichtarge O, Bourne HR, Cohen FE (1996). An evolutionary trace method defines binding surfaces common to protein families. J Mol Biol.

[30] Li W, Godzik A (2006). Cd-hit: a fast program for clustering and comparing large sets of protein or nucleotide sequences. Bioinformatics.

[31] Manning JR, Jefferson ER, Barton GJ (2008). The contrasting properties of conservation and correlated phylogeny in protein functional residue prediction. BMC Bioinformatics.

[32] Marks DS, Hopf TA, Sander C (2012). Protein structure prediction from sequence variation. Nat Biotechnol.

[33] Michel M, Hayat S, Skwark MJ, Sander C, Marks DS, Elofsson A (2014). PconsFold: improved contact predictions improve protein models. Bioinformatics.

[34] Morcos F, Pagnani A, Lunt B, Bertolino A, Marks DS, Sander C (2011). Direct-coupling analysis of residue coevolution captures native contacts across many protein families. Proc Natl Acad Sci USA.

[35] Ofran Y, Rost B (2007). ISIS: interaction sites identified from sequence. Bioinformatics.

[36] Ofran Y, Rost B (2003). Predicted protein-protein interaction sites from local sequence information. FEBS Lett.

[37] Ofran Y, Rost B (2007). Protein-protein interaction hotspots carved into sequences. PLoS Comput Biol.

[38] Pazos F, Ranea JA, Juan D, Sternberg MJ (2005). Assessing protein co-evolution in the context of the tree of life assists in the prediction of the interactome. J Mol Biol.

[39] Pirovano W, Feenstra KA, Heringa J (2006). Sequence comparison by sequence harmony identifies subtype-specific functional sites. Nucleic Acids Res.

[40] Pommier Y, Marchand C (2012). Interfacial inhibitors: targeting macromolecular complexes. Nat Rev Drug Discov.

[41] Pons C, Grosdidier S, Solernou A, Pérez‐Cano L, Fernández‐Recio J (2010). Present and future challenges and limitations in protein-protein docking. Proteins.

[42] Porollo A, Meller J, Cai W, Hong H (2012). Computational Methods for Prediction of Protein-Protein Interaction Sites. Protein-Protein Interactions - Computational and Experimental Tools. Vol. 472.

[43] Porollo A, Meller J (2007). Prediction-based fingerprints of protein-protein interactions. Proteins.

[44] Rahat O, Yitzhaky A, Schreiber G (2008). Cluster conservation as a novel tool for studying protein-protein interactions evolution. Proteins.

[45] Res I, Mihalek I, Lichtarge O (2005). An evolution based classifier for prediction of protein interfaces without using protein structures. Bioinformatics.

[46] Sander C, Schneider R (1991). Database of homology-derived protein structures and the structural meaning of sequence alignment.. Proteins.

[47] Schueler-Furman O, Wang C, Bradley P, Misura K, Baker D (2005). Progress in modeling of protein structures and interactions. Science.

[48] Sharan R, Suthram S, Kelley RM, Kuhn T, McCuine S, Uetz P (2005). Conserved patterns of protein interaction in multiple species. Proc Natl Acad Sci USA.

[49] Shenkin PS, Erman B, Mastrandrea LD (1991). Information-theoretical entropy as a measure of sequence variability. Proteins.

[50] Shulman-Peleg A, Shatsky M, Nussinov R, Wolfson HJ (2007). Spatial chemical conservation of hot spot interactions in protein-protein complexes. BMC Biol.

[51] Taylor WR, Hamilton RS, Sadowski MI (2013). Prediction of contacts from correlated sequence substitutions. Curr Opin Struct Biol.

[52] Tuncbag N, Kar G, Keskin O, Gursoy A, Nussinov R (2009). A survey of available tools and web servers for analysis of protein-protein interactions and interfaces. Brief Bioinformatics.

[53] Valencia A, Pazos F (2002). Computational methods for the prediction of protein interactions. Curr Opin Struct Biol.

[54] Wass MN, David A, Sternberg MJ (2011). Challenges for the prediction of macromolecular interactions. Curr Opin Struct Biol.

[55] Weigt M, White RA, Szurmant H, Hoch JA, Hwa T (2009). Identification of direct residue contacts in protein–protein interaction by message passing. Proc Nal Acad Sci USA.

[56] Zhang Y, Dougherty M, Downs DM, Ealick SE (2004). Crystal structure of an aminoimidazole riboside kinase from Salmonella enterica: implications for the evolution of the ribokinase superfamily. Structure.

