# Supplementary material for: Sequence specificity between interacting and non-interacting homologs identifies interface residues – a homodimer and monomer use case
Source: BMC Bioinformatics. 2015 Oct 8;16:325. doi: 10.1186/s12859-015-0758-y (PMC4599308; doi:10.1186/s12859-015-0758-y)
Supplement: Additional file 1: — Contains all the figures and tables that supplement the descriptions of the experiment in the main text. (PDF 1116 kb) [file 12859_2015_758_MOESM1_ESM.pdf]

Supporting Information to "*Sequence Specificity Between Interacting and Non-interacting Homologs Identifies Interface Residues – a homodimer and monomer use case*"

Qingzhen Hou[1], Bas E. Dutilh[2,3,4], Martijn A. Huynen[3], Jaap Heringa[1] and K. Anton Feenstra[1 §]

[1]Center for Integrative Bioinformatics VU (IBIVU), VU University Amsterdam, De Boelelaan 1081A, 1081 HV Amsterdam, The Netherlands

[2]Theoretical Biology and Bioinformatics, Utrecht University, Padualaan 8, 3584 CH, Utrecht, The Netherlands.

[3]Centre for Molecular and Biomolecular Informatics, Radboud Institute for Molecular Life Sciences, Radboud University Medical Centre, Geert Grooteplein 28, 6525 GA Nijmegen, The Netherlands

[4]Department of Marine Biology, Institute of Biology, Federal University of Rio de Janeiro, Brazil

[§]Corresponding author: K. Anton Feenstra k.a.feenstra@vu.nl

Email addresses:

QH: q.hou@vu.nl

BED: bedutilh@gmail.com

MAH: m.huynen@cmbi.ru.nl

JH: j.heringa@vu.nl

KAF: k.a.feenstra@vu.nl

Table 1. Number of pair-groups in different pair-group datasets (Test-set 1)

%identity	<100	<99	<98	<95	<90	<80	<70	<60	<50	<40
Number	1593	955	725	665	638	566	523	454	334	230

Table 2. Number of pair-groups and predicting performance (AUC) after filtering on minimal HSP length (Test-set 1)

HSP-length	≥0	≥25	≥50	≥75	≥100	≥125	≥150	≥175	≥200	≥225	≥250	≥275	%iden
AUC	0.517	0.518	0.521	0.528	0.551	0.576	0.583	0.596	0.591	0.593	0.599	0.599	
Number	1593	1525	1053	647	317	206	152	124	110	67	54	38	<100
AUC	0.517	0.518	0.519	0.525	0.556	0.587	0.618	0.64	0.647	0.638	0.639	0.639	
Number	955	915	619	395	150	93	56	42	32	18	13	10	<99
AUC	0.524	0.525	0.53	0.543	0.559	0.583	0.617	0.642	0.65	0.643	0.645	0.645	
Number	725	686	436	242	138	88	54	42	31	17	13	10	<98
AUC	0.525	0.526	0.531	0.546	0.557	0.582	0.617	0.641	0.652	0.644	0.649	0.648	
Number	665	623	400	220	125	79	48	37	26	12	8	5	<95
AUC	0.522	0.524	0.527	0.541	0.56	0.582	0.616	0.637	0.643	0.635	0.645	0.642	
Number	638	596	384	211	120	75	45	37	26	12	8	5	<90
AUC	0.522	0.523	0.527	0.546	0.565	0.59	0.625	0.645	0.646	0.637	0.645	0.642	
Number	566	529	345	190	105	67	41	33	25	12	8	5	<80
AUC	0.524	0.525	0.53	0.544	0.564	0.593	0.628	0.65	0.65	0.636	0.645	0.642	
Number	523	483	312	177	92	57	37	30	23	12	8	5	<70
AUC	0.524	0.526	0.531	0.547	0.565	0.595	0.63	0.644	0.645	0.636	0.645	0.642	
Number	454	418	268	153	81	50	32	27	22	12	8	5	<60
AUC	0.525	0.528	0.533	0.546	0.57	0.597	0.621	0.646	0.651	0.642	0.676	0.672	
Number	334	306	197	108	55	31	23	17	12	8	5	3	<50
AUC	0.524	0.526	0.527	0.529	0.54	0.572	0.606	0.623	0.637	0.637	0.681	0.679	
Number	230	209	129	64	27	13	8	7	4	4	3	2	<40

Table 3. Number of pair-group and AUC after filtering on minimal HSP length and minimal sequence length (> 400) (Test-set 1)

HSP-length	≥0	≥25	≥50	≥75	≥100	≥125	≥150	≥175	≥200	≥225	≥250	≥275	%identity
AUC	0.542	0.542	0.547	0.558	0.578	0.604	0.614	0.63	0.625	0.68	0.681	0.678	
Number	380	371	284	189	130	99	74	60	52	20	17	10	<100
AUC	0.534	0.534	0.542	0.561	0.592	0.613	0.661	0.673	0.687	0.676	0.672	0.669	
Number	198	194	147	91	56	43	24	21	16	6	3	2	<99
AUC	0.535	0.535	0.544	0.559	0.584	0.601	0.66	0.678	0.695	0.684	0.672	0.669	
Number	181	177	136	85	53	41	23	20	15	5	3	2	<98
AUC	0.54	0.54	0.547	0.564	0.59	0.608	0.659	0.675	0.695	0.684	0.672	0.669	
Number	171	165	125	78	48	38	23	20	15	5	3	2	<95
AUC	0.533	0.534	0.541	0.556	0.591	0.606	0.651	0.671	0.688	0.683	0.662	0.653	
Number	165	159	119	75	47	38	23	20	15	5	3	2	<90
AUC	0.541	0.542	0.549	0.571	0.611	0.628	0.675	0.694	0.694	0.684	0.662	0.653	
Number	140	136	103	63	37	30	19	16	14	5	3	2	<80
AUC	0.541	0.542	0.551	0.574	0.621	0.639	0.677	0.687	0.688	0.68	0.662	0.653	
Number	121	117	87	57	32	25	17	15	13	5	3	2	<70
AUC	0.545	0.546	0.561	0.592	0.637	0.658	0.687	0.685	0.683	0.68	0.662	0.653	
Number	102	97	69	43	27	21	15	14	13	5	3	2	<60
AUC	0.532	0.534	0.551	0.588	0.638	0.636	0.659	0.67	0.672	0.674	0.662	0.653	
Number	77	75	52	28	17	15	11	9	8	4	3	2	<50
AUC	0.512	0.511	0.506	0.498	0.485	0.485	0.489						
Number	54	51	31	12	2	2	1						<40

Figure 1. The different trends of SH scores at three kinds of positions with increasing minimal sequence length (0 to 600) of homodimer query sequences. 0 means sequences which have longer than 0 sequence length (i.e., all sequences). The SH of interacting sites was lowest and remained rather stable with increasing minimal sequence length. **(Test-set 1)**

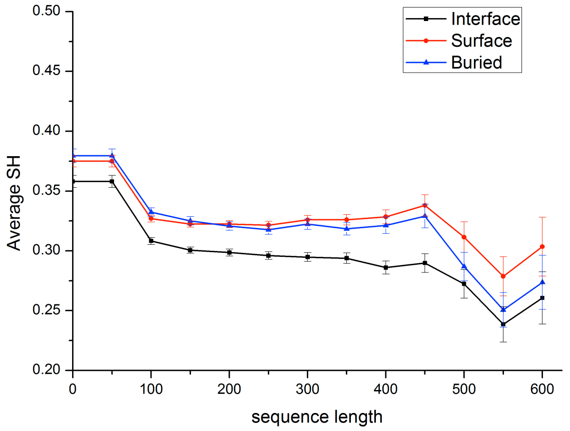

A **B**

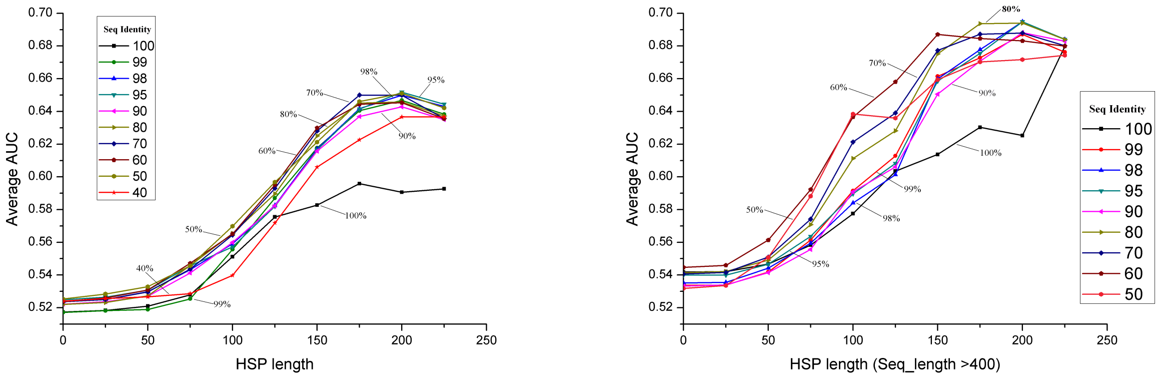

Figure 2. (**A**) Average AUC with the increasing HSP-length from 0 to 225. Each line shows one dataset with identity cutoff. (**B**) Average AUC versus HSP-length and minimal sequence length 400 for datasets with different identity cutoff. The AUC with the dataset filtering at 40% is not shown because a limited number of groups remain. **(Test-set 1)**

Figure 3. ROC-plots measuring performance of interface prediction at different HSP-length with minimal sequence length 400 (<80% ID dataset). The diagonal line represents a random prediction. **(Test-set 1)**

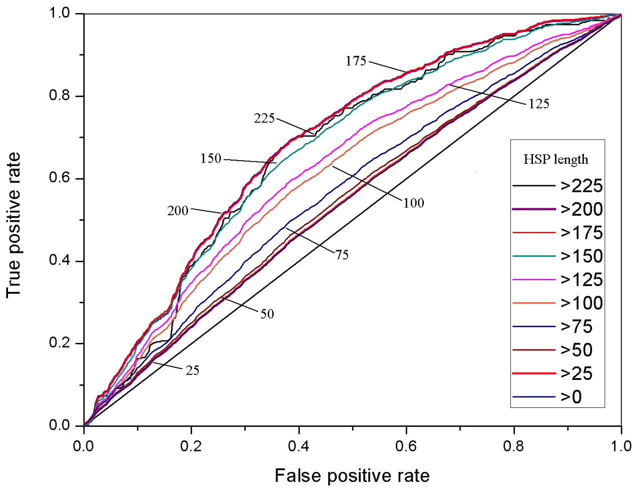

Figure 4. ROC-plots measuring interface prediction performance of the two best scoring proteins in our dataset. The diagonal line represents a random prediction.

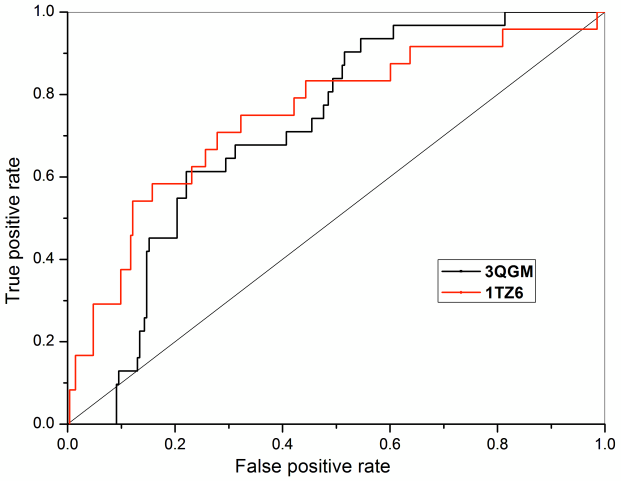

Figure 5. Exploring performance of different e_value cut-off dataset. For example, '-10' means 10^{-10}. 'Mixed' means pair groups mixed in at most 20% monomer sequences to the interacting sub-group and at most 20% homodimers to monomer sub-group (20% homodimers, 80% monomers); 'the lower e_value' represent using the lower e-value of the two as the same cut-off to separate H/M and M/H to form new groups. The x-axis represents the minimal HSP-length. **(Test-set 1)**

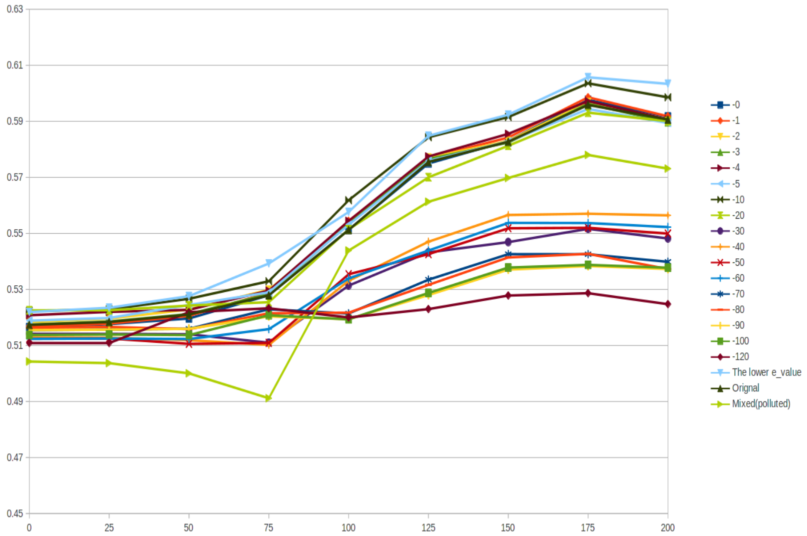

Figure 6. Exploring performance of different e_value cut-off dataset. For example, '-10' means 10^{-10}. 'Mixed' means pair groups mixed in at most 20% monomer sequences to the interacting sub-group and at most 20% homodimers to monomer sub-group (20% homodimers, 80% monomers); 'the lower e_value' represent using the lower e-value of the two as the same cut-off to separate H/M and M/H to form new groups. The x-axis represents the minimal HSP-length. All the datasets should have a sequence length longer than 400. **(Test-set 1)**

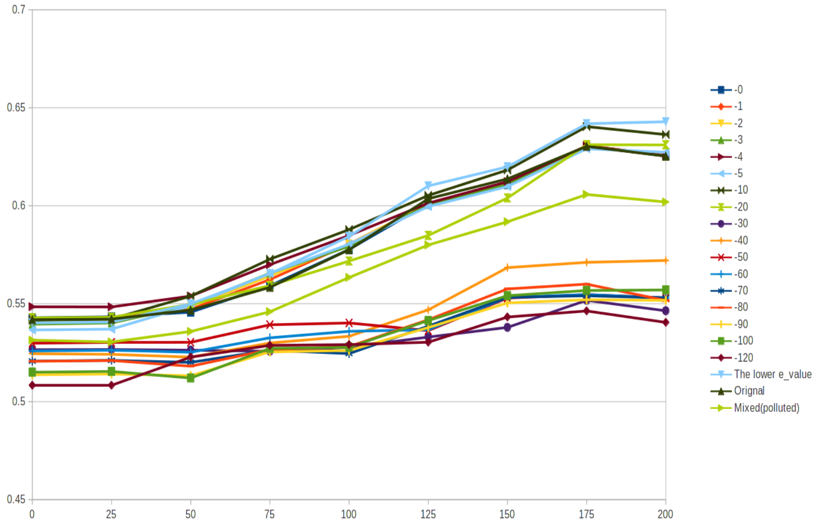

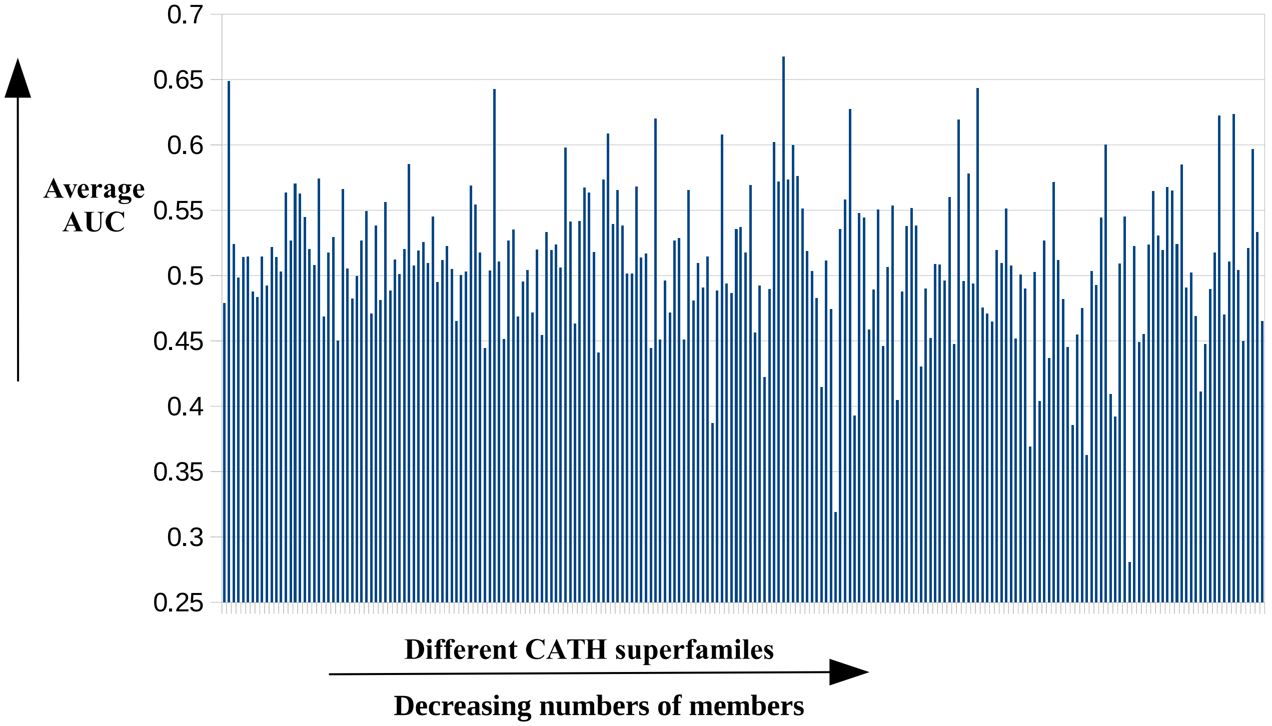

Fig 7. Average AUC of members in per CATH superfamily (Complete Test-set 1) The various CATH superfamilies are ordered by Decreasing abundance.

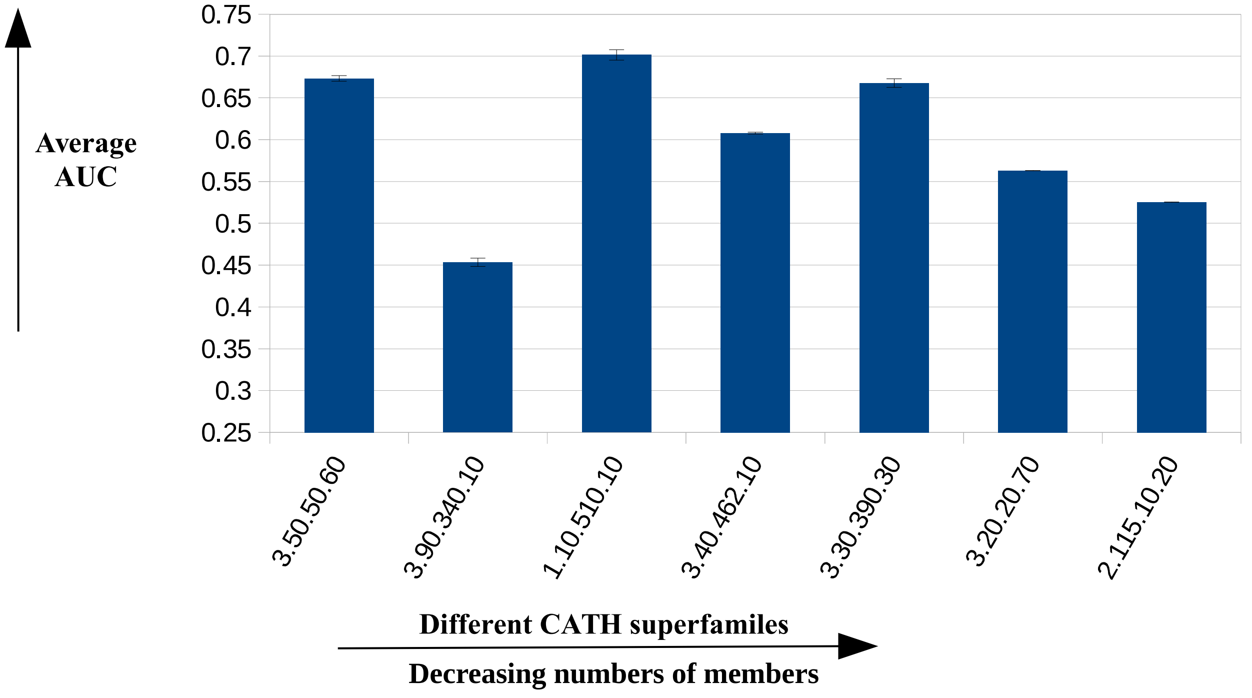

Fig 8. Average AUC of members in per CATH superfamily (Selected Test-set 1) The various CATH superfamilies are ordered by Decreasing abundance.

Table 4. Number of pair-group and performance of different e_value cut-off dataset. (All dataset are filtering on minimal HSP length.) (Test-set 1)

Hsp-length	0	25	50	75	100	125	150	175	200	Dataset*
AUC	0.517	0.518	0.52	0.528	0.551	0.575	0.583	0.598	0.592	e_value < 0
Number	1528	1463	1030	636	312	205	151	123	110	
AUC	0.517	0.518	0.521	0.529	0.554	0.577	0.584	0.599	0.592	e_value < -1
Number	1422	1366	971	601	294	199	150	122	109	
AUC	0.518	0.52	0.523	0.53	0.554	0.577	0.583	0.597	0.59	e_value < -2
Number	1359	1307	936	583	285	193	148	121	108	
AUC	0.517	0.519	0.521	0.528	0.553	0.577	0.582	0.596	0.59	e_value < -3
Number	1309	1263	903	570	280	191	147	120	108	
AUC	0.521	0.522	0.523	0.53	0.554	0.577	0.586	0.597	0.591	e_value < -4
Number	1246	1204	862	548	271	188	144	119	107	
AUC	0.519	0.52	0.524	0.529	0.553	0.576	0.583	0.594	0.589	e_value < -5
Number	1197	1160	843	538	268	187	143	119	107	
AUC	0.522	0.523	0.527	0.533	0.562	0.584	0.592	0.604	0.599	e_value < -10
Number	1055	1030	767	490	248	174	133	111	102	
AUC	0.523	0.523	0.524	0.525	0.551	0.57	0.581	0.593	0.59	e_value < -20
Number	802	794	595	389	181	129	101	87	82	
AUC	0.514	0.514	0.514	0.511	0.531	0.543	0.547	0.552	0.548	e_value < -30
Number	630	625	467	313	124	83	62	53	50	
AUC	0.513	0.513	0.512	0.51	0.533	0.547	0.557	0.557	0.556	e_value < -40
Number	544	541	408	287	109	74	55	53	50	
AUC	0.512	0.512	0.511	0.511	0.535	0.543	0.552	0.552	0.55	e_value < -50
Number	500	498	374	269	97	72	55	53	50	
AUC	0.512	0.512	0.512	0.516	0.534	0.544	0.554	0.554	0.552	e_value < -60
Number	423	421	328	234	90	68	53	51	48	
AUC	0.515	0.516	0.516	0.523	0.521	0.533	0.543	0.543	0.54	e_value < -70
Number	258	256	178	98	68	49	38	36	33	
AUC	0.516	0.517	0.516	0.521	0.522	0.532	0.541	0.543	0.537	e_value < -80
Number	240	238	166	89	63	46	35	33	31	
AUC	0.515	0.516	0.516	0.52	0.519	0.528	0.537	0.538	0.537	e_value < -90
Number	208	206	145	85	61	44	35	33	31	
AUC	0.514	0.514	0.514	0.521	0.519	0.529	0.538	0.539	0.538	e_value < -100
Number	192	191	135	81	60	43	34	33	31	
AUC	0.511	0.511	0.522	0.523	0.52	0.523	0.528	0.529	0.525	e_value < -120
Number	136	136	99	58	43	32	27	26	24	
AUC	0.522	0.523	0.528	0.539	0.558	0.585	0.592	0.606	0.603	The lower e_value
Number	1216	1175	829	486	247	167	128	109	96	
AUC	0.517	0.518	0.521	0.528	0.551	0.576	0.583	0.596	0.591	Orignal
Number	1593	1525	1053	647	317	206	152	124	110	
AUC	0.504	0.504	0.5	0.491	0.544	0.561	0.57	0.578	0.573	Mixed(polluted)
Number	1593	1525	1053	647	317	206	152	124	110	
*e.g. -10 means e-values are lower than 10^(-10)										

Table 5. Number of pair-group and performance of different e_value cut-off dataset. (All dataset are filtering on minimal HSP length and minimal sequence length ≥400 aa) (Test-set 1)

Hsp-length	0	25	50	75	100	125	150	175	200	Dataset*
AUC	0.543	0.543	0.546	0.559	0.578	0.601	0.611	0.631	0.626	e_value < 0
Number	376	367	281	186	128	99	74	60	52	
AUC	0.542	0.543	0.547	0.562	0.58	0.601	0.611	0.631	0.626	e_value < -1
Number	359	350	271	181	128	99	74	60	52	
AUC	0.541	0.541	0.549	0.564	0.58	0.601	0.611	0.63	0.625	e_value < -2
Number	352	343	268	179	127	98	74	60	52	
AUC	0.54	0.54	0.549	0.566	0.579	0.601	0.611	0.63	0.625	e_value < -3
Number	342	333	261	174	127	98	74	60	52	
AUC	0.548	0.548	0.554	0.57	0.585	0.601	0.612	0.63	0.625	e_value < -4
Number	331	322	250	169	124	98	74	60	52	
AUC	0.537	0.537	0.549	0.566	0.58	0.6	0.61	0.629	0.627	e_value < -5
Number	328	319	248	168	124	98	74	60	52	
AUC	0.542	0.542	0.554	0.573	0.588	0.605	0.618	0.64	0.636	e_value < -10
Number	293	285	227	156	118	93	70	56	51	
AUC	0.543	0.543	0.55	0.559	0.572	0.585	0.604	0.631	0.631	e_value < -20
Number	214	208	164	115	83	63	49	39	36	
AUC	0.527	0.527	0.526	0.526	0.528	0.533	0.538	0.552	0.546	e_value < -30
Number	169	165	127	83	54	36	24	18	16	
AUC	0.524	0.524	0.523	0.53	0.533	0.547	0.568	0.571	0.572	e_value < -40
Number	140	138	101	71	48	31	19	18	16	
AUC	0.53	0.53	0.53	0.539	0.54	0.536	0.553	0.555	0.553	e_value < -50
Number	125	124	89	62	42	29	19	18	16	
AUC	0.526	0.526	0.525	0.533	0.536	0.537	0.553	0.555	0.553	e_value < -60
Number	116	115	80	56	40	27	19	18	16	
AUC	0.521	0.521	0.52	0.526	0.525	0.539	0.553	0.554	0.553	e_value < -70
Number	107	106	74	51	37	25	19	18	16	
AUC	0.521	0.521	0.518	0.527	0.528	0.542	0.558	0.56	0.552	e_value < -80
Number	100	99	69	48	36	25	19	18	16	
AUC	0.514	0.514	0.513	0.525	0.526	0.538	0.55	0.552	0.552	e_value < -90
Number	90	89	61	47	35	24	19	18	16	
AUC	0.515	0.515	0.512	0.527	0.528	0.541	0.554	0.557	0.557	e_value < -100
Number	88	87	59	45	35	24	19	18	16	
AUC	0.508	0.508	0.523	0.529	0.529	0.53	0.543	0.546	0.54	e_value < -120
Number	66	66	42	32	23	18	13	12	10	
AUC	0.54	0.541	0.55	0.565	0.584	0.61	0.62	0.642	0.643	The lower e_value
Number	340	336	258	172	122	96	73	59	51	
AUC	0.542	0.542	0.547	0.558	0.578	0.604	0.614	0.63	0.625	Orignal
Number	380	371	284	189	130	99	74	60	52	
AUC	0.531	0.53	0.536	0.546	0.563	0.58	0.592	0.606	0.602	Mixed(polluted)
Number	380	371	284	189	130	99	74	60	52	
*e.g. -10 means e-values are lower than 10^(-10)										

Table 6. Student's t-test and p_values between One cutoff and Original in Fig.8.A (main text)

hsp_length	*t*_score	*p*_value
0	1.8223	0.06854
25	1.6937	0.09046
50	1.5317	0.12579
75	1.1365	0.256
100	1.4775	0.14013
125	1.0263	0.30542
150	0.8797	0.37978
175	0.7079	0.47971
200	0.6791	0.49785

Table 7. Student's t-test and p_values between Mixed and Original in Fig.8.A (main text)

hsp_length	*t*_score	*p*_value
0	-4.6857	3E-006
25	-5.0182	6E-007
50	-5.7404	1E-008
75	-6.8154	1E-011
100	-1.0443	0.29672
125	-1.7696	0.07754
150	-1.387	0.16648
175	-1.7441	0.0824
200	-1.55	0.12257

Table 8. List of PDB IDs of all homodimers (Test-set 1)

2RE7	2JEV	3NVR	3C5Q	3GUQ	2PSU	1KNU	1FWX	3FI9	3SLF
3M8F	3I4B	1FFL	3CZ1	1YAV	3O5C	3OGH	1C4K	4FZ2	1CDC
2HOE	3RYR	3KO0	2DLD	2FBQ	1I49	3IIR	1XJJ	2NRK	1BU3
3ZYV	2H8O	2VEZ	2Z69	4H51	4JBY	4EC4	2ZW2	3RK0	1LBQ
1QLW	3ZFL	1MKA	2W4M	1PKV	2GSL	1BQA	1ZOO	1H79	4FHZ
2OKK	1S7H	3VUB	3C4U	4F4J	2RGV	6ADH	3KBL	2XD5	3LO3
2A4C	4JED	4DJ3	2CCD	2BDT	3G0I	3PYI	3USF	2GDR	3DKA
3O76	3JVW	3DZD	2PCG	1DMN	3VQR	1V13	2ZN9	3GK1	3BQR
1T3Z	3H2P	2CGF	2AYU	2PZZ	1ZMC	1G60	1J22	4H6U	1YSJ
3ERF	1XOI	1VKC	2O7P	1WSW	1YO3	2Q3L	4HJR	1SMQ	2QKO
3KW1	2ZVC	2P3E	1VGP	3AYJ	1VGW	4ISS	2O5N	3QFW	1Z84
4E8E	1BXK	3D0W	3E7O	2Y51	1BEC	4IKB	2CUY	1ZOS	3BVB
1HW7	4EU1	1HT5	3OCI	4HVC	3B12	3M9F	3PJ6	1JYS	4EO4
3ASR	1EOH	2H9F	2RHM	3HOA	4HAE	3F6V	3MY7	4F0L	4BI5
2Z3C	1JWX	2FGY	2DKJ	1TPH	2C2I	3OQD	2IVV	1B15	3PWE
1H3F	3H9P	3DPT	1GGO	2ETR	4G8N	1CQK	3CKV	2OWD	3K9G

2H98	2VW8	3TSA	3KFC	2ZI6	1FSY	2Q0Y	2EN5	3C4I	3H03
1KCX	2I5B	1T9K	3F40	1CBY	1ZTD	1SZR	1WAB	1EVU	1AFS
3C8G	4GZ8	3I3G	2P2X	2X69	2HSA	1C6Z	3T32	3A6Q	3K9D
3TB6	2H84	3EZG	2JD3	3K2O	3BPK	4GP7	3VP5	2BZ1	1O4S
2FU3	1GY7	1UJN	1QRD	2VS0	4E0H	2IV3	3NW2	3L31	3N3D
1YNH	3SMQ	1UQ5	4FKC	1X83	2Z3O	1GSE	2EL9	2XJ9	1BJF
3AJX	3NDO	2ED3	1X8D	9WGA	1YBX	3HM4	1XET	1GXR	3HE0
1X6I	3F1L	3UUC	4HF7	3LC0	2VPF	3STY	3I7R	1VPI	3KCX
3D5T	1HOZ	3MF0	1DEK	3CP2	4ESX	1A64	3U48	2AUR	3FBU
4GPA	2BJO	1YDW	1BO8	2H3G	2DP3	4HZO	4IN1	4HIA	3HHE
3GJU	3K87	2UV1	3PR8	1VGR	2AJ9	3NYY	3MGK	3TR0	3QNU
1NXM	2QV2	1YWQ	3O2R	3Q63	2V5M	3N3S	3B8X	1KBL	1ZGA
4GA6	1SKZ	2IKQ	3JVD	1K49	3CWN	3L1W	3TAT	1DQZ	2F6X
2QFI	3I6J	1UB0	2I9X	3QDE	1OCV	3FDG	3CKA	3LM2	1PSQ
2VTK	2YUT	1V94	2C72	3R6F	3PDX	1BY9	2UVK	3FSE	1VZZ
2J66	1U7H	3OIP	3TEQ	3IA6	2BKB	2OTP	4BET	1WSR	2VJN
3P8D	2F5I	3RUF	1GOI	1U08	3G1O	3UYL	2ZJZ	3BNI	3O60
1TXT	3U2L	3IJ6	2Y6W	1GGG	3URR	1V47	1XQB	1U6Z	3LNE
4ATV	4HOJ	3OA7	1XCV	2D66	1K4Q	4GBD	2HTD	2AN0	1QW7
3QGM	4HCG	2FQM	2EUI	3G3F	2Z2A	1YM5	2R2F	3JU7	1DPZ
1DBT	3KB1	2PN2	1RK4	1U5O	2HP3	3HWK	2FFJ	3PD9	3VLH
4EW6	3C70	2AB0	4DUQ	3HV2	1JXN	4AH6	2QFN	2GAK	5DAA
1ICU	4AP8	2IFC	1XUA	2WTH	4RHN	3HGV	1ZYD	4FL0	3MWS
2PWP	1K41	1TED	2XEC	1HJ1	2ZHH	3LR8	3GYB	1X2I	2R78
3V75	1W2Y	2P53	4GAH	3UPL	4EAF	1ID1	1IGX	1P18	2IU6
4EF8	2Y7P	2Z4B	1LAY	2AV3	4LC8	3BWV	2FPS	1E3U	3C3J
3BDD	3RBY	3A2B	1S4N	1LWD	1B0W	4IW9	2I8T	3A9X	2B9U
3UJS	3UKM	3BJX	1CTN	1O6A	1AXE	2YVU	2GT8	2X7X	3W01
3PMR	1ZP8	1BQ1	2PHT	3E5X	2QQA	1FD9	3OXV	3Q34	4JNH
3BLN	2PC6	1EA9	3RW9	2Y3H	3N68	2PG3	1EEM	2X9E	3IHT
3P1Y	2P7O	2BOL	3H0S	2G3B	3UX3	3K1T	3PRH	1AZR	3QQA
1VQG	3FF3	3SL7	1L5B	1U3Y	3C4W	2P1J	3OGP	2Q1K	1E9Q
1R8K	2O1S	3F8C	4DYD	4IBP	2CWZ	1AE2	3SZT	3HL3	2EBE
3LGN	1H1W	3RM5	3NBE	3OHE	4HSH	2WS2	2EAV	2D4G	1VQW
4B0C	2Z04	2FIP	1BOW	2IA0	4DS3	2CA8	2ZU2	3IE7	2GA0
1OMO	4FU0	2UXH	1D7C	1FTX	3EFY	1XSQ	1PY9	3QC3	3QTY
2WYT	3NDU	2JE2	3NYD	3ICU	2ZN8	3KSH	3KIA	2IMM	1E7Q
3NRH	1XDB	2DSJ	3KY7	1XR4	1E7Y	2VZX	3CTY	3LAD	3H90
1DXQ	2DG7	3AWH	2VSH	1YBT	1J7G	3S5R	1DWX	4FVF	1E9O
1M38	4F22	4UPJ	3ZGD	1TYP	1JK6	2QGY	4DF1	3ATH	1NPD
3R89	3ROW	3BXZ	3OP6	1KU9	3IRV	2WCU	2BTZ	3FFM	3L07
2HMA	4HVK	3I4U	2RG7	4A20	2WYI	2C11	3NOY	2EC2	3FJS
3RZZ	1OAL	2FCA	3TV8	3GET	3RCN	2WK1	2IEN	1OSJ	3LPX
2YG5	2NNN	2O8I	1MRX	1LBK	3GWK	3E07	1NI5	4JQT	2AO2
4AZ9	1BQ6	1XA4	1V9N	2RJT	1H0K	1JSC	3K28	7HBI	1QWI
3BOF	2GLZ	3NL9	3B1Q	2NS2	1KLZ	3QSQ	1PRX	1RZN	1SD4
1MC5	3PWI	2WNT	3LUF	1H2H	3AFJ	1J8B	1AXA	2Z1W	1BQD
2IXA	2A8L	3ZUX	2HGW	3PDB	2OZJ	1ISO	3EZ7	2ZXY	1A59
3SB1	1T3B	1DP4	2FBI	3TGW	1ORD	1DXL	3M44	2YQU	3W6W
2R11	4EU9	3GQF	3ORR	2CWF	1KM5	1I86	4B4P	3AJY	3E4F
2I7N	3U5S	2E1Q	3LZZ	3B76	1VPZ	1TZ9	1OQC	1UDA	3KIZ
3SG5	3IT2	1X04	3OPC	1HSS	4B9D	3LLX	4KQW	2G86	2EJA
1J0C	3H3L	1RHC	3ISZ	1F3A	1J0E	3S53	3KKJ	3I2I	1M6N
1JLX	1S9C	2Q6Q	1HKU	2RK4	3I51	1TMX	1QMH	1ZKQ	1WL2
3CLK	3HNZ	2RGS	1Y9W	3NF2	2XTI	1BH5	3DLR	3FNB	3LK0
3UR2	1UPI	4FCJ	3PQ1	2EH6	3CXZ	2ZZT	3F8M	1QF5	3ENC
3R6A	3G8R	2ZCG	1TPU	1TC5	3MGG	3IC7	3L1G	1NF8	3TP9
3CMR	3TZI	3F1C	1B7B	1VI6	2ED5	3TRC	1CM9	1TDF	3FAS
1CGN	4GPN	3ZVH	3ECY	2Q3X	1AWB	3AXB	3DAA	3OZ6	2IJI
3IB8	3VOD	2XLT	1JC5	3MD7	2YV3	3MQ0	3DGO	2AWA	3QWU

1TCD	2DG8	3R0U	3L09	3JU5	2OER	3G2B	3LN6	1JHZ	3TQP
1FHE	2C7W	1CM7	2PZ5	4E3R	1LWI	2NVV	1WD8	3FU1	2C3Y
2RAE	3FWZ	2OX1	4GBU	2Q6R	2DSA	3B46	3L2J	3Q64	1VLS
4EW7	2VK8	1PRE	3DGQ	2NVM	1SQ5	3EE2	3MZ2	2B8W	1GK6
2YY8	2W93	3FYB	2XH1	3DYT	1OZT	1E7S	4FMM	2E5X	1IHK
3VVM	1O57	2ZCM	3OAO	3E7Q	1QGD	1Z0X	3QSR	2FZW	4H17
3M3I	3FGP	3M3W	1JM6	3GAY	2C4I	3AQT	3DP9	2OZV	3B47
3R03	3OPB	4E1N	3Q2J	4K25	1M54	2IJH	3HCW	3RIT	1QYA
4F6A	1UC8	2VRZ	3T61	3UBR	1YNI	2Y8H	1GS3	3LM7	2HLJ
3SMP	1QLH	1EVX	3G22	1FBU	2WKO	1I0H	2IUT	2Q9K	4IXN
4AS5	4K8F	1ASK	4DQ1	3THC	2PN0	1IUG	4E1O	3TIV	4G1O
3RH9	1ETQ	1OR6	4DZ8	2IOQ	4G89	4HN0	3PMD	1K86	2ZJX
3LXX	2SAM	2ON3	2UV2	2B5V	3MZV	3CJW	4FP4	3EVJ	4A5S
4ERU	3U13	1V71	3EFB	1JB5	1XSR	2Y5Y	1K3R	2PH1	3RHC
2VEQ	4GO7	1SPQ	1DJ3	1JQW	2Y70	1XKJ	1P0C	3ORF	3H51
3ICV	1JUD	2Q2F	2YFB	4F9K	2FV7	1QAL	3OXA	1DJU	3UHZ
3FDB	2C5E	4EG0	2GE3	4DJ4	1BET	3SC4	3RD1	3DO3	3SW1
2F1M	2Z17	3IKV	2QUF	1LVE	3LW3	4DSR	2FYQ	2PYW	4ESB
4EHS	1ORE	1H0N	2ZX3	1V43	3K2V	1WX1	2YXZ	3A05	3MEN
3LYC	1TYB	2AJR	3BXR	4ETJ	3GBX	1HZW	3T7D	1LOS	2TOD
3EUQ	2HBO	2I9Z	2JK2	4ACD	3ICT	2C9H	3KRV	3MSH	1BLC
1HSO	3VCB	2Q4N	2UV0	4HPK	2QBV	3KZU	1GVE	2XBN	2OYN
3ZCG	1QOM	2QH1	3ACA	3KD2	2XYB	1DJ0	4DL9	2FDE	2QPV
2EKB	3FIU	1VLL	4EKY	2HZ6	1ZV1	1JJK	1R2R	1GRC	3QTA
1GFS	4TS1	3OX9	3ERJ	2QSX	2Q1E	2QYM	3THN	2VEB	1KH7
3DSB	1LOQ	3RMU	4EG2	1Q7E	1R6U	3V67	2AMP	1G1Y	2ZEJ
3S67	1PZS	1ISM	1OZU	2J6R	3QAN	1TVV	3CHV	3LZK	3MPG
1TG8	2PMR	2WU9	3UEI	1UN8	2NRH	4GQT	2Y5D	2FFC	3AL6
3G3E	2XYM	2PNC	4FN3	3QLJ	1THT	2HUM	3CM4	1J3B	3F7A
3LXB	1IA9	3U3X	3QPT	3KJX	2OQO	3LL3	4DAD	3SBX	1N5T
3E82	3DNK	1CG3	1ALH	3KYA	4KUN	3SOG	1IVV	3HJ6	3HM7
3RY8	3OLP	4E2A	2DW7	3L79	3NJC	3LE4	1FW1	2XLR	3EET
4GA4	1ECS	3ZZM	3TO3	1XSO	3RMI	2OBY	1V5Y	2P62	3CSM
2PFS	1X03	3VQY	3ONP	2VVT	3CJD	3DL3	2TPR	2Y53	3FQ8
3UG9	3UW8	2VJ1	1WY5	3HJB	2XSU	1JHG	4IGJ	1GZD	4GP1
2QH0	3TQJ	3B97	2NVA	3SF0	3L3P	3QMS	1NJ6	2QL3	1K94
1TSX	4DF2	3U6W	2CTS	1EXT	2OQD	1VBM	2Z4X	1KPC	2P1R
4DYK	1YAA	1SJ5	3PM7	1CZ7	2HNL	3KF5	1SSG	4F2P	3LJL
3KPH	2VBG	1UW7	4A00	1KOF	1A4X	2IIZ	3QMW	4J2Q	1TW3
2W2K	1WC6	1SYY	3MNL	2A9U	3R4Q	3NRR	3IX7	2PN1	2AAQ
2HIN	2A4K	3ZJG	4G7A	3TOE	1UTY	3CYW	1VEV	1ETW	3CBG
3E3C	3M5X	4EO3	3ZTA	1YM3	2VVQ	4F5G	3JYN	4A8E	3FWY
2V57	1EW2	3NU6	3KWP	2P84	3LFR	2CKJ	2XDG	1THE	1VQC
2BKW	2I6R	4F5K	1K6S	2BH7	2O1T	1TY9	2ASH	3G7V	1NVT
3EGJ	2A9D	2ZJT	2EQ6	1J5P	3LKV	1D6J	1B5O	3EW0	1GVJ
1E31	1FBS	1VIX	3GZP	1MHO	3PCT	3E4V	3D0T	3LMA	3IWG
3HRS	1P5H	1CQ3	4BJ1	2IOA	2JDJ	1P62	3ZGV	1V1G	1ONO
3BLI	2H0Q	1V8Z	2OXL	2VXO	2ZYG	1IZM	3VF5	2QNV	4H2G
2WYA	1MYR	1WC5	3H7L	4F2N	3QIK	1CIV	2HZK	3NAD	3R18
4F1E	4A47	3N9X	4JXU	3HQX	1MOQ	1DZ3	2QG3	3FNS	2W7Z
1ZX2	1KU2	3VB2	4GFK	2XX5	3MAD	1AJM	1YH8	2XDP	2F1K
2DB7	1XNF	2DV1	2RIQ	2B0R	1UOM	2OEE	1HUK	2FD5	1MML
3GWH	2FLQ	1LK9	3B5M	1PS0	4A2U	2XWC	3US8	3I43	2EJZ
3UJH	1EK4	2R4I	1NZR	3DSL	4GC4	3LVA	2EQ5	3EX6	4GLL
1IAT	3C61	3FI0	1QHI	1JXH	3LUY	3GC4	1PX7	1B0A	3T1W
3INV	2RH0	3N4R	1YJS	1ZZT	1H9G	4LB8	3NAT	3M7P	3OJ7
1Y0U	1SKV	1QS4	3URN	4KK2	1MT5	2VR6	4GLT	4AEQ	3LMU
3DWJ	3IIB	1MV8	3F13	1ETK	2OWP	1A71	1B5P	3N05	1T8Q
3N9T	2APG	1VHQ	2AKC	3T1X	1XWM	1S9I	1YJ8	1S3O	3IIU
2X6H	2IMS	3KOS	1F1M	2FU4	3TG2	4G2T	1UTH	3UPS	3K8R

2PML	2IEO	3RPE	3F1V	1XB4	4HVJ	3UQZ	2YAE	2GP6	3E8S
1G6W	4HL6	4BR3	2XMS	3GJZ	2YWL	1J6V	2OIK	3HIM	2GN0
1NS2	2R18	2CWV	4AGT	3HTI	4F3A	1XDS	2R0T	2GO8	1Y9Q
1TPB	2D8E	3C8N	1KQP	1LXE	3S5T	2XU6	4DKY	2P6P	1T9D
3AQM	1M4R	2VMP	1HQV	1O6C	1Y7M	1YQC	2X65	1OBJ	3U6G
4ALB	1C39	1P7N	1TZ6	1XTM	1ADU	1UN9	3G1C	2O99	2JAS
1AJ8	4ID7	1TDE	1PDG	3PFO	3ROS	3TB4	3DM8	4DT5	1U5E
1VH4	1IBH	2QBU	2VCQ	3TK1	4HME	3AP3	3CL4	3G0K	2YAT
2PH0	3R9U	4AE8	1YDH	2Q0X	4K3Z	2GN5	2XBU	2YLZ	2PVZ
2ZA0	3ZYY	1WVF	1DK4	3LV2	4A9W	1TGK	3I0I	3VP6	3NWT
4JVU	3MBL	2HNE	1TEX	1EQB	4GQV	1KM3	3BHN	1VHD	3RPC
1E6P	3UNT	1QX5	3L84	4GT7	3KT4	3JTZ	2B0C	3EEI	3FZW
3P9T	1WLI	2QNT	3KYE	1B3N	4HJ2	3CXN	3QWB	3EA7	2YEU
3L2K	4L8E	3FRK	1AKA	3DCM	2Q12	3CXV	3A4J	3EE7	2Y6P
3RFW	3HGG	1OU5	3DEW	2P19	3IGD	3P2P	2YYU	3QAV	3OJI
4L4X	1NXZ	3MDP	3DDH	3HC2	4H2I	4AX2	1L0W	2VD2	3SSI
2RAF	4FSC	2ZHP	2XB3	3P1T	3DGZ	3LZV	4DQ8	1ZCU	2XRH
1WLT	3VVL	2X2U	2YY9	3THA	2FNP	2FUV	1MO2	2GI3	3B57
1VJT	1W6Q	3R2P	3EL9	2V54	1ZPS	2DV7	2HJS	3PQK	3VZ3
2IW2	3QM4	1YMU	1M2A	2WUQ	3UHU	1IRJ	3FC0	2OVI	4EPF
4EW5	2UV4	1TRB	1VL7	1Y92	3NI0	4FAK	2V28	2QVF	3AJW
1PFR	1O7L	2OPK	4B16	3EWB	2RAS	1J32	1CW7	1N8F	3LVM
3FE3	3BS8	1QAP	4EGE	3B4D	2GFV	2W7R	3CRJ	3F8H	3PPB
1Z82	2OEZ	4KVH	1CLI	1JS3	1A4H	3C3Y	1O9P	3Q2W	1DT0
2YZM	4HUT	1Z1E	1FZV	1TPC	3V1T	2WE8	2XPU	1F91	2W01
4IJN	1ZXZ	3HFD	4AQJ	3R38	3IJT	2X40	3NRT	3SJS	3OHS
3GIE	4ERR	3VDG	4I44	2VK1	2WSJ	3AUY	2WAG	3QVQ	1XDH
1R9J	3LPM	3UJM	3L6D	3HYL	1WPQ	1HH1	1XFS	2B7Z	3Q2N
1SMO	3D5P	1YSB	1KA2	1USP	2ZM0	2WMD	1P48	3NMD	3Q74
4AKV	2RDE	3EK3	4FXR	1KGZ	3E77	2EFL	3TAU	3H77	3KH0
3NU8	3G1T	3KEB	1PD2	3A3F	3ATP	1WLC	2QWV	4KKQ	3LRC
3MYF	4ICT	3LL9	2W7W	2BWJ	3CCK	1PX6	1ZY5	4I8A	1TC1
4G19	3GWL	3W2Z	3GY0	3LOY	3UYY	4L5P	4AFM	3DJB	3FOU
2DC1	1KVT	2O0R	4AIH	3TLE	3KB2	2YBX	3NA3	2O72	3V8H
3SMA	3G02	1U73	1MPU	2GOK	1VZA	4B5O	2HE5	1KJN	3SLC
2CFR	3SSJ	3LFH	1VKG	1K6D	3EOQ	3QH8	3PIJ	1WLE	1H4F
2ODA	2TSC	3CI6	2QZ9	3M3V	3ETJ	1VHV	3MHE	2AZK	3FC5
1YI8	4H1S	2VMT	2FFS	2VLD	1UOE	1S7Z	3COY	3QMQ	3IA1
1QB2	3R5K	1O3U	2OFY	4AE4	3H0Y	3GWE	2E4N	3E8X	1HUJ
2CWW	1AIH	2O4N	2Z98	3PGQ	3UYV	1BJN	2ZDY	4GLW	1OEP
3BJV	3ZFI	1SNY	3V1V	3UKT	1WD6	4FXP	1BIU	4E1R	1E5Z
4B4Y	3ZXO	2DTC	3OBH	4DN1	1KLY	3GTH	3OSV	3CLH	2Q20
1NJH	3FHJ	1R66	2QJ4	1R5A	3I24	2FXD	2VCV	1D7K	4DA5
3S5S	3T8U	2IK6	3SXZ	3QZY	3BFI	4ASG	4MK4	2DVK	6GSV
1ONF	3DL5	2HH9	4EWG	4EDY	3JSA	2Y6Z	3QUV	4AK7	3V4S
3GEL	4KQX	1FEA	2I0M	1B67	4A2C	3CTW	3D1C	3M1L	3APY
3ISO	1BHL	2PWN	3LLD	3PNH	1XPK	3NSK	1O6T	1J79	2IGQ
4A1I	2JAX	1FEW	4B1F	2G3A	2EJN	3FFR	1DOS	3LY8	3P4E
1KNY	2ISN	2IK0	2J3J	3HZO	3HAI	3VQD	1OAH	4ASI	1VR7
1CQS	3PI2	1U3Z	3RUO	2Z6J	3OF4	3U6U	3AB8	3KKG	4IS0
1S08	1LXD	2EFK	1DTY	3O98	3QSG	2QO1	1Q8B	4JZ7	4G3W
1HXP	2G8D	3LK5	3HUH	1WST	3O9S	1SH8	1R1U	2OAI	2VUG
2NYC	4JHC	2DBS	1TSM	3HZP	3NFT	3RQB	3EEA	2PVQ	2I87
1SIP	2IOG	1P1C	3MSU	4L82	2QH7	2OSD	2PIJ	1ZCV	1FWZ
3NKL	2XF4	2RIP	3H95	2QT8	2O2D	2X5C	3IV7	2ZGU	4GMF
1SHN	3KOT	3UT2	2RF7	3V6G	3BCM	2GK4	1UC9	1D4S	3HZ4
2EJ8	4IJ6	3LPN	3G8L	1BWR	4EB9	4F4D	1Y3T	3QZX	2PW6
2P1A	1VBN	2C1U	2Y7I	3OOY	1WTD	3LHQ	3O4V	2W7F	3MLF
3USS	1FPS	4B0I	1ERE	1YG2	4DI0	4IPJ	3CEI	2F22	2DXU
2B06	2I7G	2YZI	3MOZ	3M7I	3MVP	4AZB	2E2T	1U20	3FRC

3KMI	4GLB	1J0H	2P67	3QF0	4GDT	4A26	2OR3	2V30	4I15
2YVY	3I2H	2OE1	1RQI	1XPL	1OBL	3ACB	3TOJ	3QP1	1VZG
3EUP	4E6H	1KM2	2PBW	2I71	1AMH	1M3E	3HAF	1WCV	1EV5
1DLE	2C3D	3CLV	2YVE	2XX3	2X7Z	3A5Y	2I80	3I44	3HAH
3GC6	1BBH	3D3J	2AAW	2XH2	2H39	1TOG	3VW9	4FYK	1J0R
2G7S	3G2P	3H4Y	2GT2	2RKH	1JEH	2C8U	3UFN	2CJE	2CFB
1DJ8	3MEY	3YPI	1B57	3OVR	2F1R	1U7I	3R0Q	2OIP	2W11
3F4C	3KQ5	3L2I	1LQL	3V9M	3I0Z	1A9M	1WIW	1BIQ	4J6E
3NZR	3CO5	2IKB	2H9U	1QWJ	2ONF	4I8Q	2IMJ	1PBO	3MIZ
1XZW	3MTQ	1HRH	5FIV	3Q4G	2HYD	3UEG	4F5M	4IWN	2ALM
4I0K	3JW2	3KUZ	2XKL	4B1B	2PA7	3EZU	1LQP	4JGA	3AAJ
1EFH	2X8U	3D20	3B81	1HZ4	2A9S	1PL5	3H3Z	1MX0	2JID
3TD4	2PIN	4E16	1EKF	2ZGW	4B8X	3M52	4HTF	2QTI	2F3X
1WY0	1EH9	4JB7	3MEL	1CBF	3TB2	4AVP	2FXQ	3M9Z	3CWX
1W53	3KF3	1Q8M	1D7D	3TSY	2C4J	3RO4	3KVU	3DCF	4FK1
1V08	2F4P	1GFZ	3APN	3N6F	3HUU	2PAE	2GOJ	2F5J	1Z0P
1HXQ	1YR9	1U4R	2BV6	2GT4	2IAI	3P3I	2X50	1MZJ	4I1Y
1D2F	3UGI	2DP9	2PL5	1VCI	4FRZ	3VHR	4G2M	3IH0	1MZV
2O0L	2RL3	3PGB	1CCD	2HDH	1DCF	3U2A	3FD5	3RBX	2NWG
3TS7	2I33	3BA3	4AK9	3UDO	2BAY	1ERW	3P2Y	3GRO	3AI9
3RHT	3NEM	1X7O	3S64	1M0U	1O5L	3CZP	3T22	1R8B	4FOL
1ELX	4G06	4FTW	4DQF	2EBD	3JV9	1TXG	3GU3	1U12	3KMO
2OU6	2BYL	2ADP	1H6V	3C8J	3RPP	4ASL	4G0L	3HGX	3BN7
1MY7	1WKZ	4EM8	1YO6	3FG9	3ZWF	4JCI	1TXZ	3BHQ	1RIY
2BEK	3F0H	3M5U	2AYQ	2Z6F	4F66	3CU2	1ZO2	3PPE	2P5F
2W0R	4IJ5	2ES0	1B6S	4F5L	1XFC	1NY5	3HX9	3UEY	4IT1
3CJN	3OI7	1HJ6	3GDI	3BGX	1QQ6	1Z8D	2R2C	1HKQ	4E5P
1OPR	1PBF	2J2F	2ZCX	2WP4	3KXK	1DVI	2G8Y	2EGY	3BL6
3B60	2WK4	1B8G	1HTA	3DH9	3K96	3BCW	2JGV	1SEI	1A15
2OOI	2X0S	2V42	2FNO	4JQZ	3KCQ	3GU2	1QUP	1WDZ	1V1Q
3DLX	2GED	2YR2	2IW4	1R7L	2O08	3KDC	2X5F	4HI3	3K8E
3MOI	3U7A	2YCS	3HQ8	3T9V	4AMV	1I4S	1ZP7	3STX	2H53
1CHW	4EZG	3K3P	3TFX	1G5H	2WSQ	2E5F	2QVC	3N9I	1EOV
1YFW	1OH0	3LIQ	3KAO	2GXS	2QHQ	1HJK	2Z85	1WKH	3FMI
1J31	1TOX	3U0B	3GR9	1BWD	2OVG	3T90	1B4P	2RK6	1V5V
2NQZ	2DBR	3IPR	3NR7	1N49	3E3M	4ITB	1CI0	2J85	1JBQ
3TQD	3Q84	1JVL	1I14	1OJT	3T4E	3CBX	2JJ8	2XMT	2F8M
3MNG	3OB4	1ZNP	1TLG	1T98	3E10	3NF6	4DJN	3DU4	4HRW
4G2D	3IV3	3L7W	3IR3	2ZVX	3G5L	3FNV	1BMP	1YPI	4KEM
2GD9	3EXZ	1ODW	1FZR	1HVY	1FJH	2F9T	4L3R	3MK6	3ZQ5
3Q1Y	3F71	1US1	3SXM	2FX0	2R1U	2ODO	3Q4S	2XCK	3BW2
3OS8	1ISA	1H7H	2BMI	4G1K	2IES	3MUX	2WWI	1HGX	2AEV
4NOS	1KJX	1Y7R	4FKZ	3DAL	3V0L	2IKK	2DDH	3DR4	2CYB
2I6H	1VBH	2Q5I	3A5S	3H07	2J6Y	2GX5	2HUU	3RFB	2DPN
1F1D	3I54	2CME	4HFJ	2QSI	3A14	3MJM	2J6Z	2H2R	3LLF
2Z2B	2J1P	4FQO	1WGC	2AKZ	3I2F	2O3K	4EUD	1KEP	3BM2
1IJI	3GHF	4HX7	3ELE	3KBY	3MS5	1OOE	2XR4	1RSR	3RQI
4A1N	1WU9	4I9B	3EIP	3NVO	4DZW	3E7G	3TZL	3CZ9	3HNW
2QNU	2AVT	1ZXK	2DFU	2XWN	4B17	2QNJ	3QG0	1HEI	2DPF
2PSG	2CCK	4F5I	1MVP	2CIN	4L0Q	1VCE	3D57	3B36	3D45
1DMQ	1YGP	3SE7	1CD0	2P8I	4ECJ	1NFZ	2OI8	3BAT	1GLL
2R5P	3B38	6GSU	1ODX	1DLI	2V9Y	2DPG	1L5Y	4E04	2BHK
2ATS	4A0M	3DC1	3IC6	2HK2	3F8X	1ZVL	2A6B	2B9D	3EC6
3O8J	1PE0	1Q1E	1SZZ	3U9X	3EO4	1XUQ	3BWC	4IAR	1AOG
2O5H	4HEB	2QSW	1YR0	2CN4	2RJH	2X2A	1WPN	3DVI	3SMS
1SET	4APV	1GLQ	3S7O	3IB5	1G5C	4EC0	2WOJ	1WW1	3HIU
2JKV	4E5M	2RGU	3G1L	2OTA	1AID	3HBA	1F4M	3MTI	1RW4
1YOA	2HAY	4IO1	2Q7V	1PQU	1ZN3	3S6D	2QR5	2W2L	1SZQ
2VF7	2G6L	2JEE	2A9F	1STO	3FR7	4GYS	3C3M	2Y79	4B9E
1R3S	3LY1	3OML	2FON	3B1F	2YXN	3KCC	2FUR	1YIB	1HLM

3QTD	3OQP	3RKC	3RLU	3VEM	1C3G	3TQG	1OUL	4EGB	3C9E
3M5Z	1UT4	2WNS	1FP3	1K20	3S4D	4F32	3F6O	1D3Y	3FFL
3V8L	1R65	3GUZ	4F31	3TW2	1T4B	2GJ4	2FZT	4HJ1	1XVV
2Y7R	3LNS	3PUT	3S8C	1TLJ	2PZ0	3BDH	1M6E	2IWQ	2WGW
1ZJJ	1TV8	3SLZ	4MT2	3V7C	2OP5	3PZS	3B5T	1JQC	3HR0
3BXP	2QWW	3HME	4MDH	3N46	3RU6	1JLK	3V4C	1VQJ	1I4M
3BM6	1ORO	1UTG	3G8G	3PJY	3O0T	2V5B	2ZMA	3CQ9	1ZVP
1R3N	2QA2	3G25	2EB1	2XMH	3G26	7ODC	3Q6I	1E3I	3ICF
4GEM	2DV3	3UHN	2APS	3EZ2	1X08	4J0N	1DZT	1J5W	1KUT
1K8F	4FCX	3ZXC	3PC4	1M7V	3AX6	3E1H	2OQ2	1CBG	1JMG
3MUQ	1MJT	2Z54	1K88	3CL5	3K5H	2INR	1R4W	2QC2	2D36
2FBL	4JBD	1ZHG	2Z76	3BB8	2AZQ	1KYW	2O53	3QGZ	3DED
1H69	1Y6E	3HMQ	3CM2	2AWD	4FUN	2ZGO	3I9X	1UWH	1BTK
3MGJ	2BML	2IBP	3TYS	2J0F	2VP8	1EQT	3ES1	1GXL	3BM4
1G98	1XCA	2ZSK	1ZCH	3FFU	3SC3	3U04	2CIJ	1K2F	3K7Y
3FGY	3FC2	2ESS	2YX9	3DVH	2YHN	3LRQ	3TSM	2PFB	3GIO
3G9G	3EYY	1GL3	1FI4	3M1Z	3IJO	3LQS	1EX2	1JG0	2W21
2A8F	2XL9	3ZYC	2EO0	3SOY	1G57	3NK1	1PJ0	3ER7	4A3N
3QTP	2Z61	1RKT	2Q2R	2FB2	1EJE	3L8D	3NKH	3K79	2XLS
2D6Y	2PK7	2BX7	2FZF	4KC0	3LZW	4A22	3F4N	2JCS	1TWD
2ZVI	2GDZ	3TG9	3N38	3T2U	1Z9M	3PC6	1GPU	2WQF	3TLY
3N2M	1R7J	2PEY	4FBK	1P6J	1Z2I	1PRH	3H3M	2QTM	3K6K
3IO5	1A37	1AR0	2EGD	2RDP	1N1E	1Z72	2XPE	1RZ0	3OB6
3LT8	1UTX	3B0R	2ZUK	2Z10	3GAZ	3G7S	4JWQ	4HE4	2JAE
1UWL	2JG2	1LY1	1NR9	3LWD	1OTK	3E7M	3CNK	3OJE	1I3L
3FHO	1KZK	3RTY	3E9M	1OGZ	3PM6	2Q3B	2J6H	2I0J	2QCI
1MKH	1M0D	3NM6	2YCR	4IXO	1E9P	1FKK	3TZT	1K0C	2IXH
2PKF	4GEL	3VPM	1C1D	1TC2	3I1K	2Y5S	2YHE	3TN4	1VI9
2YN4	2F9H	1DLJ	1XS0	2WGE	1RNE	3VS8	4B28	1DJN	2PKE
1DIG	1L1R	4GMG	3VMK	4GLI	3RMW	2YHW	3PM9	3LYD	3L9F
3KC2	2OGY	4EHI	3OP1	3AW1	1BGW	1UMV	2F8H	1E3L	2X44
2WWU	3N16	1X77	3GDM	3BXS	2VKV	2HKZ	2PZR	1NJC	3HS3
4LFG	1VHX	3QWE	2GST	4E0V	3P8R	3P7I	2E83	3BW3	3R13
2BO3	4H7P	3SIY	1GC3	1EE2	1MZH	3Q2L	3MWM	2Q5H	2HU7
3L1M	1YDV	1CI4	3N8D	2X3O	4FUV	3CXX	2P4Q	2EV9	3E60
3M16	3LLS	3BKW	3P0T	1U67	2X1J	3QP8	3JYU	1N31	1YPO
1USO	3VYM	3JZ3	3HCP	1JIL	2Q2L	2Z28	4AT0	2WRA	2FT0
3N3Z	3LTV	2FJ2	4KSY	1KM1	3UBM	2DSD	2W8V	1JLV	1MJH
3Q5W	3AGX	2HPV	1SJI	2OYZ	3QOO	1OSY	1YTA	4DNN	2Z46
2X3V	1G5T	3AVQ	1AZ5	1N8T	2GVL	3I9K	3P1V	2E4Z	3F14
3EY7	3CQ6	2C2X	3FV6	3PL2	3OHU	3EDD	3FHX	3ITQ	3EWN
3R0Y	4LC6	4A6G	2GBU	4E4T	2NTO	1Q9U	2HOQ	3T7M	1VDC
2R52	1EXS	2HH6	3EY8	3QKC	2VXN	2HSB	2XGG	1I69	4FR2
3I38	1JA3	4EXN	3ZBV	3GZ7	1H6O	3NVA	2WAP	1XW8	2DY3
4AAM	1VQA	3E03	3ZS7	1A6P	2VNS	1GQA	3A74	1IBD	4J15
3AIG	3ES4	3C48	3TX8	3G0T	3I6U	2A3N	2JFQ	3NYJ	3E2D
3CHI	1E5M	4IOH	3ISH	3EEG	2WT9	1JUO	3BV0	1VS3	4HX6
3G7R	3AWJ	1XML	2R14	3ZLM	1WZ3	1EEQ	2Z6C	3GGU	2P82
3RJW	1V96	1CTE	2P08	4AFL	1NJJ	1S9J	1MK4	1SHJ	1GMP
4APY	1EN4	3RD7	3LY9	3UT8	3BNV	3LMZ	1SQE	3O1W	2ZSJ
4AN6	3NSL	2PWO	3GHJ	1VPA	1A8L	2WGL	3LHX	1B76	3ZZ4
1T09	3N2Y	3DM7	2OOJ	3QMV	1HNB	4DY1	2I7S	1VAF	1ZM7
3HWL	3F4W	1UCR	2W1R	3GTI	3ADO	2NO7	1QQ2	5GRT	3D0R
1G8S	3S2R	1USC	3QXB	3DAQ	4AJ3	1FBQ	1HDY	2C1L	3IBP
4IQI	3DMY	2JCG	4I8P	3FVQ	1RLU	4J9X	2JK1	4F5F	2CCG
3HAC	1R3R	1H8X	2NS9	1E5X	3M1U	2P6L	1ID4	3FFH	2IKS
2F1O	1JGT	1AZV	2J67	2HRO	2DI3	1CD8	2RHJ	1P1V	3IL7
2WM1	1PVD	1JOG	1GTO	2ZZ3	2DWO	3FU8	3GYD	1RFY	2ZB9
3Q0E	1O89	2H9V	1Y67	3HDO	2XCH	4ESW	1CNZ	3UHS	1IEF
1H2N	3M3O	2QTE	1T04	3EA0	1R5K	2D1H	3DPJ	2OFW	1E7R

3RZA	2WAH	2VSD	3KT0	1KTC	3R23	1AQY	1JWJ	2QTT	3ZZW
1VR0	3VQL	2E3T	3PWG	1V4V	3Q91	4BJR	3O4I	3ABI	3MAU
4AJF	4EQS	4JKZ	3QU1	2WYQ	2XLP	2F42	3F9F	1IAX	3HSR
1LGQ	4GMD	3B42	3NOQ	3KK6	2A87	1TPW	2OZH	6ICD	1R3W
3EUC	1K9P	3GUV	1RRG	1R6Y	1VG4	3K3U	2GRZ	3OOD	2PO3
2JJC	4EQR	1FC1	3AFC	4DZH	2J5V	1H8Y	2R9Z	3HP1	2A35
1Y42	3ABH	3DJG	3LWB	1KIJ	2FDD	2A2Z	3VWX	1KD0	4EXO
3VM9	1AWD	4EUN	1AJ5	2I4D	2R8Q	3DYD	1MD3	1B9H	3JTW
3UH7	2I7R	4H3C	2IPB	1OBP	1SEJ	1BYS	2AB2	3F47	1WU7
2I0X	1OJW	3S5D	3MZ1	1XJK	3LSN	3MR7	1UI7	3C7I	2ANH
2BKA	3EZ0	2Z26	3FQA	1JUJ	3VPN	1J3M	2R6Z	2O1X	1CZB
1YDY	3FH1	3MAK	3HRI	3Q1O	2NYG	2G9Z	2FGV	1YHA	3C3K
2Q7X	3F3H	1PFQ	1TXY	3AXA	2DY0	1QY4	3CT6	3TK0	1T6L
1YUX	3OHN	1RQL	4F1Y	1AX1	4EOG	1L6R	1HYO	1C7H	2DQW
1EMF	3O73	1FDV	4AOV	3CU0	2BK4	4DPO	4AQN	2OUX	1EG5
3G16	3MOL	3LLL	1QH3	1H3U	3R5G	3M0G	3CDC	1IEC	2QA5
3OKS	4DBD	3S23	4FX9	3FA5	1Y97	4FWL	4HES	3UR5	1UNA
4EQQ	3TJ8	1F6D	2QX0	3N4X	3GU1	4KN5	3M0F	1XRE	3LCE
1RKA	1W4N	1W07	4GUF	4AIB	1H4T	3EIB	3NUZ	1K66	2EMQ
2UXW	1K68	4FYR	2IWF	1KH9	1O50	4HZP	1R30	1XHY	3OJG
2W3Q	3BQT	3QQD	3RIM	2GC3	1R1T	1IYZ	1KCF	1N26	1ZGJ
2VGV	4GMK	4DGS	3HZU	2ZHO	2ABQ	2GYY	1CHM	2FVU	2E8C
3BOQ	3LUZ	1DUG	3HLX	1T33	3NYU	3HZR	3RD3	3HEM	2ZBB
2ACX	1VG7	3LL6	2Y08	1A4I	3NYM	4H2B	4JBB	2Z8H	4AXD
2IA1	2HJ0	3K21	1AFR	3S5Z	2WEA	3QNC	1P65	3CAM	3HWR
1UDC	2I1A	2CO5	3L8A	3D40	3HF5	2X0I	3GA1	1HKW	3HHF
3OTL	3TYX	1A4B	1OHQ	3CRU	1ZZQ	2C20	2Q2H	3T7Y	1R6T
1S5K	1IJL	1J1I	3PP9	2EL2	2Z0J	2WWO	1TVD	3I3W	3PJL
2W2F	1GC8	2GH1	2X5T	1UER	1BO4	3RJT	4BCA	3URB	1TKA
3LIS	3OXN	1M9R	3P9V	2JA9	2QO0	4K8W	3QT8	1WD9	3DBI
2QU7	1RV8	1GD7	4BC6	3C6M	4F82	1C7N	4F62	2HD0	3EDN
4E85	2HJH	1YQ7	2BZ4	3AEZ	3RB7	3U7J	2FFG	2GPU	1ASB
3JQR	3LF6	3NH4	3M8J	1DHT	2Z5F	2JAT	1TIS	1YLQ	4AWQ
2P5Q	1KBZ	2XVH	2RB7	3V7M	4AMT	2AB5	1CZ9	3GFL	4EPS
1YPQ	1G2Q	3S19	1WOV	2OCZ	4K8D	3IOY	3K9C	3SZJ	2QL1
3KM4	4JAT	2W7M	4IOT	3ED4	1YLM	3B1R	3DTJ	4FBL	4IR7
3O1X	2WUZ	2CW2	1X87	3HG9	2E11	3TFW	1AUC	2YCI	3APZ
1J55	3ULX	1Y23	1U3W	1BGQ	3G6O	2Z0W	2AA4	2WBG	2EWT
1QQJ	1U0U	2HUN	1J7N	3C0M	1EW3	3K9U	1JEZ	3M43	2CST
3CYF	3U21	2DAP	1ELY	3P61	1PS7	3SM3	3BRO	2E18	1GH7
1TSD	3NY5	2YGO	3SZA	2G3O	3NIV	3C4T	3RZP	2WBA	8PRK
3K4H	3UYU	1TLK	3LST	1R3Y	3OOP	2XK9	1BYL	1BO1	1SQI
1OQ4	3T9P	4AG7	2GUG	2YRI	3DL6	3F6A	1OKJ	3I6W	2YY5
3VL2	3ZQV	4INA	3OSC	2OGU	3TP0	3FJY	1D0V	1P5T	3EYW
3G15	4DQZ	1FIE	1NOX	3G8M	1MTC	2IG6	3AMF	4IPI	3NNL
1X8X	1DFX	3UAF	4AU1	1V8T	2NYS	2CDU	1GNE	1E97	2I7P
1VIA	3NR9	2E2P	2GFY	4A6B	2DOU	1WYX	2IJD	4AQP	1HW3
2Q24	2PPV	2YK5	4JHR	3KAE	2J6G	4DIO	3G14	1CW4	3F8G
3I0P	3TQK	1LKZ	1B3D	4H9T	3S91	3EKT	2Y5I	3CTZ	3S7Z
3H65	2OV9	3M2P	4ATY	3PBW	1UXM	4IC3	2OEM	1E9F	3O0L
2W2A	3QSB	2QNI	3N4P	3DXW	4E5S	2CFY	2B2C	3P60	2NV4
3EGY	3H36	3I6A	2HOX	1N2F	4E5Y	3RLZ	4F8D	2Q1U	1I21
3BYR	1KCM	1CI7	3DO5	3S2Q	4HAI	3QK7	2QQ9	4IO6	1IX7
2I5D	3GRN	1VIQ	4GT1	3FM5	3HFY	4IMP	2BTI	3T8T	4HL9
3EW2	1CHU	2O4T	2F7K	1G2W	3QMT	3OM0	3CP6	4J7K	2QHK
1UIS	2OLM	3E2S	2V0X	2D4W	2DRV	3D0J	2PIH	1USB	2D1C
2FL4	3KW2	1J3R	4E9K	2OAC	2O9C	3TNG	4JJP	1CU1	2RIU
3FMW	3LYH	3W5I	3QKX	1M75	4IKH	2OZ6	3ND1	3NQ5	4F2V
1TFZ	2GPY	3NUT	1JIE	1WW6	2ZOV	4EAE	3ROT	1AQE	3KAW
3NWX	3OWF	2CGA	4E43	3NHX	1W61	3CTV	2W40	1ORU	3O1Z

3IQM	4IA5	3H50	3C26	2EKL	1A27	2O4L	1R3V	1WKQ	2Q2J
2E7F	1SQS	3VTS	1FN9	3PAJ	4BD7	2PFI	1YEM	2AEX	3TNZ
1QP8	2DV2	2CUN	2XJ7	4F0Y	2I6L	2FHQ	1JQX	3SSX	2OK7
3AAT	2CW3	1BKJ	1ULK	4I19	3LRD	3LND	1UQ4	3F7S	2X64
1G7W	3BVQ	3CEH	1QCO	3S4J	1OKI	1M0S	3PUH	1E6U	1SJ2
4AE1	4A03	3RKY	3QNI	3EPW	2F96	3KDK	1M2B	1S4K	3UT4
4DMB	1C8U	1Z5N	3CPO	3B7H	3PU7	3H4O	2EEP	4HI7	3G46
1EPA	1U0W	1MFT	3CDY	4A35	2HXW	3UYI	2O2Z	1KL2	1AHE
1QKS	2XEU	4ECL	2IYO	1HN4	1A0G	3EUT	2RGI	3LJB	2CAQ
2EMU	3LZC	2DXT	3FPK	1BG5	2X5G	3KO6	1YUZ	1QFE	4J7P
4B0T	2DR1	2RGY	2D2D	3BEU	3KU7	3B5I	4K2H	2AW5	3CL1
2AL2	3P13	1HJ3	3V95	2OY9	2J68	3N0Y	2PW9	2YCD	4J5P
3HO7	2NO4	2OOD	4HA0	4BC9	2OWQ	4E8O	3GW2	3PD4	4F5J
1QHM	3I17	2ZZC	1YYV	3FO5	3MCZ	3RAO	2NLV	3G5G	2VAF
1DHR	2XOT	4GR9	3GZL	2AUM	4EAX	1GU7	3GK7	3MT1	4J8Q
3QJ9	3BN1	3SJG	2B1G	3LR5	1FRO	2YIL	1N9E	4BMG	3ENT
3DSH	1X84	3K5T	3INM	1A9Z	3ZZ3	3GDK	2J0N	3TVI	4EY5
3BQY	2ZO6	4DO2	3R3X	1X13	2B4H	1N5J	1CGO	2OZE	1WUU
1O22	2JK3	2CNB	2X3J	3OGA	2C2A	2I8E	3GW3	1V8W	1JOE
3GUR	2OHO	2NVK	3KO8	3MAS	2ZV3	1XVI	3O6Z	4HR1	1BHT
3PNO	3RVA	2HJD	3CBY	3F2V	1KZJ	1YCO	4BC7	3PZJ	2DWU
1E2J	3HTM	1K9U	2BHX	2QMM	1Y11	3EFK	4HQM	1SW7	1LNS
1AJS	1R0E	3SD2	2WFC	4GXD	2QE9	3MPQ	1VSK	3FCN	2Q7H
1C8T	6GSY	3UHQ	1JLY	3GLA	2E7I	4G86	2I3O	4DSB	2GZ6
3LSC	3KSR	2W3H	3A0C	2OHV	1WYD	3JSJ	3N0T	1TU8	3U1W
2VFA	2IF5	4DKL	2NLO	3LCR	3W54	2E7U	4GIW	4HN1	3PUB
1EAJ	3UHB	3Q01	2QDO	3OQ6	2HBX	3T2L	1UOD	2ZP1	1QAF
1Z9P	2OBR	2PAM	3UFF	2R82	4JXQ	3R19	3T0W	2X3L	3HMZ
2OPY	3ZZ5	3KUM	3K30	1Q98	2AR9	1QZ9	3ORG	1JAY	3LAS
4KQA	2NX8	2F8F	1CR6	3KOL	3GEE	3FBG	3DL2	3KGB	3DS5
3LXU	3APM	3M5B	1V5X	4GWH	2QEC	2WUA	3FHS	2DD9	3TR3
1YJY	3E6M	1L8P	3GX9	2PSN	1UOU	2P2S	1TCW	1T3J	2GE8
1IC2	3BDB	4FLA	3UST	3EP1	1JQK	4EZB	2CXN	3BJ8	2GZ4
3TDJ	1W02	3P1M	2GZW	1MJK	3RHF	2G04	4FS9	1P3W	1M32
3PY2	1KVU	4HFV	1OX8	2PD0	2XFV	1NFP	2FA2	1PSU	3UH3
2OMA	4DBH	4F4E	4IDN	3A16	2D52	3OZA	3KHN	2GB3	1UR9
1QKM	3SY6	2RK3	2OWY	2YSK	1F9F	1GSF	3Q6A	2HTI	2P11
2HXT	3G1Z	1TD9	3SM9	1EA5	3LWG	1XI8	1XPY	2Z1Q	3P0K
1AW1	1T62	2PO7	2GPC	2X6G	2XL6	2G4C	3A20	2PK3	1ELQ
1S12	2VPH	3N62	3N0S	4E1J	3JSL	3NRA	4EU8	1BG6	2W9I
2XPK	3ING	2D1F	2YX8	3UOW	1U3Q	1YSO	1YV9	3LVY	3DCJ
3D72	3Q58	2O6D	3HT2	1JDF	3PWA	4EQX	3PNM	3CRN	3E7P
3JQQ	1IZI	1YYR	2G9Y	3FJM	1PXB	1K9T	3P0F	2HKV	3UWQ
2PPT	2E2V	1S06	3Q0X	1OY1	3HCO	3NTV	3SXY	2WK6	3SYL
3QBO	2R0X	1ICW	2VC3	4KGI	3OYR	3P2O	4A3T	3D26	3NOX
2JBA	3FJ7	2CVI	4E3T	3HK4	2ZIF	1GMG	2O2E	3AHH	2YZK
3G1H	1ZZG	2PSQ	1G2U	2FH9	2Y52	3KGZ	2PZM	2RAH	3U1N
2XSV	2O20	3ULC	2VSV	1LCL	3N7H	1WLW	2TS1	4BBB	1YFD
2WZ1	1RZL	1K0J	3VX4	1QMG	1QB7	3DP7	1TMI	3MC6	1EYV
3OA4	2G8A	1USL	2XKJ	1BAJ	1Q0Q	3ZXB	1OFZ	1TSY	3GBY
3BG9	3HQ9	1G5X	1OD2	1A5V	3GFX	1S20	3BI2	2OPO	2SPC
4HKP	4GW6	1TVL	4F4H	4FX0	1ZLJ	3TLZ	1IBA	2YAB	1DR8
1OHY	2XF1	1VQE	3RQS	3ISQ	2YI7	1VQI	3R9P	3E7D	2QHC
3ZRW	2WQP	1I5E	2OEJ	1MSC	3L7X	1E71	3LGG	2H8E	3AST
3UW3	1ZX9	2QD0	1SEV	3DYC	3ZIB	1FUX	1XAC	2PQM	1WV8
3KH8	3MBK	4EUM	3N3W	1NIP	2PLA	3V4K	4E51	1B8K	2YY4
4FOM	4DPD	1RTR	3ZW5	3Q5J	4G85	1KP0	2CCY	3P8L	1GPJ
2VH3	1YDK	3F3K	3S18	4IR8	2EBB	3LTM	1HQO	3O61	2B7L
2BZS	4JUU	4F8T	1OGL	2IZ0	3UB9	3TER	3LLW	3B96	2EG6
3DKH	2W5W	4GFC	1Z8H	4JL9	1XDL	3PRA	1ZSO	3P9I	1VP4

3VJH	1JGS	4EX5	2RC8	3TV9	2Y0F	1JB2	4F0R	3EWM	3LNC
3M46	3DTO	2Z8A	3ACS	3NZQ	3IX6	3IJM	3FT5	3LFV	1MWQ
2KI5	4GRX	2WDW	1OHS	3KXL	1CJ2	1NCG	1GC9	3HFU	3GX0
3ZW8	3MFX	1L8D	1W4L	4G6H	3NFW	2VJM	1OFN	1JWK	3G79
3MWK	3EA9	1I0L	3TKK	1Z9N	3K9I	3AY8	3FIR	2J5B	2C3T
3QNE	2ED4	4FIV	1VLK	1WPO	2Q6F	2CBN	1VQF	3HUP	3U0F
2X3F	3M4P	2J62	2OYE	3R2Q	3SQM	2ZXI	2AXW	3FKJ	3M47
3D7E	1O5T	1RDN	3NUI	3KLW	4AIR	2XZ7	3FX3	2QYO	1SFJ
2J9B	1N9W	3OV8	1TEE	3EAO	1KVR	1XHN	1PXA	3LB5	3OSK
3LHT	3CJP	1KMJ	1D5N	4ESE	3L6B	3TLO	1G8T	2QB2	3GGQ
2R01	1DV7	1UW1	3TFT	2R43	2OBB	1FXR	1YPP	2ZZW	3M9Y
1XI3	3HCY	3PQB	1IX8	1Y7T	4AXV	2VCJ	1DDR	2ZG6	1IAG
1QYC	1VGA	3LHF	1EV8	2EI5	1YLN	3FOV	4EDF	1OUW	2CXK
3F3E	3FET	4DJB	1CSM	2Q0B	3MBI	4DIL	3H6N	2ECP	3VDN
2IP2	1Y4L	2PZD	2NQK	1COZ	4JG9	3BL3	3OHR	1EZ2	2F07
3DV8	3TQT	3PGY	3H01	3QRV	3KVI	3FHW	3EEX	3VAY	2RCV
3NQW	4FP7	2IK9	1AOR	3I5C	2BWP	2QTB	3OXX	2WHD	2CYY
3OYT	1F6L	2DDK	3IC9	2FTO	2QIW	1OAT	3F5D	4BES	1XRH
2GIY	3TA6	1QIS	3M41	3SZR	2CH2	3E5V	3HHP	4G3K	1SUI
2PIM	2QJ8	3UGD	2H1T	3F69	2AVS	3RAC	1ZXE	3PI6	3KSG
2ZAS	1KHH	3CCY	2BNL	3SB9	1IDM	2W3P	1BDR	3IL8	2WVT
2BMC	1ZCP	1O5H	2VFG	3O2F	4B8B	1IL0	2IBD	1JPH	4FGL
1YNU	2JCD	1OAJ	1PCF	2YL0	2P9D	3NOS	3OP7	3IK7	3IPF
3MCQ	4J3C	2J1O	3K6T	3IBM	3QNL	3BIL	1B48	2JAO	2OPT
4ECB	2P1Z	1YQG	3EX3	3GZ4	3HQ7	3MES	1ESN	1HQS	1ZSY
4A7P	1Y37	3B4U	2VP0	2R53	3BS3	3G8Z	3KOR	2IYP	1BGJ
3C18	2R9A	2G39	3QPL	1BWS	3MGU	2ZUT	4EUC	2J4Y	4ICH
3DZB	3TN3	3L1L	3FVX	4B4C	2YY2	3NX3	4ED9	2XRL	2X8J
1W00	4I3M	1BDL	1ILS	3UAR	1V9Y	2I02	1XKF	2B1Y	1BV9
4IN2	3TJ1	1SW6	11AS	1OW2	1ZX1	4BI3	2IK1	1SXC	2P2W
2WKD	3EVT	4J40	1QNI	1DXX	3PXO	2V6O	2JL1	2XXC	4B8W
2OO2	1LLF	1XQU	1X9E	2VPQ	3L1F	2F8J	4BDL	2OND	4H08
3M8V	1J6X	3LKK	4HRV	1PV2	3TWQ	3H1S	3IL9	3CB0	2R83
2I7A	1KOB	1BOT	2J24	1QM5	1HF8	4EJ8	3JZ0	2VXB	3H2B
1FF4	3M92	1EXQ	3K53	4EM3	3F4A	2YSW	3G3Z	1U9O	2HCM
2D4U	1XBW	1CI1	4JE1	3MX1	3ZMD	4BC0	2ODM	3HHX	4L6W
2XJ3	1E58	1J9A	3VTF	1HW4	3MAP	1E9B	2ZWM	2BTM	1CJ4
2AL1	3NSI	3F5H	2X1K	3DMA	3TH1	4EFZ	2QC7	1LL2	4E4C
3R2T	3NBU	4FX8	1JV1	3VC3	1TXN	3E7L	1VGY	2NWU	4GQF
2VL1	3GE2	2FMB	3JTJ	1RGB	3NUK	2VGY	3ZWD	3P5Y	2IRW
2HU8	1CJ1	3LQE	3AXZ	3FFE	2ZM9	3CET	1OSI	3B3A	4FNH
1A25	1QB3	3E5D	2HXU	2CSU	2FCK	3G23	4FD3	2HP5	1PRG
4A1F	2D4C	2XRM	3D6K	2YQ3	1SU7	1OBI	3HVV	2CMJ	2F4L
3DD9	2UUV	1EZ1	1I88	4DQC	4KKM	1LWH	2FXG	2XSR	4G2B
2P3C	3P8K	3GFM	2HUT	3T2W	3GLX	3IAL	3B53	3KEO	3BXU
1C9U	1R6D	2HZ5	2GU9	4L8J	1BDM	1WC8	3F6W	1B8Z	3IE3
3QWM	1DLM	4AO7	1E9I	1LKC	3HHH	3EM4	4B56	1ZRM	3EUS
2A0K	2NV9	2WV7	2O3O	3U1V	1AZB	4IP2	1OFY	4DPH	3OE6
1V2D	3Q3Q	3MJK	3ZWO	4H7N	2PAR	1VZ0	4GPO	4GCI	3PBU
3HLU	2ZAK	2ATI	3IDA	3P2N	2HYX	3NQ7	2GJG	2OME	2PS1
1TYA	1A8O	3CY0	1B9I	1IUX	1JZL	2FNI	1MJF	3DUW	1LVL
2OU3	3E56	3TJ7	2VFE	2OBC	3GDW	2Y4O	3NBR	4AYT	3MJO
1TJD	2VHD	4BBC	3DLO	2WCE	1KMM	1NYH	3KVR	3L7O	3N2S
2P9H	3V8D	1ZP3	1F1E	1AV8	3N0L	1EKU	3EHI	1JB4	1DQR
3O63	3RLV	2EWS	1AR2	1ELZ	1U8E	1VGM	2QQZ	3CAZ	3B4W
1VK6	3KB0	2D63	2QJF	2XLE	3HF1	3HV0	3R0I	2E2C	1EYM
1PQP	1T10	3BQX	3QLD	3LHV	3PA8	2RCZ	1HCI	3EO6	3GAG
1O6I	3ZRG	4AJR	2JLD	2FYX	1Y7A	2O96	3CQ4	3ROB	2EJW
2J96	3TWO	2XV5	3LJQ	2AUO	2D3M	2R6V	3PSS	2XOL	1FNO
1VRB	3CSV	4AF5	4EGT	3E4Q	2FKN	2PK8	2EXU	2EVV	4KWA

3ALA	1JU9	1VHI	3G7E	1TIM	2QSQ	3ECF	2C9S	2O7T	3JU8
2XBG	3A9W	3LEH	2LJR	2XHF	2Y4D	1LSO	3HFK	1FLG	3QIT
1NHJ	1C02	3ACO	3SYV	1C0P	3L7H	3G1P	3RJS	3LR0	4B7I
2AXQ	2IXK	2J70	1PTZ	3UNA	2TMK	1I3C	1IBF	3F2U	2D1G
2B3V	3ZCH	3TZN	2G7G	3UPM	2IJL	2O7G	3MSE	3IML	3UHA
3S9F	1KKR	2ERQ	3VLN	3RV1	4BE3	2WMM	3LQ3	3R8E	3SAJ
3TH6	6FIV	1CM5	3GRZ	3NTE	3RH8	1UI8	2W1V	2G84	1HW1
2OJP	4AYW	3SO9	1ZBQ	3ANU	3U9L	2IFX	3A9L	1FP1	1PT8
1VL4	3DNP	3VAB	4AC0	1ODB	1U5J	3BRU	3ZN4	3BBJ	2BHJ
4FF7	4IAG	1GD9	2ALV	3U15	2VFH	3AVE	3PIU	3U1D	2R5W
1JMH	4D8F	3GMG	4HMA	3QLT	3G1W	3KSV	3PI7	2I5P	3POL
3V7P	1T3E	2P8U	2O38	1ZVA	2HNK	9RUB	2HY1	3S6F	3N2Q
1XNG	3E3H	3NYG	1VG6	4FRY	2NN3	1GYO	3A36	3TR8	1QAC
3NME	3HHD	3QZE	3Q1K	2PEK	3GLV	3OY4	2A4E	2O7M	2IMZ
2Q03	2CKI	4EJO	3ZZD	3P5N	1BWB	2PJS	3ID2	1BWW	3R09
3MDK	3SOP	3UB2	2A6A	3MBF	3HEB	1QZR	1IVY	3DTT	2QTR
2XRF	1YYA	1ZEO	3II4	3U2G	3TN1	2FFR	2QJD	2R6I	2QJW
1P43	2R6O	3CEW	4KF9	3Q90	3C0K	1VFH	3ZBW	1Q2W	4AQI
3O72	2IOC	3BW4	1P2P	3D8X	1DPG	5HBI	1S7I	5EAA	2G40
2HUZ	2IWH	3T9O	3RBL	1IS6	3I5T	3T69	3D62	2QTA	1WL0
2HUH	1XD7	3NK6	4AY0	2V6K	3KBQ	2C3B	1IB5	2WP7	3N1S
2HA8	3NDC	2CZ2	1TLV	3F4L	4BDG	2NZ7	3NEI	3GTV	3DVT
3O4N	3F6D	1GQ1	3U0O	4GQW	3OFG	4IW7	3OQU	1XSE	2R26
2XGA	3P2Q	3MJY	1EM6	3MPH	3H2Z	2V40	2C08	4AXS	2J7T
1PYB	3ES2	3QP5	2PZP	3FSL	3TBA	2OQI	1R22	4EP4	3SDO
3V69	3TBF	2XIJ	4E4J	1E2R	1KMZ	3UQH	3TZ6	1HUU	1VGQ
2W5X	3QBJ	1U8S	3UQN	3NFC	2B18	3FJT	3VGS	3ROU	2RSP
3LVE	2HYO	4AYA	3OO2	3GZI	3T35	3FMY	2ARV	3PDK	3K6Z
3TTP	2NO2	3EN0	3LHC	4BL4	3O0M	2Y92	3EIU	3O10	2WB6
3O1N	3NWR	2OWV	3B02	1TYT	3ERM	4FCU	3TN5	1S4D	3HR7
1T70	1NOK	3TRO	2YG4	2V03	3O2K	2DDM	3LRJ	1NCE	2DKH
3KW3	4I7H	2ZUC	1LUL	1VJQ	1TSI	2A61	1B8U	3EJO	3L20
2QYA	3T5T	1URU	2R47	3N2B	3LWC	2PO5	2AVO	2B4G	3G3S
3CBU	3TLB	3B8V	1IQX	3RLC	4GFL	4F98	1PBB	4D9O	4ISD
3TD7	4DF9	3VK5	1IGQ	4G12	2C82	1EVL	3PG8	2A96	3JTF
1WA4	1GJQ	2X3B	1NKD	3FPJ	3GFA	1EYE	3GWR	3E5U	2CU3
2BIE	3R0G	1IEG	1UWK	3LWJ	1QFH	2C5A	1Q05	1B6U	1ETO
3G8O	1ASS	1M44	4A16	3PFE	3S2W	3IF0	3IQY	4DVH	3RGL
1ON2	3F8R	3R3Z	1K0Z	1AN8	3PHA	3F72	3UCA	3QTH	3VRC
2Z25	4JQP	4GIC	3M1G	3TYA	3QM3	3F4K	2PY3	2HOY	1MY6
3T1I	1AIU	1YB7	3O4Y	1TBP	4I3L	1ONS	3DXI	2Y6B	1I12
2VJP	3BLL	3FF0	3U83	3TQU	4AW3	2Q30	3EL6	1J1C	1EPZ
2BM4	2WJ9	3GV3	3KII	1FOF	2EWP	3H78	2EFR	2P3Y	3O4Q
2III	1DBQ	3UAK	3N4W	3RYK	1YAI	2XVI	3Q7E	3R0A	4MV2
3O7B	3NJO	3AAC	3AXS	3GYW	2WUI	3EA4	3FXH	2ANS	4AB5
1MTB	1GUK	2QXS	2ZZ5	3PF6	2GIU	3RX6	3AHJ	1R9D	1P6K
2G9I	4HG0	2WZT	3RY7	4GCM	3EUA	1WWK	1P9O	1H99	1MNA
1X1V	4A5N	2WJX	2NZT	1KV5	3BBL	1V03	3NJ2	3ER6	3RQK
2XWL	2O4C	3HEJ	2COJ	1WN2	3VRH	3JZ6	2BPE	3AGA	2PEX
3KKC	4E1E	1F6Y	1FUN	3CGH	1YHB	4FUM	2XGZ	1B6J	3TV7
2EPJ	2OWK	2GYO	3AO4	2XR7	1F12	2OA2	3HN2	3FMS	2OQM
1E1T	2NRB	3B33	3T43	4DEM	1FDS	3A6R	3ON4	4DQ2	3VPR
2HYP	1PL2	2ZB6	1H1M	1CMB	4IV9	2Y84	3DMS	2VVX	1V53
1SMK	3RKT	3ON3	1HTI	1BTM	2PAQ	3JVN	1I1D	1WUE	2BN2
2B5A	3QEN	4FLM	1QSJ	2V5S	2QM7	1BP0	1ZKP	3MWC	3FDK
1VI0	3V3E	4H9V	3JX6	1O64	2PHI	3TZB	1ASU	4HWU	3FYA
4JGO	3H5W	3IMI	3OCA	1HDZ	3GFJ	3LI0	2JJF	1QC9	3GZC
2NCD	2PH5	2EH5	1H72	1OB9	2OKO	3PS7	4G6X	1NIU	2YZT
4BEP	3BPJ	3U71	2PPQ	1S66	4J6F	3K2Q	3QDK	4BDO	1IZ3
1VCV	2AQX	3TQC	3BIX	2WTX	2PGV	2DC0	2I51	4EQH	1MD4

3P7J	3MQ4	2YCF	2GQR	2QOP	2WUJ	3E5H	3E0Y	1OBK	1U3I
1ZA5	4GR1	1S09	3CNI	1GNM	1GT8	3IHK	2H8I	3C8E	2J8R
3EX9	1SW3	3BT3	2J3N	1YY7	2YEA	2P12	3QT5	1R43	1IQC
3LVS	1NRG	2ANI	1OTW	2O1B	3G1X	4HKU	3OF5	2YR1	1TP2
2AN2	1XK7	2RA6	1B63	2E5D	2WFH	2X0F	4E0B	3LMO	2GUU
3Q37	3QPH	1BP6	3LS9	1VLC	4GXG	4H3O	2WLO	1G8L	1EF0
3NOV	3TKU	2AMJ	3HJP	2WPO	1T3G	2WVN	3CZ8	3EN2	2I9I
3I6X	1Y5H	2V4N	3RD6	3UER	2BSM	4ALG	1NS5	2BYJ	2NX4
3K1E	3P8T	1JIQ	1IBB	1IZY	1M9N	2ZJ3	1M76	2PCA	1RPA
1AUO	2XH0	1BJW	4INJ	2G89	2TRT	2JKY	2PH4	2ASF	3KTY
3GRA	1N99	1GZW	4ACI	1SEP	3D82	1W1U	3GFF	1ZCZ	1XD8
2YAZ	3LDT	2X2S	3L43	2XZ2	1JWN	1EZI	3QP6	2WQH	4L0N
3H8U	3I4J	3BPX	3OCX	3KHY	3RUY	2FGC	1XVS	3VA8	4JNQ
2NRO	2H2C	2UW1	2IVY	1TYD	2VCT	3O8Q	3NI6	2BIH	4ECC
3LOW	3N3R	2EGW	1VE9	1UBV	3RF6	1CML	2X06	1JYF	3ACT
2DU9	4H87	2XLD	4EQW	3RT6	4ART	3UA3	3U2M	2ON7	3IGR
1FA8	1T1S	2X1Z	3Q29	4ATM	4DMG	9GAC	2CH9	3CY6	2ZVT
3G7Q	3LAG	1FC4	2G87	3ZJL	3B1D	2WES	4E8B	3EN8	1I2L
3P96	1JL0	1AQ6	4J7J	2XGT	3N29	1YLK	3HL4	2AX4	3CS2
1A43	4GAE	3DEV	2DBO	1WJG	2I4L	2WEP	1NP6	2PSR	1HSZ
2Z0A	4DBC	3K6H	2C5S	3O55	1KM6	3CNU	1GNW	4ECH	1INR
2W43	3NZP	2QIS	4G41	2Q79	2Z2W	3HAD	1A7G	3A65	1J4A
4DBA	3N6V	4AU5	3RJ9	2FV2	4AO9	3DSP	1VME	3JYQ	2VRN
4F0C	1JDE	1XKO	1OLZ	1YIG	1J33	1JLH	2IK2	3PFC	3QYC
4ADD	2J7J	1VSG	1JYE	2Z3Z	1Y2O	2WYO	2X0Q	3L18	3BF4
3EGQ	3MK2	4AYZ	2P3A	1VMI	3CJE	1XXU	4GHJ	1YCF	2GUH
3HEK	2OWU	2E10	3GJC	3C5U	4EAQ	3D4I	2GSD	4GR2	4FUK
2GYQ	2B4D	2O74	1PGU	3RQP	3PLT	2QM9	3NMH	2QM4	4GWB
2ZOY	3JWU	3EDK	3DAX	1RBA	2VG2	2JAA	1TUH	2P5S	2D65
3MEX	3V4Z	1DIK	3E0L	3EGO	3BOS	2IHF	4FMK	1I0R	4H9U
1IJY	3UV1	3BSF	2JFU	3IPI	3HJV	4G3V	4AV6	3PSV	2ZM7
3W5W	3BNJ	1Y25	2OKU	1HUR	3STQ	4HZ2	3K8A	3EBU	2X4I
2JBW	1UES	1VLJ	3EXQ	2VUX	2H2U	3EMM	2P3D	1BHY	3AHG
3FCM	2RDZ	3FJV	2AV8	4AGI	3IJQ	2HN1	2REY	2XUA	1EBL
3KR1	1MZF	1G7X	1EZG	1SB9	2ITB	2OD6	3T8K	2RK9	3MP4
2JCX	3CES	2V5L	2EX2	4DHL	1X6M	3JTX	3HL0	2WZO	2P8J
4E2G	4EH1	4KGF	1ZBR	4B54	4A0G	1ZXY	3IKH	1R8J	4FEA
4HCX	1EOG	2W27	3KMJ	1MSV	3ZBY	3OLQ	4IJA	3BAP	3K40
3H1N	2QG7	2G1U	3PWR	4J2F	2JGT	2DD7	2QIB	1U3J	1ZPR
1PDV	4JXN	3OP3	4HFG	4HAM	3LV1	1OY5	4FDV	4HZN	3BRI
3TJR	2DC4	2B5W	3A4N	3I3H	1AG9	4CSM	2G6J	3JR2	4B1A
2ZLY	2H2K	3E39	1HAV	1Z45	1EQW	3S8K	2NAD	3TJX	1ALI
1YXO	3L8U	3TOL	3NAQ	1S2P	1YZX	3UB4	1E19	2F48	3CXP
1U6S	3VBE	1M9T	4EWP	1TMH	3V9L	4JWP	3MGM	1JBG	4ERA
3CVO	1ZEJ	2D13	2EB0	2AQS	4FDB	4DNA	2VFW	2I3C	1KSI
1IXM	1LHP	1EO6	2J0W	2D2X	1PDH	1Z5A	3AU0	3JTH	2BHS
1K8U	2ZNJ	1R0K	4AP5	3CP3	4J75	2YYV	2ZZ1	1RL8	1W7N
1V8Y	2GEN	4E0E	3IL5	3ITF	1UUF	1WQ9	1ZCY	3U1B	3TBM
3KNW	3L5Z	1I07	3OXP	3LQ7	1V2Z	4G6C	4AKK	3MTV	3B1T
3PJB	2XH4	3UHW	2O03	4H9Y	2Q3P	3PTY	3RGR	3TY7	1HLP
1SZ2	3M6J	2HNG	4L8N	3ZWP	2PBX	1WC9	3NC4	2XGX	3P01
2CAR	2DLB	3CSX	3BEM	1JMI	2IJJ	1YW1	3ADT	1AAQ	3IAO
2VPA	3QEO	4F28	3GVV	3B7L	1SOX	2Y78	3E48	3FEG	3BDG
1V5H	1V5B	4FJ6	3OCY	2CJR	3NIB	4HW8	3ZV8	3ICX	2ZU8
3OKW	3KDI	3G3K	3PKO	1VQD	2GMS	3F8D	3DR7	2Q1X	3I2W
1USM	2YXE	5MDH	1PTA	2HQM	3VML	2YMA	2FB1	4G2E	2RC3
1IQB	2F3M	1V2X	3EFE	3PX2	4DFH	3IFE	3E9K	2OD0	3EQR
1BPJ	1BW9	4JNN	3OK5	1PQW	2PV7	2QMO	1UKK	3DUP	2Y4F
2GSQ	3OQA	3QTT	2I79	1PV9	3KD6	1A9Y	4H3Z	2Q3A	3IGF
1NXH	3ID3	2C5J	2JIS	3UY4	2OHC	3F0A	3FF1	2DW6	3U7I

1U7W	2CG3	3RH2	3MQD	4GEB	1WY2	2JGD	2C49	4JRL	2O6X
1AF5	2Y0Q	3EGC	4B0N	2OOK	3GMD	4LVE	2I4V	1XUB	4AF2
3FYN	4AQB	2I3D	3E17	2A72	4HQV	3MCW	1VD6	1F5V	1VE2
3EXC	1GO8	2PWL	3OZ7	4EUT	1I7F	3G8Y	1HYV	3AEX	3O31
1WPP	1MD0	3MMH	3M3C	2PJQ	1YGT	2ZB2	3EDE	3UM6	2E6X
1KM4	2OCP	3IPT	1T92	3R3H	3TC1	2Y0O	5RUB	3FFY	2FA0
2POB	4DN6	3AIA	3HA2	1ITZ	3ZH0	3H1O	1DQW	3BMZ	2C6X
4FTX	3VVZ	3ET5	2CL3	4E3Q	3AO1	2HS2	4F5Y	3E97	2Q1W
2HKN	3GGO	3B6O	1H21	2X2N	1VQU	2ENX	3L9Y	4B7C	1X7U
3E8J	1A0F	2RKK	1GRP	3LTE	4GSA	2ALX	2WCW	3TSN	3JXE
1M1F	3EJK	3ZPG	4FQ9	3OWO	2TDX	3KXP	1G5Z	1QOU	4FVB
2IF9	2ANJ	3REM	1V3E	3PF3	2PY8	4F95	1JYA	2IXE	3FS3
2UV7	3DR6	2GW1	3FLJ	3DVF	1EX0	2YDX	3CZA	2IWE	1U4P
4GYM	4H89	2RIO	1VJO	3FJ5	1YSD	4DQB	2H9B	1XTO	1GER
3GW0	2GQD	3F43	2IMK	3L08	2G8L	1REG	3EM3	4B74	3W00
4K7G	2JBX	3KDW	2WOZ	3EL4	2P3W	2GOP	2D4V	3Q62	1ZL9
1WL1	3QMR	3N4K	3D34	4ADN	1QI9	1JR8	3T5M	2I7D	3A52
2Q3M	1J0J	2X01	3CJL	3D3K	4ETS	2ITG	4EM6	1T82	2J98
4IAQ	2D0J	3IN8	3FN6	3QYF	2IHO	2FHI	2VG1	3BQ3	3FJ1
3E0K	3A60	2XEF	2YHY	3FHM	1WWM	1V05	4EGU	2HZP	4AQL
1GWJ	2XDN	2VR7	3B9I	2Y8V	3E4U	2PB4	1F1A	4GVM	1S3H
1VM8	22GS	2FE1	3CY1	3RH1	3SBR	1SCQ	3HKX	3KB4	2GS9
1HUX	1CL0	1O4W	1IPD	2F6S	4HN3	3DTF	3NR1	3U6V	3HFT
3MGD	4DLL	3A1I	3H0K	3QUA	2PYN	3IQX	3D6G	1XEF	2WKG
2PFL	3I12	4FZS	2IHY	1ETV	4K2X	1WKV	1GDH	1ULZ	3D3N
3RPJ	4F8Y	3OHI	9GAF	1G4X	1MG5	3G12	1X8Y	3INR	2J7Z
1XE6	3KZP	2ZZ2	1HL4	3I7F	2XT4	1GKH	2VC7	3LSS	2VJY
3L67	3IWZ	4AVR	1XF1	2AQ6	3UOE	1HRK	1MQE	3FTB	2WNX
4DB8	3UF2	1QE0	1I8B	3LE5	1TRE	1PP2	1NA8	3ZN3	3BA1
1W6Y	1YVO	3HDT	2H5G	1HZV	2OQL	2DKN	2A4M	2O57	1ZZR
1NW1	3C97	1VLR	2GAU	2FIC	2W41	1JPY	2GV8	2V2C	1Q2X
3QHA	3P41	1DQP	2PMF	1F1C	2CA1	2VSG	1ZGS	3P1J	1MGV
3PID	3RKS	1QOR	3TNL	3UE9	3D5N	1ZK9	2H0U	2J1D	3O0H
3V48	4IQ0	4HAC	1ETX	3VLK	3ZY7	1DOV	3GOA	3BJE	3VZ1
2G5C	3C1Q	1ALK	3VGJ	1U7D	1VE1	5FIT	3BXO	4HB7	3NSX
4GNV	3ZVI	1T3I	3FVM	3RUC	2HUV	2ZM2	2FPN	3NEU	3IT3
2WKI	1YUD	4AIG	3IQ0	3GBR	2IPH	4J7U	4H49	3G80	3IFU
3QO5	2Q8H	2XP1	3EB0	3TKJ	2WV0	2VNV	2IXF	3HA1	2A2A
3KEX	2VRO	4BDR	1DTS	2GBB	1MGO	4G8S	3B5K	1U36	2CYC
2IQQ	1OXH	2VJL	3TY3	2NR4	3LZS	1QLL	1PM7	1GYX	3MOS
3GBV	3HJ3	1CZ3	5JDW	1CZJ	3TYP	1IG0	3LSJ	3PUI	1U2W
3Q7L	1U87	3IBS	2YF0	2BII	3G7J	2W2G	3NQ6	2GC9	2OU5
2XT2	3HMU	3OTR	2Z02	3F0N	1E5D	3HDC	1ILK	3N7U	3D8B
2BHT	2YGU	1VSJ	3QTC	2XCO	3UN0	3U8Z	2R6M	3BPV	1N8K
3NTL	3HKV	2XSQ	2QQQ	3E5N	1I83	3G7D	2IOH	2INB	1QIT
3R77	2OMK	3MVG	1X1Q	1TIY	3LYX	3Q80	3NQK	2QAI	3FPE
3ZXQ	2P4G	2REK	2X3E	3EDW	1AMK	1US6	2VYA	3EHC	3T8Q
3O6G	1EVG	1XFD	2HHZ	3QK9	3B7P	2HQL	3TE7	3NX4	3DO8
1B49	4EXJ	1Z7D	1UAJ	2UUQ	2C3S	1N19	2ZNY	4FBM	2QUM
3TNB	1R7H	4IZ9	2ZVY	2CCA	3ZDU	2XGU	3Q9C	2GEL	4A3Q
3JPU	3ONT	4GSS	1DDZ	3BID	2ZM8	1VQH	3LR4	2IML	4HCE
4AK6	4AJ6	2A2J	3RIC	3CT9	2VPK	3EBT	1VF1	1G1A	1KA8
2VAJ	3PLN	2AS0	1A7W	4HYL	3QII	2OKI	4E4R	1Y8G	3BXW
1U0K	3CEQ	3GR5	2RA5	2PNH	4EC7	2SHK	2CC3	4AK5	1XRK
2QL8	2E67	2A0U	2GUC	1Z1J	1B98	3UV0	2C9Q	1O5W	3K67
3FY6	3ED7	4H3U	1BGF	1WNF	2YJG	1FP5	3LG0	3DZ8	3C3D
2Z0O	2XXX	3O1C	2R6U	3EH7	2E9Y	1LGP	1UV5	3N04	2DGN
2Y7A	3OLJ	1CG1	1VSM	2BB3	1UB7	3A2L	3CNG	2ITM	3BNY
3UL5	3NBS	3W0D	1SQU	3QI3	1EE9	3OK8	3T81	1UTU	4F5H
4EFV	2HIQ	3IVC	1I45	1Y7H	1LPF	3NB6	2IQT	1VJ2	1S07

3RBU	3RMH	1KM0	1ZVU	2Z24	1YQZ	4JAQ	1XV2	3FRS	2BGC
3CIS	2D1L	1P4R	4EIN	2DXQ	2A99	3BOA	3I0T	3QM2	3LZQ
1PN9	3VZ0	1UAQ	4DN7	1A4O	1TFG	2QCO	4A01	2VOM	3HDJ
2X5H	2PUZ	4E71	1TW9	2GUX	1ESC	1Z05	4DFE	2P64	3HKF
3L65	3QE6	3O74	3DWC	1E9C	1JMF	1MP9	1ZW5	3N8W	2AZU
3TN6	3OD1	2O0T	2V3M	3BB9	2VUO	3KB7	1ITU	1KNW	2B2K
3N1T	2PQV	3NON	1TD2	2XJ6	3PX3	1AHP	4FUO	2VAX	3FZ5
1EI1	1Y4U	3AC9	3LNR	1P6N	1PCZ	3K32	3ZXT	3ESM	3BDF
1YKW	3HS5	1F4Q	2Y74	3F7C	4FLB	3TRF	1M0W	2ZCN	3G48
1AXG	1L4K	2HXO	3B9T	2QE4	1H1C	2G6W	1NF9	1CQE	1UWW
2QNR	4G9B	3LQN	3BHO	3T9Y	3TDM	4KFN	3UPY	2YIJ	3KOM
1PM2	4DUP	1EV7	3DNS	2PTF	4FTP	3OZL	2QYB	3KKB	3I3N
1ZVN	2P55	1MO0	3V8R	3LHR	1NVJ	2HB2	3KBO	2VET	3A1E
1VPL	3OMS	2HYT	3M8E	1F13	1EN5	1YCL	2O4M	3QBW	4HSZ
1NSJ	1FON	1QPQ	3OHT	4GA3	3NAV	3I32	2WIQ	2AWP	1UYR
4H02	1K2U	4EP5	1MSS	3W08	3LA3	3STT	3K7T	2XXT	2YC0
1F75	2ZL5	3DR5	2O3A	4INI	2CY5	3OJO	2I9E	2DIK	3UDX
1ZKA	2ZKJ	2I9U	1PFC	1HTT	2DG2	2FMY	4H9X	3N2O	3EPV
3RH7	1V70	2PFC	3IWJ	1O12	1XQA	1QJH	5BJ3	4AE7	2A8N
2QTQ	3KVH	3G4O	1ZES	3KZH	2IME	3FKD	4F5E	1Z5O	2RHE
1SD6	3GDR	3F9S	2LIG	1T5O	4DIK	2PS7	2XBQ	1S0A	2VFZ
3LI7	4GEW	2QCQ	3NEK	2YKY	3QST	3KXO	1OHB	2D5Y	3RR3
3ITJ	4HUZ	1UX5	4ILK	1TO0	1WV4	3BTU	4JA8	3NRQ	1BUO
3DH1	3T0X	2YBO	3WRP	4K8T	3GAS	3F84	2X3G	1TZB	2OF7
3C9D	1Z8L	3CAI	3KP3	3QQS	3V1X	2ORD	3OXO	2V4M	2Y40
1LCU	1YER	2H3B	3ZY2	2DGB	3DB7	2HXV	3ZT3	2AQL	2E0T
3GTT	3G3J	1RRM	3MIL	3VE9	3IXO	1GQL	3C4J	2D2G	3UHV
1D8H	3KS2	1WA9	1TVE	2W57	1VH5	2PRX	2A9C	2YL3	3ZYL
2Q3T	2OGE	1AD3	2Q4O	4FDL	1YYT	1IE3	3Q18	1U42	1AN9
3A5P	2HYJ	1FB7	3TFH	2PZB	2ZY9	3OD0	3JSS	1L7E	3Q12
4FE7	3H7F	4AEA	2B60	1YKD	2G5T	2AV6	1ZH8	2QM3	1ZZS
1X0J	2EL7	2HKU	1TLU	3JU2	1RGG	3R60	2PTY	2PZ9	3GH0
4K2W	1Z6Z	1RFB	1KZA	3ENP	4E5K	4ING	4IPL	2AF3	4H65
4GYH	4A7Z	3KLX	1WZI	3BCX	3JUZ	2X3C	3VOM	1FCJ	3SJ3
4AEC	3KYZ	3RQ1	1HVS	2YBQ	2DFI	1U5W	1EV9	3FKT	4EJR
1V1O	3KKE	2PSS	3LMD	4L1J	2EUC	3NCV	1QQH	1XQK	1T8P
1DL5	1YRP	4G5A	3M1E	3IS6	3K4S	2R42	4DUN	3UT1	2ZEW
3EUF	3SK1	1PXT	2YXH	3F9T	3DI1	3E9Q	2F5G	4GAP	3E5T
3FCD	2OGI	1EK3	1Z83	3FPD	3R8W	2IV0	2BES	3JRZ	1BF3
4H1Y	2B8M	1WWZ	2R5E	1ZKZ	2J8Q	1KYQ	2D2A	2BSH	4EF5
1N1A	2GD5	3LZA	2AZJ	3VFB	2VEA	3SG6	2VIB	3TGN	3GL5
3QBD	1O9Q	1WGT	3GDO	1E1E	2NUL	3EI5	4DKK	3AAK	3BV4
1PGT	3HA4	3QU2	4HBP	4A1Z	3F3X	2CVW	3BUT	3LHU	3FGW
4A6U	1SFP	2FXJ	3CE1	2ZUG	3N8B	3TQX	2DQ0	3DMC	2RKF
4H5B	1OJ9	2XGD	4H0P	4BR1	3QJ7	2Q2I	3ZUY	1YGA	3FH3
3HJO	1ZBS	1IQ6	3MPN	3TDG	3GFD	2AML	1D2R	1P4A	1ARH
2VL3	2BTU	4E77	2NX9	1E77	1BIS	2C59	1X74	1BO7	2FRE
1WQ4	3LYG	3K2N	2A67	1AE3	2CZG	1N5X	3K6E	4GMH	2FDS
3U4G	3MI7	3F7E	1WAL	2CJW	2Y1Z	1BYU	4AMG	3IIC	3HID
3DF8	2WTN	3LA0	2XLO	1NWI	1BKG	2E25	2HGX	2QDT	3NQF
4JOS	3R6J	1FP2	1W9C	3NI7	2QO3	3VBB	1PIZ	1HFS	3ONX
3AUF	3CY4	3V6N	3EAW	1DPR	3CNX	1EN6	3PU9	2BJI	4F9I
3DN7	3IW3	3Q31	2JJE	1T4C	3KTL	4EMY	2B69	1AY4	4F44
3JX8	2P4P	3K0Z	1U0M	2GS7	1JF9	4JIX	4GXO	3VOL	3HL6
3U1U	4JQ8	1CTU	1AZW	4I9G	3MPB	3T38	2WGG	3N3Q	2VD9
4APS	4B0Z	4GGK	1SG9	3D00	2AVD	2WGI	1N3L	4F0B	1MH9
2C7T	2WEU	1USR	3FGV	3LPL	2G5G	1FVF	3CLQ	2F5D	3HMK
2QRZ	3M5P	3U7S	2GVI	3E2M	3HBQ	3SB6	1TLB	4AMI	1N1B
1Q2H	3TR6	3GG9	2ZYA	3IS2	1REI	2JJH	2ZXL	4DQN	3JUW
2BDR	2CUK	2R6F	3BJ6	1SGM	3CUK	3R4Y	3NPK	4JVO	2XTY

2Z7J	1ARI	2DCM	3IEF	2ZL7	2A8I	2I10	1M6J	2PAK	2C76
1UZ3	3KJI	2YVN	1OUN	3N6W	1QPA	3NO5	3IA7	1YEA	1OYJ
3ALE	1PYD	1NT0	1FVP	2PIG	4G79	2HMP	3QH2	1NNW	1E0R
2VB0	1YB2	1ESR	1MJV	2QLZ	3CWR	2EKA	3V7I	3JY6	2FSW
2Z2R	3B7C	1ZMB	3L2B	1VFR	3SXW	4FAC	3E18	2RDY	4JRH
3Q0W	1DKS	2XQ2	3Q9B	2QPT	3SON	3ENU	4AC8	3JSB	3EHE
3UB3	1VG3	1QAK	2OMO	1H91	2ARZ	2IJK	3EPN	1Z91	3Q7R
1PDZ	2OD4	2QKP	1XCP	3DCA	2XSA	3FK4	3DTN	2PB6	1VQV
4IMX	3ON6	3QAO	4DO5	2X52	1T3C	4BFX	2IK7	2ZGT	3ASB
3Q0K	2IY2	1HZJ	1XPQ	3H4L	1EWJ	1SZ3	4DQH	4DVD	4EIA
3UHR	17GS	2GYR	1FF5	3OLT	1EV4	4B4W	4FE4	3AI7	1RCW
4E7O	2RGH	2V8D	2PWJ	2QAK	2EEN	2CAN	1NK1	1DJC	1WRP
2W8M	2F3G	2HOW	3S52	2POK	1ALU	3GXO	1WR8	1ANI	1OIV
3ZXI	4FWR	2XH7	1K26	2ZOF	4F80	3MWH	1CJ3	3EJ8	2EPK
1BWP	3QDO	3AQI	1HUL	2CBF	2V0O	3FQQ	1OBQ	1V84	2B48
3DEE	2VPR	3D7Q	3N2I	3L8J	2OKF	3FKQ	1G3W	1JDN	2IU5
2R3X	2JJ7	4EZF	3HJ9	1CDO	1RMD	2A8X	4AW2	3NAU	3B09
2E4R	2ZTK	3AUA	2BH9	3OCO	3P04	2YRF	3KE3	4JZY	4E0A
1HT9	3LXY	3FD4	1DD8	1V3V	2DQ3	2W8X	2AUN	2VMX	1VKH
3U9I	3EXA	2GE7	3JV0	4G9M	2D5M	1JOP	2WTE	2CMG	3VC5
1DDX	1K3E	3C24	2FB0	1TEL	1R15	3HYB	3QEZ	2FB9	2JKE
1V0D	3MDO	4DJ2	2QAE	3C3P	3ZT5	3U2T	1ZH1	2DJL	3CGB
3PNK	1ODY	2VOC	2QIQ	3U4A	2JKZ	3HVP	1D6H	4IFF	1TU1
1I13	3I83	2HS1	4H7L	2NWV	3COL	2DJZ	2ZZ8	1M2D	1EHI
1H0C	1ZG3	4A7X	3LOC	2Q6Z	2QLD	1GEU	2JC6	3PSW	3A32
4E08	2QUD	1K6Z	1FS9	1INJ	2PLJ	4DKC	1ZK8	1BEO	3KV1
2CL8	3HZB	1F4D	3U4F	2QNL	1QX3	1T9M	2DDC	1WL3	3GWN
1TIK	1D6S	2V8V	1PM3	1JVD	3DQP	3M3E	1UUR	3I4T	1VJL
1NRX	2ODF	4L7W	2B2O	3LTB	2Y3M	3SY5	1ASF	2W90	3MJQ
2H0H	2JLH	3V3X	1IOL	2WGK	3DR2	2YK7	3AH7	2E80	2Y39
1YSL	2X9M	1HJR	1RR2	3KSP	3OWA	1HSI	3I6M	2DHT	4FZR
3LY7	3AGC	3SLB	3CJY	3FLC	1J6W	1ERN	3G13	3TW4	1KPB
3NQN	1PA0	3A3X	1XE7	1RCQ	1NWP	2ZDO	3AHC	2BYW	3UMC
2V7S	2X2M	3FY3	3EBN	4IC2	3F6C	3DYQ	3HHV	3OKX	3OU6
1AFW	4ES3	3EZ3	1PEU	2FC1	3KWK	2OKV	2Y4N	3TJL	2QUI
2HG4	3L5L	1ZC6	3KKY	2QQB	1LMK	1V7R	4J4C	1GY5	2PVP
4GI7	1R28	2RKB	3QAE	3ENK	3A9I	2HZB	3JX9	2H09	1S99
2Z7C	1K6C	3HSY	4KMR	1P4K	3L19	1H94	1GRO	3QBM	1M1M
1JFL	1B62	3KAY	4FIS	1LOL	3OII	3AHI	3K2H	3D7L	2H2Q
4B04	4E28	3T1S	2HRZ	1W01	3CC8	4HHE	3N65	1B74	3QJO
1L3P	1JR9	2WFD	4AXL	3UM9	1CG4	3URH	3H1P	2GK9	3U5V
2QCW	3VQE	3BKX	1M99	3RMJ	4IYJ	3KZB	3SSA	3C64	2YCK
1U7N	3O27	3FX7	3BJA	2HF9	1CNO	1ML6	2XB5	3BF5	2G7L
2W8W	3NYS	1Y4J	3N9W	3OZW	4GPI	3KRK	3KPC	1G8Q	1VM7
1XWG	3EZ1	1YQX	1BYK	2VCY	2BDQ	2HQ9	1W8I	4DZA	3CM3
3NVU	4AYN	1WPY	1TUV	2F1L	3PC0	1PDO	1K04	3QXX	2WKY
2QYZ	4IST	3NQA	1ZHQ	3HGQ	3F7L	4K7Z	4E70	3E2C	4HC4
1CMV	4AVM	3ILX	2BSJ	3DAG	2VJO	2W31	4JJI	1N7Z	1GZ6
2RBB	2XZ9	3UGS	3LUS	3FWN	2RAI	4KNA	2OAD	3DY6	3I42
2XL8	2P04	2G36	3GEH	3H2Q	4DNG	4AWY	2Q9R	4GH6	1SH9
2F7S	3TL9	3KBF	4GDH	4F2C	3O9O	1CGZ	2G76	3EGT	2D2R
1RTS	1SD7	1XAO	3N4S	3O6D	3G7P	3LC8	3HI0	2OQR	1XTY
3FIW	2WQW	3LII	2E46	2EHP	2Q58	1K51	3DJ6	2Z9A	3DDS
3FK9	3IGH	1V7W	3BWN	1Y0B	3LV9	1K3S	3I05	3GIU	2FZE
1NE8	1G0Z	3DT3	1SMA	2QMX	3EEQ	2R27	2QHL	1VI2	1SMS
2G3P	3VK6	3FRQ	1H0X	3ECD	4CTS	2OBN	1NU3	2F83	1WZ7
3TJT	3FM2	2EH3	3NNR	3CU3	1DJ2	3ZJJ	4JC5	2ACA	3OP2
2EFJ	3NXS	2IMN	2O1Z	2WCR	2QGH	4A8Z	3KF0	1DNL	1KBN
4DO3	3INQ	3DI4	3C2S	2Q8R	2IEA	4FUW	2XCC	2XRE	4J29
3BDE	2ELD	3S6T	3SN9	1NXU	2OP9	1SAW	2WZJ	1WWP	2OL5

1HDR	2BHM	3GB6	3D0Z	2P5U	3VS9	3KDA	3T8E	3GW4	1BV7
2FN1	2YPR	3C34	2F8D	2PL7	2EV4	2W2B	3H2D	2FTZ	1QKU
2EFV	4D8T	2A1S	3EZS	2RH7	2IPR	2ZF3	1VZ8	1JRC	3ISX
2V27	1Z4P	1ULH	1O9N	2GFP	3O2I	4I1L	1TE2	4A5Z	4I66
1ZOR	3LFL	4AG0	2OGS	4D9M	2B61	2V5G	4DIR	2OWL	1GXK
1NF0	3D5F	1VJB	3MP3	3DJA	3DNH	2BP1	2Y0P	3H05	3L3B
1GCJ	1I0I	3M1Y	3HCM	1EWR	2II1	3C3W	2BC0	4EQ1	3CTP
2ZCW	3HJX	3IKL	1J1A	3BM5	3E5Y	2QRR	2PS6	3OCQ	4GW9
2ZOZ	1R4F	3SEY	3DNX	3EO3	1PGJ	2ELB	3BEO	1NX2	1PN4
1Y0H	1GNN	1IPI	1NDA	1LRL	3HTR	3P0R	3BDR	2ZGQ	1Z85
2FYI	1BZO	4ACS	3FGE	3S46	1VGZ	1IUB	2XV7	2Y4R	1GKD
3SPX	2GAN	1PMT	2J9H	3DPM	3CXY	2FXV	4B6I	2BHO	3LTX
1H7X	2EO4	2P1F	1QU4	2EW2	1VGU	3ICA	3KXD	1ETY	3H0N
2A88	2WWR	5CSM	1KWS	1AIL	2A6C	3TGV	4EUU	1BIZ	1U6L
1H4V	2IN5	4GAK	2JL4	3PPU	2Q82	2XWX	1DJL	3G7I	3PJZ
3PJ0	1IE0	1E98	2P8G	2WQD	3T5D	1GUP	2DVY	1I08	2QFR
3QMO	1O0W	1EK5	1DK8	1O9E	2A11	5ENL	1VG2	2NZO	3NQG
3DWI	4E1P	2I8D	3RU7	2X0D	3KBX	2H2B	2E1H	3Q8W	1R8G
1GO7	1LL0	3ZYE	3ZFB	2NYI	2HI1	3BM1	1IAZ	4FMR	1WGJ
3CT8	3W4Y	1P6O	2I46	1TT4	2R1V	1SZ7	2B67	1VH9	3C8X
3JS3	3HJG	2NYR	3CO8	3FCR	3VPZ	1QWD	1OVD	3BRQ	3FRG
1IED	3PM8	4GZE	3F3Z	2HUL	2Z0F	1R0S	2CKO	2PFW	1VH6
1Z68	1RIB	3BXF	1VHG	2NRC	2QYG	1XZ8	4BA0	3NH8	3TG3
3VH1	3H5Q	2GFN	3SZ3	1PFO	3OIT	1THF	4EIV	1RVE	1C0N
3HTV	2FBH	2WV1	3P3A	2R38	1Y0G	5CTS	1W5F	3E26	3U55
1BT4	1U07	2P35	3NE8	4IUJ	3ZJH	2NOV	2FA5	2IIM	3E2Y
2UV6	1BL5	1VIU	3B9G	2C0Z	2FBK	3DEU	2DG6	3I1J	3F6T
1PVM	4FHT	3IH3	2BQQ	2NSN	3EO8	1VZV	1NWW	2FRH	3EZF
1DMM	2WFN	3I64	3TQV	2ECF	2IIR	4L59	1F36	1QMA	1ZRS
3FGG	3HAJ	3MHX	1U4M	1SW0	2D7V	3HB8	1G3Y	3U5W	1N1C
4AV7	1P6B	2FVZ	2ELE	3O52	1E7D	2ZO7	3BL4	2JLJ	3PPL
1EVJ	2QUQ	1CBK	2WVO	1JHC	1KVQ	4HK4	1WLH	1TUW	4H8A
3GLZ	3QZZ	4FH7	3LX7	1AY2	2FJT	2X5R	2YK4	2P9M	3OI8
3F2I	2OB3	1TPV	2WSM	1P9B	3TZE	1X7N	2VUH	2J7O	3K41
1Z3A	2XXU	1H1Z	2WY0	2PS4	1HYU	3B5G	2HAH	2ZE3	3BBR
2E0K	1UZR	1V25	2H3D	4FGW	3GLB	4FEI	1BIH	2CB0	3BB5
4E74	3G17	3OMT	2W8T	1TW7	2WU8	1KHZ	1LJ9	3PNU	3BJD
3K8J	1G85	1LRH	3TG0	2YKF	2ABJ	3GPV	2GFW	3PFH	3LM6
2P25	4DGT	3F41	3DJW	2PHN	1KJI	1VC1	3NP7	4GE0	1B0X
2OM6	1U0E	1DPM	2FOR	1G8E	1BBU	3EVV	3H3H	2AKO	3GZQ
3NQO	1ZVF	1DWI	1GT5	3VJZ	2E68	3TZA	1O4U	3G6I	3F8B
3TE8	2OAM	1QLV	1UI6	3LWS	3LIO	1LYN	3IF2	1H65	3BBY
2AEJ	1IDE	3FGH	2Z29	3LBY	1KLL	5P2P	3F1B	3TCS	3QH9
2DQR	1BMD	3HY8	3M3S	3ETC	2H63	1EA2	1NQD	2PYT	3B3L
4DN2	1POC	3AKJ	1TOK	3G8K	2YWV	3NM2	4B3A	2B0A	3V1P
2C4M	1EI5	1ANJ	1E6B	1ZYB	3K94	3FBT	3GUW	2P05	3VJM
2Z6E	3DUV	3RFC	3ERS	3KSU	3MMS	2GHV	3AMZ	2Y6A	1U7X
2YC3	4GD0	3T4K	1SU5	2PBR	3EIA	3TY5	3COS	1T5B	2E54
3I0F	1A1X	2BC1	1LTE	3CAX	2QR4	2GXF	1X56	2J8Z	2C54
3VX3	3LZU	2I0C	3EFA	1CBI	2DVM	2FCO	4DVJ	2FDO	3SJF
1AZ4	2JC3	1KHJ	2GVJ	3RQ3	1PZW	4JIM	3F9Q	2EF8	2CG6
3HX8	2QHD	3G3H	3DV3	1G50	2GBT	3TKA	2ZGP	1JRB	2ZGK
2J80	1TOE	2AUA	3PG6	4E5N	3HBG	1OP8	3CS3	1JFG	1A6I
1N57	1NZI	3GWQ	2ZGN	1HZY	3ESF	2G69	2J3M	1KI3	2OO0
3KHO	3LU1	1AZT	2RIL	1PGP	2W4E	4EOH	1Y7Y	1I1H	3ILW
2BEO	3LMB	4EWC	2E85	3UEH	1BM9	1AU1	2J6B	2R4Y	3E15
6CSC	2IIK	3GB3	2OYC	1KU5	1W6P	1QIR	2NVN	1PSC	1F3H
2ZOD	1UXL	4FE2	3G3G	3AG5	1OAN	4HFS	4GCX	3GM5	2PHM
3FN4	3RNR	4JDP	3O2J	3L12	3PR3	2ESR	2W8Z	1YOO	3Q8N
3HOI	2Q64	1AD4	3UHD	3FK7	2OKJ	3C9T	3CWC	4HAF	3LKI

1PXC	3FMB	3F0C	1IMC	4IVF	4GFA	1GJU	4GI5	3M6D	3D0F
3RF7	2TDD	3IL6	3NMP	4EBF	2PX7	3A5Q	1DNA	1Q41	3TTE
1SWV	3R2V	3AN1	3K69	1RC5	4K6A	3MDL	4HZ4	2ZGS	4HXT
4ALO	4JXK	2DX8	4HEX	3GRD	4AS3	3Q2O	3PVQ	1IYH	1VPB
1NPB	1LOM	3NOO	2A4X	3DZC	1NJ8	1LBV	1R11	2OI2	4A5O
1VGV	6GSW	4IG6	2CH5	1Y94	3GOR	1DYU	2V32	1ZB8	1MI8
2ISK	1FEE	3UCW	3C0F	4AGH	3HQ6	1TFE	1DFO	1IOM	2DUM
1OAA	1F0Y	2I8B	1ZJC	1JVS	3GUF	2PCK	1CX2	3L0H	4JWT
2XUR	3GTF	1BDQ	3NSM	3LNP	4FW8	2O1V	1FOC	4ET0	2CYA
3BBD	1TWU	4J10	2D0I	2FXE	4GYT	4DYL	1SHL	2DCJ	1JDW
3NFU	3U95	1CSR	2WJD	3OMV	2DPL	4J41	3CEX	4JOQ	3GS3
1UB2	2IDL	3OY2	2P92	1EMD	4H8E	4FJU	2DQL	1DJQ	3GYQ
1FDW	1PZM	2WOW	3NPF	3NM8	3DSR	2RBC	2Y2J	3H6G	3GX1
3UPT	3V05	2DH5	3MIO	3MAV	3KYG	3QJ5	3BCV	3FF6	2AVV
1B8A	1I89	3U3P	2VKW	1GSU	3UF5	1UDV	1RKV	1E7L	3LD6
3VSY	1YJG	3W3J	4E3W	3HPX	3EWX	1O4T	1JGM	2IK4	2FQ4
2EJC	2Q8T	3ZYX	1TCX	3IVR	2I2O	2X99	2FTR	2G0I	1ICI
2OB5	3IP3	1QDB	3DQR	1KQC	3EIK	2HDB	4GE3	1WUF	2QZG
3TAL	3FBS	3US3	3AQN	3KXQ	1X0V	2FGU	1MLY	2NQR	3U80
3BNK	1AJD	2EIX	3A1N	1PUC	3P5Z	1GTU	2G8X	1Q09	3TY9
2Z1T	3EC9	4AOU	1OQU	1VHM	3EDP	1UMU	3HT5	2DM9	3L46
1ES9	3QS2	1ZKG	1UB9	3OE9	1JQ6	2BZ3	2IA9	4GP6	2YGK
1BKP	2NMY	19HC	1TYC	2QGS	1II7	2CZ5	2FLE	3TCM	1QG3
3MND	1AY0	3TLC	3IR9	2ZZ4	3HNE	1O81	3D7A	1WLK	3L1E
1CZE	1SP8	2R1I	2GFF	2R5N	3HQ5	1PL1	3KAJ	3R6M	3VH2
1Z7U	1AB4	3CP7	3UCG	1GMV	2OWM	1MKK	3TNO	3ZCO	3F9G
2AUD	1VKI	1SQ7	2DC5	3G8E	1JRQ	2WND	2I45	1OCM	1CSG
1F2D	3KRS	3E6E	3EOF	1DOB	2Q8O	2GUP	1TSW	3C8M	3I2G
3VOS	1AIB	4E5R	3TBO	3GTX	4BEZ	3T7E	3IUV	2VGZ	3P6B
2FC2	2IWY	2Y21	3DYG	1J85	1QJG	2PQ7	1FLM	2Y2Z	2EWN
2O27	3QOZ	1JXA	3IL3	2IX4	7AAT	1Y44	2R6K	2EVR	2E6K
2Z6D	3JRT	2F5T	1GRT	1GVI	2NRF	3EHJ	4HMZ	4JVW	3D4J
1QXX	3QGU	3G3I	3LHK	1URB	3BM7	2PEI	2EG5	1GXF	1VZD
4GDP	4EJQ	2FBN	1K4Z	2QFV	1ZXN	4EVU	3DLD	2O95	1SOS
4IR0	3EKQ	3LG2	1P9N	1JPI	1BGN	2AUW	4INB	3RHG	1CB4
1BW0	2WI8	3G27	4IDQ	3KG8	1TSN	1C1L	2IMO	3E7S	1K75
4F35	1SE8	4IXM	1PS6	3R41	1Z4E	1FF9	3BZH	3ANM	1F18
3VOE	2UY0	4BKP	2X76	3LV6	1BZL	4G2U	2NSI	1DAA	2FEX
3CTA	3NYL	2ONT	3BFM	3KG0	2DSB	1AAM	2VDJ	1UBX	3FL7
1F89	3CGU	3GE6	2HDK	3NUO	4EDZ	2VRB	3LLM	2V4O	3NET
2Y1H	3QFM	1G4V	1PXX	3UWW	2YWA	4DEC	4AAO	3FKH	1XMA
3N6C	4IM3	1XP9	1RKY	3CB2	4FIN	3A8S	2GWG	1SNN	1TB4
1CKJ	3UCD	3QW3	3D8U	1OGB	3MAH	3UHK	2ZXF	4EBK	1O69
1E8A	1ZRL	4F07	4BI6	2HGZ	3NJB	3GFG	2QMA	3H9Z	3LRY
4ESF	2FIU	3Q7D	1X0A	3KLO	4DLK	3RSY	3LPQ	2Y6U	4IRY
3SR3	3GR3	1NRI	3GH6	1VA0	1A05	3VPO	2YLI	1Q7G	4HWX
2A8M	1HTB	2P0U	2A62	1LSJ	1NMQ	2OOE	1B78	1W5X	3B8T
1JPK	4ESY	1CX8	1PYP	3M4I	3CAN	2Q13	1Q6U	1HWR	2QIF
2GHR	2CO3	1HE7	3NGX	3NA2	2Q50	3HDG	2E5W	2Z99	1JHD
2HO5	3CSI	3FRL	2IFA	3NPI	3I27	3AIO	2VXY	1Q47	3RMG
3L24	2QJ2	3KK4	2EGH	1WYZ	1R9C	3BMP	1D9G	3NUA	3UA8
4AHN	3HKP	1SFT	2HZT	1KIC	4J5U	3EO2	3NNT	2GHY	2ZQ0
3I7D	1IQ8	3KGX	2JK6	2QS1	1GPR	4A27	1DR0	4ILR	2P0V
3EDV	2E1N	1HQA	4FX6	4EXR	3KXS	2Z0N	3NJ0	1Q50	1QV7
3LQ2	1ISR	3T0K	2ISY	2OFX	4G1T	3B1B	3MI2	1JQV	3QFV
1OHO	2FA1	3LYP	2YVS	4DO0	3GZR	3O8S	3E8O	3BXX	3TR2
3NOR	3NQD	4E6W	2CWE	3DGR	3MCP	1SUR	1AZ3	3M7W	3LV5
1DBS	3V76	3Q20	2PEJ	3LM9	3L44	3L6U	1WXI	1CP2	2Z0V
4EMW	3QFL	3JZD	3IN1	3S8I	2ASR	3PUM	2IEK	2PNJ	1EGA
1S9A	2NS7								

Table 9. List of PDB IDs of all monomers (Test-set 1)

2AP3	1I2T	1MN1	3TNE	4I8M	2XUU	3L6T	3E7W	3E66	2Y1M
2R5V	4AKQ	2H7R	4GR5	2B1M	1DKQ	2YLK	3IGZ	2HF3	2WOY
3FND	3BFX	3DDY	3QKP	4IJD	4F5V	4GRI	1VYI	1GFK	209L
3P2D	2HZ9	3FCI	4FD4	3KHZ	4HN9	1M7X	3KFV	228L	1HVC
4HS1	2PC9	1EZF	2A5J	1MWP	3UT3	4AZI	1N7R	1RLR	2YV8
2YFP	2FK9	1GV7	1PQD	3BLM	3HIQ	2HD9	3SKS	1ZJA	1M8R
2PSH	2J7U	4HVS	2VK2	1SI5	2YRX	2JCK	3L5A	3W4S	3FSS
3SEE	4GHN	3OBE	3V16	3FFX	2PLT	2VDT	3KRG	2DH2	3GR0
2BIM	2NXB	1P36	1O13	1PQK	3VMA	3VUP	1WSF	3I8O	2HF2
1BSA	3GFO	1H9N	4GZU	1QGQ	3FFA	1TRP	1IHG	3CUC	1SJ8
4G1B	3TJM	3HW5	1BXE	1D03	3HX1	1W9S	2WXF	2GCJ	3VXJ
3PAM	1XKS	2WBC	3S7Q	2CYJ	1OKQ	3PST	4AOH	2XLL	3UYC
1P90	2V1F	2PVB	2XVV	3UQV	1GBG	2OPE	2QZ5	1L2H	3OFS
1I4G	4D8H	4K2R	1XQD	3ELW	3NKV	3SLT	2EIF	3C37	2P86
4EEW	2EFG	4EPV	1YUO	3C10	4KY7	1OBR	1YQB	4HT2	2I9C
2CAK	4G1J	2B90	2Q0Z	1H4E	3CNH	1LHY	2OEO	1KGU	2C9A
2HQZ	2BXR	1XZJ	3UV5	3RM3	3CU4	3SV6	2OIQ	2I53	1T32
4H4D	4DWF	1GOK	1PF3	3PZ0	1B56	3KEP	2BUP	2IAV	4EZE
3U9J	3ETW	3GK5	1N41	2E1B	1MEO	3TEJ	1FYA	2ZP2	3DBG
1UOW	3NYV	3G5D	2VVK	1JKA	2WJH	2OYP	1O7Q	3IKW	1Y9R
4G8C	1DGK	2ZPD	4GYI	2UVG	2Z6V	3OM2	2OQA	4ICA	3K1U
2V4U	2IL5	2NRR	2ELF	3BNB	4FNE	2O52	1MN2	3QS3	2BS7

1LYJ	1SM4	3EFR	1GEQ	3QNM	3UZJ	2QPM	3TFD	3F4R	1UIF
1EUT	1T27	1AKT	2XWH	3PPS	3GHP	1O5U	3ERH	2XRB	3VIS
2WGX	175L	2OTT	3NMW	3SOI	1H0B	3T7L	1L72	1FR7	2YDL
3M6B	1N1F	2A6Y	2WCP	3DAJ	1K3I	3GFC	1O0R	3QSD	1EPU
3SC7	3I6C	2J44	2OCS	1JQU	3COH	1ZV4	3HBK	4DJA	1QW0
1EE6	4HY4	3FZI	1L38	1RPM	1PNE	2XOV	2PG6	205L	2AHE
1QQN	1QHW	2XMF	4BGP	2Y8B	4ETR	3K7L	3LJP	1FY1	1PZ1
3S98	1ROA	1IJT	3ZFP	1FMJ	3BS2	1CY2	1G7S	3AA3	1UOK
1C4O	1CTY	1FLW	3QP9	1SHM	1H14	1QWO	1A7S	3S7B	3GDZ
2FYG	1F82	3F6P	1KF3	2O2T	1EQP	3MNJ	3EBX	4AFP	2WV2
4EQ9	4IBX	1RFJ	1F9H	2A2K	3RXW	2IBN	2X49	4K8J	107L
3BT5	2GZQ	1AVS	1SYB	1VG1	1AUN	1XG8	180L	4DOT	3A4G
1XCR	3UEN	2PLC	4EC3	1DY6	1DPE	4A71	3H1F	1AMX	2Q4U
2FSQ	3HRE	1FSL	1U24	4B2F	3EBK	1PAQ	1QTF	1B6E	2BOQ
4H3W	3DVW	1ZCN	1JA1	1D9Z	3GMV	3TP7	1MJ0	3ZLY	1RGY
1L08	2VH1	3R0M	1YPR	3ACX	2HAZ	1AC5	2HCT	1VYU	4G22
4AII	3VIU	1RAJ	2V5N	2IJE	3MW8	3R27	3NGF	3HQC	3K1D
1L0I	2ANW	1IIZ	2QFQ	2W5A	4DQ0	3PAE	2QSB	3ABB	4G4I
1FHF	3CQV	2R0S	1IR7	4GIG	3UNG	1AEC	1GRJ	3IIX	2XSK
2XXL	1UAS	2YV1	2JHQ	1WER	3B72	3T3Q	3JUI	1ZJK	4JCJ
3K8U	4F1V	4F0I	3TQS	1QUD	4EER	2J1L	1MC2	3KCM	1V07
3OBS	2FN9	1T8H	4HHO	2C95	1HQZ	3RFS	2XMZ	3LH9	2BQJ
3NB8	3POY	3UEK	1XZZ	3FMU	1LZ5	3TRK	3DMQ	3IVY	1HN0
1BWL	2JG0	1W28	1JSX	2H1N	4H6Y	2ZOQ	1Y80	1SYG	3QJY
1GTT	1X46	1PRY	1ZJ9	3OBR	3JYS	2OH4	4IL7	2ETD	3AKY
2NSG	3BFF	4G39	3ET9	3JPY	3SOR	3SLA	4GLM	1TN4	3PQU
1KUF	1VBJ	2F08	4FBH	2AU3	3B0X	4BC3	1CCT	1XYZ	1F9P
3MJX	1P3N	3T0Q	4ETK	1ZSQ	3ZYG	3QFI	3OO3	1THG	1M7U
1RRH	3U25	3DI5	3W2P	1HFZ	3CEU	3KSX	2V4X	1NGA	1MZA
3UD1	2QY1	2W56	1BVA	2PBO	1QWY	2AHN	2E0I	4GH8	3VAC
4DOI	1W5E	1W94	3MC8	4EWE	3MXZ	3C5V	1L06	2FU2	4E22
1E7O	3UXD	3ZT9	1IQ9	1VLB	1GF4	3RIX	1DBH	3N0W	3KR6
2WW5	3GMS	1M6T	1UKZ	1YR2	2YE0	1JND	2YZY	3N0A	7PTD
3E0X	2WHF	1AJ6	1TIG	1N6P	4F25	3UUG	4FID	4DNJ	4GOQ
2DG4	3MYV	3VKL	1IS9	1WOM	1KKH	4FRV	1AK0	3FNI	4JGF
3QHP	2X18	1ZYL	1DC9	3QC1	3SP7	2XSE	4ID6	1YZ7	4PEP
1RL0	2ZTB	2WT8	1U2C	2HKD	4GKL	3C5C	2P8T	3D7C	1DMG
2YWE	3E23	1JXG	3K7N	3PPT	1OH4	3A2J	1YD9	3DHJ	2R75
2PW5	3FME	2DKK	1YXH	2CNS	3OZQ	1ACX	1RJD	3W0F	3OHG
1P3R	2J4X	1FC9	2D0B	3I65	3TVN	3D3U	3RYS	4FFE	1Y4K
3TI9	1SZW	3GFT	4FOF	3EY6	3SVZ	2LHB	1CP5	1DM1	1FCY
3AFL	3UGR	4DO7	2CHD	2RHF	4GZD	2C03	2XSB	3HB1	1H75
1L18	4GZJ	2J8G	1GYD	3C7X	1UZE	3VO2	3FSW	2F1Z	1OVT
1BSC	1F9B	2BAB	4E2O	2GX2	2VPI	2XJV	2I69	1A76	1LF7
1HT3	1VBW	1LAW	3C96	1YD5	1FRJ	2HWY	1CTQ	2DQX	2A1O
1BPD	3PNN	1K9B	3U0S	1M2I	1ULR	3E46	1ESO	3I9H	1I39
3NEC	1L85	3V1H	3S8Z	3FI5	4I5I	4FUU	3COI	3QV2	3MNK
3ODY	1CC3	4II1	1B1C	3MKD	1I8N	2QDX	3GOZ	3LIE	2HX7
1S68	2IOS	4KHM	4B3X	2CEO	1DTU	1D3N	3RED	2AMH	2I5H
1BYO	3MX7	3U3R	4BHB	1WDV	1CEO	3OSD	2Y0R	4GL0	1ZGD
3PDD	3V2T	1H85	2OT9	1L79	3VGI	3AOT	2QVT	1WM5	1JP4
1VIN	2G3Y	1OFJ	3AV4	3FS5	1QPC	3QXM	2CJJ	1XDZ	2Y4M
1CRI	2VZS	1A41	3TLQ	4GWI	1XF8	1XDY	2QZQ	220L	3FSM
1V77	3MZZ	2B7R	4H3N	3BHY	1KAF	2OO1	1UXY	7ATJ	2NTD
3RFU	1KU1	2CXF	3QQZ	2OZO	1CU0	1DOT	1Q3X	1A2B	1RB9
2Z1P	3DSK	3TDV	3BF7	1FF2	3CMV	1YHI	3BY0	3O81	1OWL
1QFO	4EOC	1WRJ	2DDR	4E2U	2P4U	2PWW	1I4W	4AM6	3EIJ
2Y5C	3R9C	3G5S	4H4J	2Z0X	1MNI	3EHR	189L	109L	2QMQ
2FWT	3M8B	1NR0	2FNA	3ODN	1Y4C	4EQN	2IHD	2F24	1C8Y
1HXA	1FRK	1FVR	3L0R	1WZU	1DYO	2RBO	1YZY	1KON	4HTJ

3ZWT	3QBC	3IFK	1QWR	201L	3RJ6	4K8I	2QZ6	3AUZ	3KVG
2IZR	3RD2	1R76	2QED	4J1E	1DIN	4ETN	4DOX	1NSA	1HUQ
1IZO	2OL8	4DCU	3IAD	2Y6Y	2EYM	4EKZ	2O7A	1C7I	3M1H
1XX2	1K5V	1UPL	1CKD	2O5V	1Z32	1VGJ	4DQV	1H43	3M4R
2OVO	1C5H	3BQV	1QDQ	3V93	1OSD	2BIQ	1M2M	3HNY	2X5Y
3C2C	4DON	2BIO	3EVY	4IYE	1EQV	1JI6	2RBK	2OQ0	4F1R
1KTW	2XVK	1P6F	1ET9	3MWZ	2UWF	1TBF	1RLJ	1LI0	1Z6G
1BRJ	1LM4	3TVY	3PSH	3ILI	2DBY	1C0E	1RWI	1IMJ	3TZO
1W96	2W5N	3SIL	3CSB	1JNF	2ZIB	2R5G	1BU8	3DHQ	2ZOT
1B8L	1SGL	2GC6	3SRE	4GNR	3O66	3IXD	1P6Y	1B0F	1XP3
2V0C	4DTE	3I3L	1KTH	4GMU	3KHX	3GT2	4B9J	1QTS	4KJO
2IDC	3VMV	2JKB	2VK9	1N7N	2HIX	2W6R	1T46	1BX0	1LMT
1VYD	1QQE	1FIK	3JXS	1JPD	1BNL	1ITX	2PBG	1JAL	1NGN
3ZKG	2AWM	2H14	2YY3	1CVE	1UPV	1VZ3	2JJD	4AF6	1Q6G
1HK0	4GH9	2CX1	2EIB	1N7X	1AGI	2EU8	4EE2	2FZP	4E6Q
2DSN	3TE4	3OG2	1KK1	1IXK	1UGN	1E6G	4KF7	1FFA	3PLQ
3VDZ	3PIT	1EUR	1WUO	1AKL	4G3O	3O0K	3N0U	3QEF	3NED
3EWR	3MLH	4L83	1OUI	1KW4	1QOZ	3LPD	1TEF	2FCB	3N5N
4DUH	1UGF	2PIC	3IMK	2WJR	1A7H	3ZUZ	1Q5P	1QAU	1S2M
1JVY	2Q3Q	2UVE	3TME	1EFQ	2FPQ	1JSE	1Q5Z	2VN4	2ERF
3CIV	4IUS	1J2F	3F7M	1E6N	1OUE	1FL0	1WQN	2CVE	2J7V
3BA9	3RVC	1DTX	2ZPS	1ZVD	1QCY	2HFC	3SZY	166L	3GT7
2OLW	3RRU	3NTK	1GAW	3KEV	1F2F	4DI5	3I7M	4AI5	3RLE
2RDI	1U59	3NSW	3BZ6	2Z4U	1Q2Y	2IAW	3C5K	2XWG	1IHZ
3U4Y	4HHJ	3FW9	3E0E	1AUQ	4AHJ	1CNK	2C0M	3ZSJ	1W2V
1BMG	1UEK	1VJ7	1Q6V	1RQ5	1MCY	4APC	2ZEQ	2A6S	3TGL
1GD6	3EXM	2F0O	3GGY	2D80	1ZY9	2WJS	3HSM	2I5F	1I7H
2FNK	1L02	1ZIR	1DGM	2YD3	3DDD	3RKV	3PH9	4EES	1GB7
1VMO	2WZG	3N4Z	1BXO	1IQV	2ZR9	3SG0	3SBM	3KHI	4EHX
3TRD	2YFU	2BIN	3KFF	3VBC	1EBE	4J0X	2H30	4GOS	1GHM
3DH8	4GPV	2XMQ	3ORH	3UTN	3K4Q	3APU	4E55	2A1I	3GXR
1XK3	3L9U	4F2T	3M7T	1UUM	2OQF	3UFC	1UUE	3OFT	2OO7
1PP0	1LQX	3LOP	1LKI	3ZM8	1GQ4	3ATY	2V7O	2CY7	3O14
3DJN	4A6W	1ND7	1U3G	2JKO	3LXJ	3SGG	3SEN	1E5T	1C93
1R88	3K5J	3CVB	1LLH	2BBW	1LE4	3A5K	2CCP	3VJ7	1IFR
2F3N	2XD1	3PLK	1MBL	2QV7	1VLY	3K0R	3PXM	4DHF	4FKL
3TR7	3KZZ	1GHS	3OM5	4DKW	3VG7	3QRJ	1IJB	3OES	1SK7
1AJK	3KM8	3QB8	1CZN	1PT2	3EKF	4I4E	1KNG	4JHN	4E3Y
3OO7	3UC4	4FG8	2PHZ	2JKF	3I2N	3MPK	1FRR	2H0B	4BL1
2QMT	1CT5	2PPL	2CPU	1EXM	3RRK	2F8L	1CRG	1QXL	3OR5
1YVJ	2I7V	1IM5	3RCM	3AE0	3OOS	3BMX	1D6M	1U2B	3U4T
3D5W	2QDB	1BQE	3W6D	4G2G	3B0J	4AWZ	3BWI	3MPF	3HYI
1MOM	3CGZ	2YOO	3GQV	2QQO	3NRE	1UED	3F3M	1H2X	236L
1VYP	4H3L	3GGD	2XC2	4FD8	3H1Q	3S99	3PUA	4DG8	3SOE
2IUG	1H2P	4EMN	4JV8	2RAL	3ANR	2F0E	2BOD	2VL8	3I7V
3AAY	3NF0	2Z5L	4DOL	3DMG	3USH	4FR9	2FT6	4AT5	1QT5
1OFK	2WFB	1VLO	3I4W	2D2E	3DGK	3P2E	1Y08	3TP4	1MH1
2EYU	2GO2	1FM4	3ORZ	3GTA	4AWM	1A32	1YJE	4HOO	1N4K
3R05	3ZIZ	3HEP	3B0G	3DY5	4PTD	2GMW	3GBZ	2Y4X	1LUF
1XM9	1HD5	2HLV	3JS6	2NYV	3O5N	3TPA	3ILS	3OVW	2BQN
2CWR	2QBN	1WD5	4E6F	3RZU	3QDA	1WSI	2ZVS	2PU3	2V2E
2R16	1LHL	3AAP	2QYJ	3N35	1ZWZ	2WSI	4F92	1LCF	2Y4T
1VK2	3N9K	3QGV	1Y2G	2WUR	3CPF	1YK4	4FU6	3QAH	1PSZ
1QAM	3D66	1DVG	1GV5	1V2Q	1K5B	4AR1	3BS0	2FU0	1XK1
1C9H	2FSX	2CKK	2HAQ	2IXT	1ODM	3M19	1UJ8	1V9E	2CW8
3I95	2BWQ	4ZNB	1HEB	3IR4	1G1B	1UH4	2CKW	1R77	1PRZ
1PYF	2ZMU	2AGT	3ADR	2XSF	1Q25	1WV3	3NRW	2BIJ	2V1M
2O9U	4KD3	3LDF	2ZB4	1NGF	4B2G	4A95	1T9Z	1O3Y	1IP4
1KDC	4DMR	1F0I	3RE2	2W1W	1L5P	1Y2K	1VA6	1GWN	3QU3
3E0G	3SE6	2QSV	1CCR	1Z1W	3ICH	1RFK	3I18	3PWH	1HFQ

1WQM	3V68	4E7S	3OKV	1RRK	1B9O	2F7D	1G15	3CS1	2WZK
1QQM	4KLK	3A3V	2ZPR	3ERO	1HTJ	4EHC	2WN6	3OC2	1SLY
4I17	2W15	4KYB	2GTN	3EBH	4I5U	4AS0	2XIK	1NRW	2ZRJ
1BN8	2OSA	3M9J	3GU0	1VB3	3H99	3FOJ	1GDV	4EDE	1L1Y
2P0L	3D68	2ZV6	2IJQ	4FX5	1L92	3T15	1E6L	2CSD	2BKL
2ZWP	2QZ0	1OXJ	1ABA	1RWH	1Z1L	3LDU	3R4C	1VEN	3PXL
2R10	2JAV	1H29	4H0C	3UFI	2YWD	2X8X	2IYK	3L1X	4G7L
4FPW	1A8D	2HLR	2WPG	1W7F	3DPN	2B71	1M9I	1KZM	4MJ2
3SNF	3ZCY	1QOI	3ED8	1ECA	3CQN	2IVN	1DX9	2OO4	3O8U
3P7N	3EDI	2OZT	1DXK	4EL6	3E2O	1CV6	4FGQ	4EYG	2H92
2OLR	3KBB	2HZI	2AWX	1HUS	3KL7	3CC6	1NDH	2XKR	3H41
3ALM	1PZ7	1X9D	2PNS	3V0D	2W87	4KH8	3CJ1	3L8H	4EVO
3QIC	3I31	1MLA	2YWK	1B2Y	1OXY	1GW2	3F2L	3LXL	3DQY
3TC7	3QGY	1OJQ	2GGC	4DS2	3LDG	2VDX	2HQK	3LF7	1X6Z
4KB7	1JAB	3B0K	1LLN	2P9B	2OCA	1UXX	2ESB	3PSJ	4AVQ
1XB8	2AS9	1OUH	3F2Z	3G4P	1X8B	2XA3	1UD3	2JFN	2X9J
4AAA	3QSJ	3CSE	2ABS	4ERN	3KYF	1HEO	2PWE	3TNV	3C0N
3MF6	2YIB	1OJ4	1PZ4	1GO2	1ZVE	3O2L	3NR3	1TOL	1QCF
3D79	4AC1	3ZNV	1HG7	1BCZ	2FLS	3AZP	2F5X	1UI0	3QOW
1JXJ	2QRS	1QXP	1FGR	4KK7	4FAI	2H8L	2AMY	4HHQ	3SVV
2A6X	1P63	2VAW	4BB9	3DXN	2SFA	1VYF	8CGT	3GI3	2W61
1MDW	3JRR	3MYU	1AAL	261L	1L50	1J08	2EG9	1WP5	3HNX
1JL1	4GKR	4AD1	1GZ1	3UAM	1EJ8	167L	2VI0	2QLE	1RID
4DQ5	2YLJ	2GB4	1RP9	3BDZ	4H2D	4GJ1	3EVP	1JOI	1VIS
1VFD	1N7D	3I37	2B3O	2WNF	1N42	1MMN	2H58	1WIO	3NGP
1CGT	1INU	4DGZ	2OQ5	1YN9	4AXC	3FUT	113L	2Q7F	4EKF
3EIR	4I0Y	4JHT	1P52	1II3	4G3H	3FHF	3BZS	3T8S	3NMB
4AF7	1XHX	1B0T	1XTE	3FFT	4G50	1HV5	3EKC	2WSA	1M33
2DXA	3BKH	1LAX	3W6E	2EBN	1V9P	4E4V	3EJF	1WZA	2B0T
2D4H	3ETP	2BJJ	1CUE	1B3J	3I2X	2BB5	2A08	2UUW	3CMS
4KED	1DPF	3IM6	1YBW	4IY8	4ATG	4EDQ	1M2G	2GU3	3DM0
2IUS	1WLJ	2HCF	3BLP	3COM	2GCB	3SGI	3CUX	1I44	2HGD
170L	1H2E	3EHG	2VQ4	3UA6	3U1F	2EIC	2YK0	1XWB	3HCH
3TYK	1L12	3MST	3MVS	1YNA	4E1Y	2V9V	3IXR	1UBN	2WKP
1MPP	1YII	2MSJ	2RGG	1VMA	2O0P	3UP9	1EU1	2QR7	1MX2
2AT0	4AOW	1GKU	1IZK	3TVD	1IXG	4A7M	2DUT	3FHD	3C9P
1GBO	2X5P	3BAH	3K8K	1HGU	4F6O	1FLU	2P3H	3V3S	1D4C
3QPD	3GQ0	1K5A	4IPT	2HXY	3QZL	1X8Q	1AW7	1VPY	1KZL
2FEM	2CXV	1ENJ	1C69	1UCG	1LYV	1CUV	2XBT	3LH5	1IV8
3NMQ	3E9S	3HDX	3UC3	1B21	3PL0	1UGE	3S1Y	1L57	3M2J
2FMX	2YYW	3K6P	2W9Q	3CWI	3LES	4B9G	3OIH	2HT6	2JDG
2WF7	2YMO	3ZJC	3Q72	3IU1	3NY9	1OT8	1J1N	250L	2H1V
1V33	1WAK	1WMA	1NQF	3KKN	4G08	2HSZ	2FTB	3L0Y	2W7S
1QY5	3W1E	2WSR	1AHO	1NYL	2YBY	2ABI	1L3K	2G6F	4B6X
3V92	2C1C	3TQO	1FHU	2HNH	1GVZ	2CX6	1QUH	2HWX	3VO1
1DCQ	2OKM	3PT3	3CPG	2CE2	3LNO	1L41	2P2M	1VSN	1E0V
2VKA	1GVL	3GQQ	1YLH	3VU2	3VMT	1YZK	3F67	1ZHX	1CIP
3R3Q	1PV7	3NO0	1VFL	2XI1	3U87	1RT8	1ST8	4JXM	1JRZ
3IC4	1EAM	1Z07	1BRN	3F8T	5NUL	3EE9	2J8B	1FGT	2YWQ
1WR2	1UGC	2UYT	3IHW	4I8H	1CJA	2AX8	3D30	3O7I	2B91
1FFR	3BQE	3GNR	2FV8	3EW7	2LIS	1YV4	1CYF	3G8V	4GJZ
2JEK	4FLR	3FJ2	2V7Y	4FTD	1XW2	3ITG	3MVV	1IO2	3S1E
3GHA	3QVM	3RE9	3RU0	1JD4	2VND	4GVO	2OHF	1PTV	3MF9
2C8S	4EE9	1HA5	4AGK	2YD4	3LL2	1WXO	2X97	1WCZ	3D6C
1F0N	4AUP	3KP8	3C1U	1F5J	2GF0	2AH2	2X32	4HFO	3VTE
4AFV	4AY1	2YM1	2RJM	4ATT	3A0Y	2A9V	2QTD	2IJF	3CWK
1CGL	2VZM	4DWW	3ADG	3LKD	1CQD	2Z6G	3VI8	1IUK	1EAX
3DLW	4GSO	2EU7	4JYF	2XWV	3S1S	3FG7	2BAA	1L60	4G7F
3UBD	2BK8	2XY4	110L	3HC1	1L9C	2DUI	4FNX	2H6D	2IC2
3HZC	1UIO	3VRQ	3ZHW	3LYS	3SG1	3B9Q	2H4N	1N8V	2RKN

1MG4	4KBZ	1A5Y	2QQI	3G4D	4HXW	3ALJ	1G07	4F6J	2AR5
1E6H	172L	2BLC	1GPI	3TZG	3TOB	1UK0	4INW	3HMB	1C97
3CLJ	3CW3	3FEZ	3GBO	3V53	1SYD	3P3K	1UKF	2ZWA	2XI3
1FCQ	1YIV	3ZXK	1ZKJ	8ABP	1PNF	3CKM	3HY4	1U9U	1W6X
3NQH	4EVM	3P02	1QFJ	2FO0	4E56	3EC2	2I88	3ZIJ	3MUH
3I6V	3RX9	1RP4	3GKA	3HCI	2WMK	1NEP	2E9L	3LWA	4FVC
4DT4	2ZEZ	2YWI	2HVL	1E6K	1ZAU	3QSL	4AE5	210L	1KNI
2VAC	9CA2	3MFR	1TR9	1L1E	1I71	1QQT	1C6R	2FKG	3SXL
1NFN	3A99	2P9R	1H4M	2RJV	2XQ0	1NP2	3GI1	1W6J	2WOL
235L	3BUU	4IFE	1FFB	1MLW	4I16	3L5H	3FDH	2F28	1J36
3B0P	2FC3	2WFO	2D1V	2PX6	234L	1BYP	1IO0	1FCM	173L
2GSO	3A4O	3NVL	3U67	1A2P	2A4V	1YOL	2PNN	3TUG	1S01
3SR9	2XMI	3E6K	125L	1GOU	3SQR	3LQB	3A24	1UIB	1PZD
1WDQ	1POA	2BOU	3Q1N	1U6D	1PZP	3OFK	3L9T	3BI7	1EOF
1U9C	2XSP	4IAL	4A0P	3K9Z	3OAI	2BL9	2C2N	3LS3	3R7A
3NR0	3S6S	1YOJ	1MHN	3O8P	3N2E	1OTO	1HQ0	4L5H	4FYO
1TAZ	3O5S	2AZO	1RBV	1L8K	2RFF	2CHM	1H5Y	1MAI	1SFE
3VYW	4GUD	1HG8	1SNC	1R2D	1POH	3QF6	3QXL	2VTC	2ZPA
3HM5	3GMI	3I0A	1QOB	3FKE	2R6J	1P6P	3IUS	2ABK	1Z8O
3P3C	3D61	4FHE	4KJN	2P6X	2E0W	2W1K	1BRH	3AB6	1OB0
3N1O	1N6C	2X7M	2QRY	3MQZ	3BCK	3MQ2	3CCF	3C80	3CVA
4KAJ	1O7T	2RE3	1XM8	1BT0	1LAA	1SEG	1B5L	3PO8	4ELO
3E59	1G94	1L62	4FA0	3AYC	1P3D	2ZQT	2C29	3JQ0	4HQD
1LI5	3HFW	3PQC	3HJL	3DA1	1F00	1E39	2ESO	1J0P	4GS4
1KGW	4AHH	2NT3	1BC1	3T6K	2RKX	3MFA	3GRU	1UXO	2OKL
3HKO	4DQD	2IF6	4BLM	7PTI	3UL8	5MSI	2Q8P	3QYE	4DGX
1YQP	4AXN	2WUG	1BUP	3PGS	1GV4	3T8V	1RXI	3G89	3U0L
1VKB	3EYG	2R1B	2HUO	3OTX	1B0V	3B3D	1T43	3FDJ	4EKL
3EC3	1NCJ	2BQF	4JG5	3BD3	3SHQ	4AHC	3MH3	3QXW	1EY6
3DAU	4I1F	2C8B	2RKM	4E1S	2F0M	2Q9D	3QYB	1F0X	2X26
3F83	3P98	2IWA	1MUG	2FAZ	1XZD	2BT6	3W6M	1WE4	3TB7
1KTG	2NXT	2GL2	3T9T	3KS6	3NT8	2QG1	1CKH	1KG2	2RA9
3RDR	3ZH6	3HD4	1T9F	4ASC	2FMA	2PYA	2QDZ	4F54	1URR
1L8B	2W50	1J2A	1TMO	2EAD	2PDY	2RF0	1IP1	1Y59	4AX1
4JEI	3MW3	3GDB	2Q9T	4AW7	2FGO	4AMC	1A2O	3NAX	3D0K
3ND8	1G6H	1JVJ	2EA3	3JZZ	147L	3AGT	2O9R	2GK6	3L2Z
1JZI	2BKX	3TNK	3CB5	3ER0	3NYX	3KGD	3JXH	2FG5	1DKM
3AI5	3TPO	1VJN	2IJ2	3FQI	4GJX	1CZP	2YJ7	4EIH	1GB2
2ZCA	1D3J	3VSE	3RGM	3FNL	4F67	1DKK	2OVD	3D4D	3KZX
3TB5	1W9P	3RNS	2BI7	2WHM	2ALA	199L	2V4V	1LYG	3KUQ
2R8B	2ZUJ	2BNH	3FM1	3IDU	2ZF8	2Y3V	3V7L	4ACY	1J6R
3EK8	2VCL	1M59	3C8S	1AOP	3O3T	2OW6	3QZR	1TS4	2XWO
2HW2	1QT3	3RG0	3GUU	4G17	2IXN	3H4X	2ESP	2A1L	2YB1
3VUE	2CJP	3SAD	4DDP	3MD9	4G9I	4FR4	1VYS	2RJN	3CYI
3NWP	2VZO	4DMO	3FTO	4ID3	1MB8	3H6E	1XAU	1YAP	231L
4BE8	1QT8	4DIX	1P2R	2G62	3ML5	2XWR	4AGJ	3ZYW	4HJK
2Q8K	4EO0	2IOR	3OJW	3HTG	1TOO	1MD6	1W42	1R4B	2ASI
2ENU	3CYR	2ZDJ	4GSR	3K7C	3L3F	2V8I	2CIO	4J3M	3CRV
3R69	3MS8	3UMS	3SY9	3SCY	4EAT	1BIY	1MOO	4GFQ	2YWZ
3FYD	4GAW	1GBY	2ABB	3RKX	2HDZ	2AWK	3DTK	1N6O	2QLI
4I5R	3OBL	1XAP	1XG6	3RY3	1MUA	3FW7	1ZB1	4GQ4	2ENW
2ATM	3C5H	4ESU	3I1F	3SG2	4JGK	3GN9	3A4R	2XOA	2D5J
3URC	3JRN	1HCB	1R6J	1ML9	1BIC	2YG7	3V46	1S14	2RH3
2WIM	2FAV	2BM0	2OUP	2PYK	2IOJ	1FNF	2O87	2A01	1E1D
2FOZ	3OKP	1R5M	4HQ1	3NPX	2BZX	1LJ8	1UKP	3BS9	2W0G
5EAT	3MZF	3ADZ	1L0D	3NOH	1YK9	3DXS	1RAL	1KXD	3GIP
3M1W	3L23	3OP5	1GBA	2NYP	1SXX	1Q7R	1YSQ	1C83	2Q43
3ZH7	3H0F	3M9Q	1L75	4FPX	1VRX	1SNB	3KHE	4F4F	4G37
3ALF	2WSD	1ZIC	4FGD	1E8Y	3RVK	3PGU	1ES2	4JN3	1DRJ
1A70	2Y6X	2C6Z	2OK3	1IFC	1X3K	4ACK	3Q3V	4ETI	3FZY

3BAO	1GND	2P61	3E5S	3GM3	4A3O	4AVN	1ZCA	3PQV	1JR2
3NP3	2Y0H	2FPH	3FFV	2X1V	2J42	2OIT	1LBA	1G2B	3O1Y
3FAY	3NY1	1YK5	1WMD	1KR1	2Z47	2ZRQ	1CCP	1KHV	3AGK
3IT7	1VCP	4FR7	4F7K	3LDO	4H3S	1D5C	4I6X	4KRR	1JVW
3BWH	3T7H	2BZ7	2CS7	1S0I	1FKL	2RG3	2BF4	2GX6	2AZW
1CYX	1T1U	3SHU	1SVU	3A7C	2FGN	3I8U	1LHH	3TEB	1SNZ
4AQK	3TBN	2YZ8	4FYB	2MEI	3DHA	1RM8	2BCH	1ZYO	1KVW
2J0V	1S3P	1W9J	4AU9	3C83	2W7Y	1CV3	1OTM	1F98	2XDY
1L0G	1RA9	3KZS	4HWD	1YE8	3UEM	3MX3	1IX0	3JT2	1Z6I
2V95	3PHU	4ETT	4IQF	3MJF	3N0X	1CGW	2ZE4	2W1N	3UP6
2OSX	4DY9	2Z5J	4MN0	2NXF	1XCL	2YN2	2WIY	1EX7	1XV5
3C1E	1T9H	1XZH	3V3G	2H34	1B5V	1ATG	2V70	2ZCV	1U9M
3GPI	1FUS	2X5L	3E53	4A3U	2G2X	2BJQ	4ASE	1QK8	2XB0
2HFS	4JX9	1UP9	1YMV	4EVQ	2BVV	1K4Y	4FMZ	2EUT	2FVV
1URX	2PA1	3CB7	1ZHP	1KHB	3HFF	2NYA	1RN4	2VL7	3HSJ
1H6L	2VIX	1DO1	3SOC	2RI2	2ZQE	3CDR	1BRF	1VLP	3AGN
4ABY	3MC2	4F6H	2PZG	3GYJ	4A02	4DHE	2I49	3FUO	2EYH
3MP2	2YNK	2RI5	3EMU	2O6Q	2CXY	3SUL	4J93	3RF0	4FGF
2NQH	3VHJ	4E6X	3I4G	1E2Z	4IYK	2I5Z	3FRT	3V50	2QDF
1RFS	2AD1	1NGG	3UL9	3B6Z	3DJM	3ODO	1KW5	3HVA	2WAF
4K60	2C78	1UMK	4GYU	3HN7	2Q3O	2C1X	1ZCE	3LLO	4E5A
1XWY	1K7J	3RHY	2IUX	1LY2	4E46	4EI7	4HDT	3I4O	2X8S
1XGK	4AN8	1H7S	1CPW	3HVI	1I3F	4FE3	1OI7	2HQJ	3UTK
3KA7	2YG6	1KRJ	2OTE	3IEI	3NEY	1JJV	3SLO	3AJG	2JI0
2HZF	2DQ7	1PJR	1ODZ	2W5F	3LM5	3GDH	4IUW	2AEZ	1LR7
2JH2	1TRH	2YGE	3NV6	2CIP	4EFP	3MZS	3UHM	4FIH	1Y51
2BL7	2GTG	1ICX	1JG1	4DWR	3TXZ	2XKW	3FGS	3FN5	4HWM
3MNH	1SUJ	2IPM	2PQT	3VUS	1OUB	2X6U	1TFF	3RBW	4HVT
2D3Y	2OUS	3UV2	3TP8	3O7W	1BOL	3R4S	3LML	2IJ4	3ZX1
2VSE	1TRI	1Q46	4HU4	1SD0	4FFL	4JJY	2FBA	1GY2	1O6G
1F2Y	3T54	2E26	3BDI	1JF0	4KNV	3KLK	3M1T	3PG0	2EWI
1XZF	3D2A	4GKY	1MXG	1MRH	1TWE	3TFF	1OW1	3UY7	3H75
1XGD	3RQT	4KBL	3EYE	3QXQ	2E7Z	1SNP	2ENG	3CDQ	1DTL
2R89	3G7N	3KK7	2H4R	2OXB	1FQL	1V3H	2XJK	1AHR	3FDW
1W9L	3JUE	1FV9	2ZV2	1EYH	2WCJ	3FM9	1BJE	3LPC	1QF9
4BWR	3MTT	3TM4	1IM4	3R72	2ZJY	3HMS	2CNC	3FVI	1QZM
3OPT	2BVL	2W00	3VAS	4F6C	2PND	2YIK	3EQE	4HTT	2HI4
2ZOX	1Y0P	3LM8	1Q8Y	3VER	1V0O	3D08	3RP8	2HSH	1SIG
2GH4	217L	1WPA	1BGC	4DUE	3O48	2OV7	1I9Q	4EIE	3GUG
1PRQ	1RRF	1OOT	1Z53	1T5C	4EQK	3CLN	4DRT	2C8M	1QSA
1V2S	1ZI0	1BC3	2NTP	1EYN	2ETJ	2VIM	4GE6	3OSX	3FRX
4GA0	1WDI	2Z8X	2ERW	2P51	3ROR	2YA2	2JIE	2HQ2	3TI2
1IQ0	2BV3	2WAA	3CO1	1RPJ	3KPX	3B55	2W51	3DV7	3LKY
3GYL	3HV8	2EW1	1QNT	1CUY	2XIR	1LQJ	3AU3	3M89	2PV4
4GEY	3Q6K	2UVJ	3ELI	1CGX	1DI3	1UV4	6AME	2V5D	1T07
3HJ4	2YQ9	1XK5	3LLC	1XQR	3RJD	1BGE	3CXL	3NGQ	3K1W
3S3E	1RDC	2MGJ	3PDQ	2SGA	3JVI	1R62	1IPC	1QFM	1J1Q
3OWG	4J9T	1GSO	2JLN	3HWN	1FNA	2FCP	1JNU	3GKM	1UEJ
2Z7R	3T0O	1QDM	3R67	2IQ7	3I4I	4A3P	1NGE	1I92	2P3X
1KY3	4B3N	4EV4	2QEN	2P3S	2CGQ	2XRG	4GGP	2FOL	2WLR
3AGL	1TTZ	3DWO	3W7T	4DFI	3EG2	2CX7	2R4L	3O13	3C1F
1DT2	4E2Z	4BIB	3U3B	3F65	4J87	4EMI	4F5Z	3RAZ	2GWR
1CII	3K13	1COL	3CD1	2HRS	1GIS	1EQE	1Z2Z	3POH	3UIF
4HA6	4ASK	4ITJ	1UYN	3FWJ	2QN0	1R5X	2O62	3FFW	1EM7
103L	2END	4FK8	1YVU	3T95	1EXN	3MSW	3NC3	2E3I	2Z72
1UY4	1ZSC	2AZ4	2P20	3E70	3VGZ	2QF4	3NZK	2GMH	1D3K
1LE6	2XNE	4IDR	4BD4	2V0V	2PT2	3NVX	2HEU	2D54	1PVA
1EQ4	1T95	4A66	1YZN	1YFO	4L8K	2FP8	3EKI	3VHN	1GSV
1TVF	1XZC	1L71	2QUO	2WL1	2W3W	4GR4	2OF1	2J8I	1JPE
1JT4	2H5C	163L	4G9E	3AAM	3FLE	1F76	4IXC	3QOU	3IF7

1AD2	3K7G	4EUZ	3DT8	1C9M	1LI9	2Y5P	7AME	4DIJ	2R6H
4AMQ	2IUJ	1VHU	3HM8	4GMQ	2WAW	1CUH	3AP9	1NF1	1H19
3MAO	1LME	3IM9	2B7S	1JD7	2P4O	2XHG	1P2H	3IM5	3L7N
4GW3	3AOF	2GHI	3A0F	2RB6	3U2P	4G2P	215L	3S2U	3KN6
2PCS	2B4F	3SUZ	1I19	1CTW	1T7A	2BAX	1ZD9	1VC9	2B96
4KY6	3E9V	1CV4	3HP4	4ETU	3QQ7	1YTM	2I6J	1YZG	2H5Q
3LUR	1OOJ	3ZBD	2HWV	1FVX	2OF3	1XZG	1CLM	2JKQ	2OY1
2VOV	1D3W	1XZE	1TWM	1UCS	3RI8	1ALA	1NWL	4B9Z	2XUZ
1X8M	2WUU	1F0J	3FLL	4A37	2IDQ	1KAZ	4FS7	1IG8	4FUQ
3DFF	2ES9	1WEI	3H92	2F9L	2HEC	2QCY	3IG9	2Y9E	2OKT
2I4A	4A2E	1PA1	2RFL	2OUA	2VAP	1DBI	3ZLW	4GEO	2WZS
2C9K	1T64	1W78	3C0I	2B1L	2OM5	3SS7	1GYM	3OV5	3GBL
2HVQ	2OLN	2AR1	2VGD	4FWY	2V94	1DUI	1ZVT	2HES	1DYQ
1DST	2E3B	1HZG	2YDZ	2WMF	1BK2	1H4W	3IUQ	2GFO	3ONH
1LZ6	3DDU	3QHN	2QDV	4AM1	4G26	1BNS	4LER	4HWY	2ZUY
3AA4	1H8K	4BDS	3MCF	3NUQ	1GEZ	1YPX	3GF8	142L	2ZJM
1MI4	2R2I	4K14	1IOR	1GOA	1I6Q	3I0U	4L7A	2O7U	2WJ6
3NDA	4KAP	1V4A	2I0Z	2QYQ	1XRO	2XVP	2DRI	2XB1	3IPW
4HHX	1XRV	3KGR	3RYY	3OFM	3UID	3I45	1GOJ	1RL2	1DR9
3N2J	1W52	3CRR	3MCM	1WHZ	1CYJ	1L86	1QGJ	3KEZ	3R44
2Q3R	2DUE	1XVY	1QSQ	4BFG	1HHS	3NFI	1IFG	3M97	1YU5
3U3G	1TQ3	1OPB	1QL3	4E2X	1PY4	2VYZ	1YT3	3UOX	3A2I
2XWA	1ELJ	2R9Y	2JC5	1KO9	1EPY	1CJ6	1BIT	3TPT	4AHD
2VUW	2VEO	1MLO	2Y0K	3NA4	3JR7	1SVI	1UGM	2X9B	1VJV
2WXW	1Q35	3NLC	2ETC	1DZK	3VD8	1QPK	1QYF	3QU4	1MFM
3G7X	2QTF	3AQO	3LC9	3EC4	1GDX	3EUD	1BK9	1PAZ	2H1R
3SJZ	1Z5Z	2E0Q	13OL	1NNF	3GBA	3ZIK	3GXB	1OUV	2ZUX
2GS8	1A26	3NS5	2EAB	3ALL	1R55	2VZU	4EDP	3LS0	4AMO
2QQ8	4G75	1CRZ	1GM6	3OSJ	2OVJ	2HEB	1K7G	3Q2C	3ZS4
1DZA	3OQV	3SEB	3UYQ	2CN2	2QQM	2EY2	4A0D	2YL2	2WJE
1JO0	1SVY	1MOD	4B6M	3PVH	2O8U	4FG5	2PSP	2OUO	1UJC
3GO9	4GX7	3QFR	2I6P	1LG5	2Q3J	3JW7	3NHN	2EYI	155L
1L22	3ZJX	3NTS	4EQP	4DU9	3U1R	1TXO	2VO8	1Q8D	3C2H
3BCI	1T7L	3KUH	2HJE	4E32	1IKQ	2ZRR	2DKV	1IN5	1P3J
2OFS	200L	2Q57	4KWU	3GNU	4K5W	1AK2	3MWN	3CV8	3C73
2IM8	3B8A	1GT6	1KS5	2CJ3	3MGX	3IV6	2Q99	3KLR	1NST
4GOT	2ATZ	3KT5	2RJ2	3O5J	2IBB	3FW3	1B0Y	2HCN	246L
1HKB	3KS4	4EUO	2F0N	3RDY	3III	3TL4	3TZM	1H3N	1OBO
1ZBT	4IT8	2ZJI	3IPJ	1CSV	4L11	3QI7	3SG3	3NVJ	2DRU
2I24	2R4T	3GRE	3IB0	3I09	1RV9	1CEW	1O5Z	1FRI	2IPL
2REU	3BZN	2A1N	1Z1A	4APM	3KV5	1URM	3MRS	2UUT	1RYN
1GR1	3QQX	2DT1	4HD9	2O0A	2YPC	3TCA	3HP7	2J1W	1HXR
1S4B	1HUY	2OU9	4F7Z	2X7Q	3PP6	3V5M	2H6Y	2JHX	3HYG
4DNI	2BEP	1LFK	3SR0	1TDW	1D4N	3AGP	4HD7	2D1T	1QKW
1H7M	1JY0	1KHY	1UIE	4HMM	3NQX	3HDF	3UGJ	4D8K	1QMY
3E0U	2R60	3PZG	1GQ8	2GUI	1PZV	3B49	2H2Y	4DXD	3PF1
1DI1	3CED	3B4X	1PME	3AVS	1P7S	3EYC	2QNK	1YLW	1NCN
1J99	3PB6	3EF1	2DKA	4EYV	2YFX	1LVG	4E50	2CEP	2W0B
1N7W	1ROW	3L00	1AD5	4I69	4HSD	1F2Z	2WQQ	2YH3	2ITN
3MJ6	1DQY	2D3D	4HGG	2ZK9	3SDN	2V0U	3NKJ	2DEP	1SQW
2XU9	3C0C	1S2O	3RGN	1PX5	4EUE	4FEY	1R2Q	1XR7	3PLC
3I57	3OBF	2WQE	1JTA	3TF0	3GP0	1RIF	4DNY	1MBM	2ABW
3RP7	1MIX	3MDM	3EB9	1S3G	1O2M	4A60	3ULJ	3O2T	4A2S
3AQ9	1W9F	3NZ1	3H6M	1OKC	3P8I	1GB9	2W4B	2HS6	1Q7F
1JKD	3SFK	3DCY	1R4X	2GF3	3N0H	4AYO	1SW2	3L6V	3AV8
1TF5	2WMG	3VTV	3ACH	3N0R	3KOG	3JYI	1DYK	2D3V	3M03
3FEK	1RHL	3C0D	1XT0	2O84	3LCC	4AEM	3E0H	1FB6	2XIL
4I68	243L	4I3D	4DJM	3NZM	2PBC	1RSE	2CP4	4D8B	1HJQ
1PA2	3TVK	3RWM	129L	1VSR	1L66	1YIX	1BRU	1YH3	1G06
3ZYZ	2JLS	3TEZ	4HBZ	4FVM	2II2	2DW0	2QON	4I5H	4BBK

1YZ1	1FDQ	3FEU	1FY9	3BWT	3AMN	2R9V	3LFU	3LX4	2Z1H
1SYT	4IQP	2VH7	1TRQ	4FDG	4KP6	1L5T	4GPM	2HC1	3M3A
3I06	1WDS	2GCA	1L9X	1SNQ	2OEV	3ZLX	2J6V	2O9P	1BY3
2J43	3THI	2F0G	1B1E	1HD7	2WK0	4J2U	2YB5	2GEE	4KKD
3CKC	2IE8	4EY0	1WQG	2VXZ	2Q07	3GWG	3EWD	3ASI	3FNR
1C7F	1NWZ	3GOY	1T4W	3CSG	3LEW	1NHC	2P1W	4ASM	3IM8
1WSH	3FV8	2PPO	4FS8	2F1Y	3UC2	1QUO	1QOA	3KT9	3MFY
3UC1	2GGZ	2O1E	1UN4	2PTD	2P0N	2OS3	1A53	3EPZ	4GDJ
3VAA	4AX7	3QNT	1O16	3SNK	3KZD	4E3K	2UWN	1N9Z	2PSB
3CSK	1WOU	3NNE	2VSN	1N5U	1YN3	1P64	1S02	2R4G	3Q3U
1S3X	4HWH	2VY9	1M4L	1L8R	2Y22	2P9F	2WO4	2IUK	2XXP
3HLD	2UYO	3TC5	3VOJ	1IHN	2XMO	1WRI	1JWZ	2Y8R	3ZDP
3QL0	2D58	4JXI	3V98	1SH0	1AZO	1TXL	1IKO	4JIC	3IUK
4GC1	5CHY	4I20	3HIL	3QDH	1T7B	2XFD	1HRO	1UT9	1E6M
2R5X	1KHC	1RRA	2E3V	2GOI	1PB8	1EW4	1LF1	1CIO	1L47
3AUB	4GBS	4AK2	4G0J	2B6M	3C7F	2VQU	1JSS	1GOC	2OIX
2V7B	2GHC	1AT5	2Y44	2H5A	1XJD	3LJ8	1CJC	4FBS	1DT4
3ZI1	3N13	2OWC	3ZGL	3H1G	3NHM	1GF7	4GAI	2G8C	3HFQ
1CM8	2OCI	3CGG	3P6J	3ISY	1UTE	3P3V	1DJ1	2IEE	3SER
1V73	3CH0	2P57	2ZZJ	3PYC	3UOM	4KKN	2Z8Q	4ES6	2PMK
1EGZ	3PBI	1OPS	3HBF	1PQI	3EPS	3FP3	1GZ7	1XQV	3LP8
2IAQ	4I5V	1BG4	1OQW	2H71	2BBR	4G9R	1RZ3	3IRA	3UWT
1T2I	1JFR	1F1S	3DRN	3I19	3ECI	3VA5	1ZKL	2WOC	1MJN
4A4K	2C0K	1UKO	2BHG	3KMV	4HS7	4AZ6	3S0G	2IFT	2F0K
4B4H	1ZK5	4GE1	3EEB	3ELX	3PGR	3V8J	3AAL	3H38	1TYJ
3PAO	1UDH	1SIF	4A68	2Z1A	3IM1	2F0J	151L	3GA4	1APB
2IC6	3H9X	1LC0	1I1W	1OLR	1UV0	3PLB	2CKG	3IMN	3R26
1OKB	1LV2	3BTA	3H0H	1CLC	1LHJ	1TG0	3LLH	4A78	3IEE
3OJP	3BWX	1C91	4EVS	2RI9	3NQ2	3OUL	2Z0M	1KNP	2I1Q
2VQ9	1BXV	3K8W	1MYJ	4J5Q	3O5D	2VE3	1CNS	4I6O	2IPQ
1U2P	3TXN	1KZF	1YUW	3A1F	1FQF	1ZOX	1WRM	2J69	1PMY
3LHH	4JZS	3LET	1N0U	1UGB	1BAN	2C2Q	3DZJ	3V3F	3OG9
1HZI	2FIA	4IPA	4KV7	2CU9	1GPP	4FWB	2NSZ	1K0M	4ELL
4A3X	3CNR	3LUU	2WZ8	2J49	2X2Z	1IBQ	2PFY	4B1C	4IDL
1LMQ	2R2Z	4IN0	2J3S	2DBN	1COT	2VGN	3L40	1QVJ	2W70
3JT0	3R5T	2V9S	1ZDE	3AG9	3TRE	1HYP	2WJ7	2NRT	1L0C
3KR9	4HGF	2QHA	3FI8	2D81	2OY0	3D4W	2BYG	2GGS	3RGO
3DCP	3P43	3CSP	3LVZ	1OPD	3P6A	1X1R	4ARO	3D14	3BR8
1T6E	3HEN	4AQO	1IJR	4ETX	1N11	1EYA	1IV9	2YN0	2ACY
2EJ9	229L	3EC1	1XMC	2GG6	1H8L	3V7B	2NWY	2NYU	1QS2
1BNE	3T7V	1TR2	3QYP	3LW2	3ESS	2VO9	2MGE	2ZYC	3SUM
4DNQ	1WCQ	1EVQ	4F13	3HC7	3OT9	2APJ	4KVR	1ZCB	1Z0F
2C7S	3QAY	3UL3	1OZ6	1GFH	1ZVJ	3TQQ	2Z70	1JKC	3RVM
1L11	1MAC	1HSE	1XGW	1G0J	3TTJ	4GXQ	2RN2	1ZR5	1QAZ
3BH4	1M8A	1CUF	4HNO	1A8Z	1D2K	1QQC	2WLB	3UPE	2B4V
4FGV	4LIP	2QP3	2A6V	1QPD	1TP7	3HD2	2Q98	3K89	3LPY
1OB3	1A06	1BJ9	4DXG	4F8L	2IOV	1UR4	2ZWI	1GF3	2O3F
2PF5	3OEN	3D02	1ZEL	4D8L	4E80	3OO1	2G0F	3MUS	2VZC
1MD8	1H1X	4F0F	3KD3	2WM8	1ICP	3TG4	3QB1	1WAD	1MQB
3VPP	3UJD	2P2J	1I5G	4HL4	2YX0	1XKH	3TM8	2QR3	2WA0
4G1H	1IP2	2GVU	4F9U	2Z9T	3EKB	1Y4Y	1NIO	4EMV	3SV0
1CGV	3POE	2BJH	3MGA	2LIV	1DQG	1UMH	3CBN	4B6G	3SI1
3LWX	3ZQX	2APR	1ZYV	1ZVK	1KUX	4IGO	3GWF	1THX	1W34
3AK5	2QO5	4GIH	3P1I	1OBV	4L1M	1TZD	1GE3	2EX0	3LAW
4BI1	3KNS	1JHS	1OID	3DLM	2XKG	1DV2	2GFG	4AQS	1FFC
3CT5	3I3C	1QTB	2OO3	3A8T	1W90	1YZL	3MJ1	2F1N	3U0R
1YWR	1YI9	1MX4	1EO5	2BB6	3P74	3OST	3P9Z	1Q36	1HTZ
3C8D	1SFH	2VAK	3D5V	1BPE	2A9O	1GH4	3QVP	4FXL	2J22
1NRF	3BQK	4DQJ	2WZL	3U3L	1LF5	3GC2	1MTZ	2FFI	3EJW
1F0M	2H0T	2Z2U	1VQZ	2CD7	1G9J	2R5S	2ZYO	3OLH	2X7B

1L00	3PJJ	1Q2L	1LK0	3GX8	1DKL	3PDG	3QFC	1QJV	2F8Y
1CM3	1LFH	3ZOR	1TT8	2R61	4AMM	1EYP	3EBV	2CB3	3CYM
3HXW	2G0W	3O9N	1ID0	2OXT	2QRU	3N6O	2W4J	3PZF	2XOM
3JXV	1G49	4KHV	2JD9	3ZSE	1DDJ	3M00	2IGV	3KAC	2VY8
4ADP	4HV0	1T4Q	3AG7	2PD8	3U4K	1LB9	1D0Y	1STQ	2QSK
1OFL	3H1T	2FNW	4HJH	2C83	4M53	2W91	1US5	3VHG	102L
2G0D	2O0M	1TPY	4AV9	3F0U	4D86	2BYV	1GB5	2J09	1UAI
3K6I	1K7Y	2F23	1PKO	1W6K	2BOA	4A8O	4DG5	2YXC	2NVP
2ERA	4H4N	4IXE	2Q0D	4E4B	3NCW	1DMS	3KOF	3NSC	1ZIV
2H7W	2D1Z	2IYL	1FG4	2YKK	3IPC	4FVR	1MLU	3HZ2	1E18
3BUL	3QFP	2OP2	3BOJ	2XD3	4GQ7	2J0P	1AZL	1LFW	3UV4
2CI1	2DE3	1BF2	1HY5	4FJQ	1CB6	4AHG	1LU4	3PYF	1PS9
1E6Z	1WSD	3HUT	2WWC	3I2E	3H7M	3N8I	2R8U	3MTS	3GK6
1PQ7	2WFG	2Z2E	3FA7	3OC6	1V6Y	2HCU	3ONC	2PQG	1U2Z
2ZAA	3B86	1PPA	3C7L	4IQK	3DV9	3UP8	3MYC	4EPP	1KXF
2Y3U	2O0I	4AKP	1YWF	2ARY	4BCZ	2GNP	2PIA	1QI3	3Q7Y
1N45	1LOU	2ZJO	1BR2	2ICS	2QX3	1KT6	1G6L	4F3H	3G5C
3U1O	3T49	3DKG	1ZRZ	4GV2	115L	2BXX	4D9V	1Q73	2QLC
3BIY	1Z6M	2WHK	3SMZ	3AZY	2JCP	3AU2	3IQD	1R44	3UKJ
2REO	1CIK	1CH7	1G1T	2XH8	1M61	3QLN	3FP5	2MGB	4FC2
1J7K	1J0W	1EHC	4J6H	4EII	2QX2	3C82	3LO8	3MU7	1D5T
1GNY	1ZG4	2BIV	2WX2	3AQU	1AYD	2PTH	3PO0	3HMV	3B3R
2Z2Z	1V0W	2XOD	4H3M	2P8V	4EEU	1EFT	3ILZ	3VTG	2OL2
2PF0	1KY0	3BAU	1S4Q	1AVT	3GZK	1CUW	1IWJ	2O5R	3CG1
2DYJ	1JD9	2WGR	1I5P	3RKP	3ETI	3Q2D	3TPK	1GWT	1UA7
3E9E	2JHN	1WF3	4B47	3CXG	3V44	1ES6	3I9Q	1H4G	4ILI
3FRN	3LA1	2V5V	3A4X	3G98	1U6T	3MA6	3PT1	3OM7	2DRR
2V6V	1IYN	3P67	1UD2	4F3Q	1NQJ	2VSF	4FKD	1QUI	1SCH
3QU5	3JWG	1BH2	4F9Z	3TEW	4FQE	1UOH	3IP5	2OC5	3MNQ
4EX4	1SMD	1BSW	9MSI	1FMV	2ZPQ	1W8W	2P0C	2GKL	3O7T
3E2N	2BVZ	3DWM	1DY2	4J0D	3NZL	1DK7	3SUB	3FWH	1ULW
2BDI	1NTN	3IIO	2Q22	3O0A	1YVR	2VH9	1DS1	3HCJ	1DG9
4H1N	2YCU	1HEQ	1LB4	1GEV	3SHS	4ICI	2E5V	2GHA	3BNC
138L	2NQC	2CXC	3BNE	1BQK	2OBI	3TU1	3E4Y	1WBE	2YLX
3NNG	2VPJ	3QJ3	3ZXF	1MQ4	3FGZ	1K4N	1N8Y	3IQ2	3GJY
2ZOS	2FFA	2X4F	2QB5	2IAY	123L	1M64	3QWY	3FKM	1BH6
4A9C	1XWI	4EFM	2J5A	3R0V	2QJZ	4JQF	4AFC	2ODV	1ATZ
3GSZ	2YDM	2Z2X	1VCY	2PWF	1VEP	1C9W	3G1J	1OGJ	3FJP
3NPD	1H70	2JFE	2GKG	3K6V	3KNY	1GWO	2F0V	1UA2	1L76
1HLB	2VY0	1I7E	1QKX	4HMI	4IPM	3F4S	3MFD	3ZUI	1SUP
2WY6	3S30	1NOA	2W6X	3G9D	4KHO	1B6G	1VRM	2AIE	1ENH
2AQ5	2RB8	1Z2A	3DAS	1EY8	1KF0	3SJO	1RLW	4DWE	3JQW
4BIR	1KQR	1QRZ	2QP2	1O4Y	1XTP	3QAX	214L	3H04	3UJ9
4FMS	2F4E	3PMG	3SQF	3F78	1J1G	1SXW	2X1L	1U53	3LX5
4AHE	1OO8	3P0B	3GEF	2KZM	3PJX	3G07	3BO6	1XTD	3PBK
3SIG	1H2Z	1SO7	1FTK	118L	1XJ0	1DQ7	2G6B	3EMX	4DSD
1PCV	2HJO	3GXW	4EQB	2XXK	2D44	2MEF	3ED5	1KS8	3L8C
2XCL	4FHX	3ILN	1VHN	3SZE	1SZN	3PTD	3FBL	3NO8	1MDQ
3Q9S	3CTG	2Y6L	3CQT	1L03	3UBH	4J4Z	1VD3	3RFN	3C9X
1GFU	1KGE	1FOB	2X45	2PQ8	2Q3F	2XJJ	1SN4	1R1M	2QK1
4GZM	1E5W	1QZY	3A03	1F42	3VOF	2VN9	2WZM	2HHL	2FD2
4A14	2YW8	3TE6	3Q67	3IHF	1CC2	3H7J	3K01	3V09	1ENC
2EYQ	2YYX	3JUH	4AV4	3V3R	2C30	4JBF	2ZRE	3ECR	1C96
1WNI	2ITE	2VH4	1BRG	2AJ4	1CEC	2Q04	3EYP	4ATL	2IN9
3UF4	2WHG	3JRK	3OG7	3GBG	3W0K	1SHX	1IGR	2RCK	1USW
1OZN	3FGB	3TEY	3ALK	1GBE	1VKZ	1CK4	1GTG	2R2Y	1P46
1E9M	2HEN	3TCO	1FMT	2GF7	1V3J	1TXD	2HV6	3HLZ	2NT2
1QX1	2OXO	3D8P	1HT6	1T8K	1L67	3OLY	3BN6	1VFO	3CKL
3I11	3PKV	3HGB	3BFV	3GFV	2HEL	3UI4	3QUP	2VN1	3LQH
1B0M	1XDF	4FC9	1R7O	1BM8	3VM7	1AU9	1PK5	2XYC	1RTQ

2R7Q	3PU5	2OTD	1JFX	3H8N	1HDI	1HEM	3PZ7	1914	4G0O
1SUU	1SYK	1W8O	4F3P	2QGN	1Z70	3BW8	3D3M	3RAY	3TPD
4IDV	1HZF	2GPJ	1LYH	1PU7	3HJE	2D0K	3BZB	1QS9	1WKO
1OT1	3FCZ	2IQ1	1IOQ	1RUW	1CO9	1DIX	1X0T	3BJ5	3Q52
1FKX	3VAW	2J0L	3EGE	4GIO	4FB2	1EH6	4A4X	1FAS	3OS7
4HTE	2OZU	2R77	1GZR	1CEA	2QFF	1RQC	2WEF	3PWB	3IBZ
3QK1	2QOD	1XEO	2Q1F	145L	3OJ0	1M4B	2FI9	3MY0	1YW5
3DLV	2D1S	3N8L	2QZJ	1R26	3VJ9	1PG4	3H4M	3ID1	4GGC
1N1I	1HQT	1AS0	3GOH	1KW2	3ZR8	2Y8K	2F0H	4IXF	112L
157L	2VVG	1OXS	1B8R	1KCK	1EL1	1JCF	3ONK	4DZ1	1RBS
3NR5	3UYX	4FZ4	3B0T	3BQQ	2E4T	3AMS	104L	3QWX	2FL7
1M55	2ZUH	2BX5	1TTO	2XH6	1XBI	3K0B	2PVJ	3QF2	4DFA
1PIG	1ZSL	2CMW	1Q9E	3O3J	3AHY	1R5B	2WSK	2VUZ	3UK0
1M2K	1YQO	4H4A	2O3R	3LYR	3EOI	3TO8	3ITP	2XH3	1L1N
3CIA	3BOR	2A3R	2QT6	1L2P	3VF1	1R1H	2P9W	1YW9	2YXY
1CDY	3BVM	4GU5	3MBT	2Y8F	240L	2QJ6	1VJ1	3CSS	134L
3FIM	4IAX	1U5G	3H5E	1PTY	2WN4	2W2J	2FJ8	2WLW	3ADY
1A54	1CUX	2D2S	4EOA	2XUT	2BQD	3FJH	3G6M	1PGV	3GNE
1KDJ	3ATV	3F8V	3RP9	3MHB	1VJK	3F3B	2OUG	3TEF	3O5X
3SB4	3CH3	1UUH	2QXI	2Y6H	1UDX	3FH2	1L64	3TSH	1FN5
4DIU	3FWA	2IGP	3DKE	3QPK	2BON	4EUY	3ME5	1C52	1Z9X
3ORM	144L	2CDO	2MEG	1GTK	3ZYQ	1G83	1HJS	1R75	4H7W
114L	2CBO	2D7I	2CFO	2PVU	2VDF	2IU1	3F6R	2H5P	3KSN
4EXL	3S9B	1AFC	1CU2	3NIT	2VAD	1DS3	1M2X	1CQQ	3OO8
1IU9	1XR6	1PBV	3V10	1LHK	1MTI	4JSA	3R9K	1CXL	3H9M
1AZ1	2FR3	1STB	4AJV	3RL4	1NNT	3DFJ	2PLU	2IF2	1KLT
3VJD	1BSD	1LZV	3FFO	3Q5Z	3UFB	1XTQ	1LKF	2FCS	2FMU
4IDC	1AMJ	2D02	3C1R	3CLW	3IKB	1WAO	1GB8	2WZ9	3HKY
2XO8	3ZXJ	1U09	2ZJJ	1ZYN	4FUS	3TSG	3GCZ	3TUA	4DQA
1SI7	3HH8	2Z0Q	1X1I	3IWO	2D4D	1B6A	2H5E	2VJW	1F24
4J33	1AJO	2WY4	3DDK	1RMG	4JC8	1JRY	1L6Z	1W40	3H5K
2V6J	1SNM	1VZX	3AKM	3M4V	1C9N	1OC7	3W28	3M7G	1F5N
3TQL	2B8X	2C1W	1K1S	1B2O	3N0E	4H7B	3NYC	3F5V	1YI6
1QTC	2F2Q	4AHK	3LMG	3PLS	2EVB	4J5H	3DFI	4DX2	3PVC
1AGY	3F44	4I0O	3RKG	3V3D	3RK6	1IDK	2EW0	1B7R	4GK9
1ESW	4HXY	3SNV	2HXP	4FLO	3LW0	2RI0	1I6S	2I5T	4AIV
1TAD	3BOD	3P3O	1I2A	1BFE	2YG9	2AT3	1W2P	1OMY	3NXU
2YKD	4AHM	1PC5	2W62	1VKW	1CCU	2HL7	3CKW	1FVG	1L8Q
4F5B	3Q6D	1GNL	4H5G	3MNI	2Y98	2Q5E	1HGW	2WTS	5EAS
3TQE	4I53	3Q6V	1W9I	1N67	2Y8D	3R5L	3H93	3TOI	3MZ5
2A14	2PO4	1T2H	2J21	3C38	1MH7	3U6J	2XTP	1FFE	3S6C
3O6P	4FFG	2Y1V	1DNP	3GVZ	3TKB	1WDU	2VXR	3QC2	4F10
3KX4	2PFT	3HVM	3K1Z	2QSA	3PIV	1HV0	2QPW	1OXD	4E72
1RXT	1R86	4HTW	3PHH	3DSM	4GV1	1MBA	3AXD	3I76	3TP6
2FCL	3KB9	3CMZ	4HWC	3MWF	4F78	2EXZ	2O0B	1JK7	3FRR
1Q1U	1MYI	1HW6	1ORC	1J9E	1PJ9	1N5N	3KCS	3KH5	3RFA
4JC0	4HBS	3MXX	1WY7	3BA7	3B44	1ZT3	3N7L	1E62	2NWD
1TDY	1DJB	4A05	2JKM	1NA3	1DDW	1N82	3ESU	2AIQ	4F42
1F1Z	1R3D	3RSB	2Z2N	3JUX	2O68	1IKT	3OA3	3DQ7	1LR6
1Z5V	4L1N	1B7M	3DAN	1L81	2X1Q	2SPO	4H0A	1V2W	1CXV
1NTG	1HCZ	1L42	2W69	3TTN	1SQ9	1XEZ	1DYG	3HKS	1UHK
6PAZ	3M3G	2R4B	3TAW	3CJM	4E2W	1XTK	3SLH	2GJP	2JIL
3QT4	3P28	1GUI	2IHL	1W1D	2PVX	3A0J	4A2B	2HYK	4GLP
3DU6	1LFO	1KS3	3UXU	1FUE	3BL5	3HAK	4FDY	2CM1	2HEE
1BFF	1Q1Y	2R31	1KFR	2ZZI	3QD7	3GOE	4E2P	3FY1	3O16
1YD2	3GVO	2P8R	1SEN	1SQG	3MAM	1HEA	2YYZ	8MSI	1W0N
4AA9	3OAN	3MJV	1VJS	2WFT	2PE8	2B3H	2XJ4	2YD0	2L78
4E4P	1J8Y	3QBE	1Z9T	2DYL	2UWA	1KBB	1XK9	1TUA	4FI5
1Q9C	1SIU	1ARB	4FCF	1THO	2EC5	3LL1	3ZYO	1F2Q	1CNG
1ZBF	4E9L	3DXL	3GG7	1P9Q	3DGT	4FBE	1K6K	1Q9I	3AWM

2V26	3E6J	2WF1	4A4A	4HVD	3NJT	1CZK	1G6C	1BYQ	1OK0
1KXW	3T66	1PKF	3CEC	2MEA	1B9W	241L	4DY3	3NXO	3QWW
4AZW	4B7O	4HEF	3W4P	1P2F	258L	3HMI	3INA	2R4O	1TH0
2WMH	1VJW	2QPS	2CXA	2RHP	1XDN	3ZXL	4ESM	3IOS	2VWD
1BCY	3GLJ	3NXP	1FVI	2BUE	4AD8	3PH2	2YD8	1ESI	3MEW
3P5U	3M66	1DSO	1W32	1OQQ	2MBR	3FJZ	3JVL	2Q0I	4KDS
3GF9	3N5A	1Q03	1U84	1CNE	1T2J	2P8E	4JOM	1NJ4	1A0I
3ZSN	3I4L	3V0C	1QUS	2WLP	2Z4T	2QXZ	2ZQ8	2FX5	3CIG
1JV0	1M20	2PNM	1JO8	2XR9	3PRY	3DXF	2Y88	3EQ5	3O12
2IQY	1FRM	3MKT	2BG1	3C5M	3FVO	3UN5	2J20	2Z2F	3JZY
3Q6L	1BG2	4GBM	4DY5	1MX6	3HZ7	2DUO	2F8Q	2B7A	2FXU
4HZ8	1G3O	2DPK	4JJA	195L	1B5Y	4FH1	1AOL	2BW8	1B7K
2P2Y	3MVR	4DGJ	1MMA	4BKJ	2V3G	2RH8	3I48	2FCR	2FGT
2PVT	3M6Z	4I5J	4F4C	3IIV	3M0U	3L01	2RCI	2FGZ	4FFH
2Z80	4EJI	3DTM	2ITV	4GWE	2GKR	4GRZ	1B7P	1BQC	2FRG
2A8E	4F40	1NZ2	1VKU	4KQC	1L26	3S01	4DIM	1O70	3SE0
4IU8	2HBW	3D33	1EU8	4KU4	3NXF	1ENI	1RI5	3O6O	1BIR
2APB	3S5F	2FLH	1EBT	1GAZ	2V7F	3K0X	2B4A	2ECE	2PET
1GIU	1TZZ	1QST	4DW4	3BAD	4EQ3	4F1P	4PBG	1MLL	3D5L
3DK3	1I76	3JSN	3CER	1VL5	4K2K	4AEK	3AQ8	4H07	1RP5
1BAG	2RC4	2RDF	3QT9	2FFZ	2IYF	2NZI	1Y9L	1S6R	2FOK
1O0E	1CJW	1M4K	1FKQ	2QTY	1OJ1	4EOD	2G7C	2R4Q	1UOY
3OG3	3L9S	3U62	2Q9E	2WB7	2PTU	4DBX	4FQT	4FHO	3N5K
1C3F	1G0Q	2Z9Z	2QUZ	3L1Y	4JZU	3GUI	3HBW	3FYQ	3B1X
2G5R	3S1D	2GX0	4DPZ	4J1M	2D48	3TC8	1KEH	1ZD8	3T9M
4KDX	1YOB	1CPM	1NPC	4IMV	3KY9	2X9K	1QIL	4ISC	2Y9F
1NPI	1JWV	2IPI	1HK3	1BB4	3RWX	1ODV	2ZE9	2E3U	3I6I
3ZX7	3C7Y	4ACR	2W74	2WOE	1OA3	3VO3	1LQY	2BQM	1PHT
1EZ3	3D60	4F8M	1HHL	2DRQ	3RJN	3GP7	4AN3	1F7C	3V5C
1LHM	4A99	1ZM8	2YLF	1V0L	3CZC	2HTA	3SFZ	1Z6T	2Y2F
1FIO	1U9A	1QTH	1HEN	1IOS	3NRY	4JBJ	4I96	2RBT	1NPS
3RWL	3EDU	1J7A	2DZX	1GIQ	4A2P	4DIF	1XSZ	4GVV	1VEM
2HK5	2RC7	3DIV	1ZVG	1MQ9	1ZOV	3SEW	1P77	1NI3	1ZI6
3ZON	4BHC	3T16	2PDU	4ALZ	4IUN	2ACG	1JC1	2PD9	3OUE
4ARN	1NQZ	3S83	2XTQ	2ZQO	3FVV	2FJI	3M70	3RY4	2OK5
1XLR	1WMK	1EUG	4DU2	1L49	1GVY	2W98	4K8V	2B4L	2F0F
3GT5	3HL1	3H2G	1FRN	2XZG	2R44	3PP0	4JG3	3QVE	3DTQ
4B53	1VAH	1KM8	4JNF	3HRL	2PQI	2CHF	1NH6	1ZZK	1DD6
2O6A	3GYC	3V39	1E69	3L4C	1MKY	1YQE	2ZJ7	1MYH	4FOC
2XA4	2E4Q	4EO7	1L54	4IRX	3SFT	2EC8	3OKZ	3E2V	1UNQ
3GDE	2Y1Q	2NUK	3PE6	3UMN	3ORK	1UIC	3FG2	1PQO	2CYX
3FCE	3SWG	3JUU	3FJK	1KQJ	3PUR	3V34	3TLP	1H8N	2DE2
1M8T	3TVZ	1LOY	4GLK	1OBM	2QK4	1IA6	1SUC	3QL6	2AG4
2Y3T	3UZU	4DMY	3LJX	4KQ9	3DKC	3A6T	1G9O	3P4L	1GFJ
1TG7	2RDK	2DRO	3ESX	3NZ3	1NIS	3TNY	2G3I	133L	3ZVL
3BRS	2GDJ	2OCH	3FJI	2ICC	1H6X	1UHA	3SK7	2AIU	3VC0
3ZM6	4IVA	1BD8	2AGK	2Y8U	3CNJ	3PYA	2YE1	2HQ6	3TOP
1Z3X	3LKM	2J8A	2O36	1YO8	1M6M	2E84	4I3N	3B1V	4JBU
2HIS	3IDW	2H5F	3ZXY	1MLG	3URO	4HD4	4HXC	3A35	3ULT
3OWV	3LHE	1ZIX	3MD2	1CNV	1VK3	3SV8	3R0X	3AU7	159L
1K5C	1KP6	3NSY	2XA5	3HH6	3IK2	3R4V	1JAE	4IE5	2VKR
2H1A	2HRG	4DN0	1JKB	4AKL	4E9S	1Q1Q	2GUF	1PQJ	3P0C
3E3V	4ANV	4DCA	1GB0	3R63	4AY7	4FUH	2HPJ	3LZ3	4G3G
2XT3	3FB2	2VTF	3RZF	2UYX	1VF8	1TFD	2PL6	4FQG	3QCN
3MHZ	2EKC	1MID	4E36	2XMX	3QON	2B8U	3OWQ	2FZU	2VKI
2MGG	1GF0	1EMR	3KA2	1D4Z	4JDF	1NGD	1UUQ	4DCG	1R2E
1GBZ	3RGA	2QKQ	4HW3	4FXQ	2QOO	3GEB	1ION	1HER	3GQS
2PKO	1KDO	2IAP	4HTR	1RJ6	3RH0	2XED	4BA6	1PV5	2NLY
7A3H	206L	3NPH	1KMV	3M7M	4FMW	1XK0	3TQW	3Q1H	2FH1
2ZXR	1WU3	1YMR	1PPI	1AD6	2W4O	1QTZ	4FOZ	1IU1	3D1P

2ZF9	3U7E	1ZSW	4IDH	1CNJ	2FIY	2YHX	1ZZW	2PEF	1VI5
4FC8	2XET	3MYX	2F9F	1B2Z	2H4V	3APA	4EIU	1T3S	1X89
3UWI	1ZND	2VK3	3WB8	41BI	2HLY	2DWR	2GIV	3MY6	2IJX
2AJA	2XHA	4AIO	2WB9	4EGD	3BQF	1SFS	3ABA	2X7G	2VB1
2VHK	2DCB	1VCD	1JJF	4EFO	3USP	1UKT	3NBM	1LUE	2C9R
2HAU	3NRS	3M5Q	1IAP	3NGO	1JGJ	3KWU	1JC9	4IOU	1G9G
2VVD	2C8A	1PUJ	1HV1	1L77	3CW4	2W20	2NLK	2PGX	1P2E
3R4R	3A77	3NUL	3DJE	2QQJ	3C8R	1FDR	2BQE	2JCB	1IOT
1F1F	2XHC	1RBT	2HAI	1F9U	1NAR	1W7B	4A84	3LCD	2PVE
2BM1	3JWQ	3MBR	4HYQ	2QNG	3F9E	2ZXQ	3T7A	3B5E	4HMJ
3H7I	2E22	3MTX	2Z1O	1W8U	3NX6	3IW4	1RN1	3OQ0	2WHJ
3FM4	2YPH	1SRA	2ZJ6	3JXG	1OR3	1IUD	2XJL	1CCZ	2XJP
3LE0	3HOE	2HWA	3AXF	4HTY	3S93	1T5J	1L20	2PL3	4F1O
3VN3	2JGA	1IAE	1BEZ	3F3Q	3KS3	3DC4	4F0W	1QHL	3D6S
3S8J	3HAP	2FTA	4L05	1CRH	2O5P	3O0F	1J7B	2VQC	174L
1BYG	3AIE	2Q2O	1LYF	2NLR	3EVR	3LUP	3HKM	1L8F	1NGC
1WQP	4DOU	1NPM	1QYI	3CHJ	3I7A	2P4H	1UIP	1MMG	3FLB
3R9R	1HX0	2HK6	4B6W	2OL6	1YNM	4BDX	1H03	3PUU	2OHB
4K0R	3NRO	1B89	3GO5	3FSA	4LOF	3CEB	3O2U	3V9E	2CLT
2PDP	2WMI	1BSG	2QU8	3QCW	1W7O	3GP3	3O6A	1KEX	1BP1
2PB7	2AE0	1B1Y	3SR1	1G81	3UMV	4K2L	2O3G	4GFJ	2FD3
3S9J	1UWC	2MCM	2HCG	1FGO	3UCI	2OJH	5PEP	3JTN	2GMK
3JTP	1R69	3SUV	3VNR	2I4I	3BDU	1CV5	1D2Y	4K37	2AMG
1ES1	3VMN	2XRI	1L36	1CNU	2R2A	3SIT	3BEE	3V6A	4HBW
4AS8	4KK4	3A7L	3LFG	1H04	3SWA	3MRY	1GBJ	4I90	3JRP
4DB6	1T91	1J1T	1BFP	1FQG	1DYF	1G4W	1YCK	2PE4	4FH0
4EIC	1M7B	4FQ8	4IUQ	3OV6	1B7N	3IQS	1AUE	249L	1A6G
1GQE	1WXR	3MK9	2E87	3I6D	2O3H	2UZC	1ITC	1XTI	3BW6
16VP	1Y8A	1NGH	3PU6	2GQT	2HJV	3PXB	2UY2	4KNC	1N8I
3A3C	3OCT	3O6T	2R5F	1QMN	4JIU	3MPM	1JDC	3DEB	3QQR
3VVN	2Q9X	2WJY	2XI9	1H9Q	2B5N	1LHI	2UXT	2ZQ5	3GEA
1B68	3Q2E	1AGJ	2ESQ	2W6K	2D5B	2VPN	3FUS	1JMM	3K63
1K03	4A3Z	1NCX	4GIB	1GE0	3GKZ	1OD3	3VLX	2FR1	2B0P
1B5W	1Q8I	1ATU	2VYY	3IL0	4EZJ	2QDL	1AB5	4AHF	1BKM
2GIL	1WB4	3PYW	4G8K	1IN8	1HIP	4FLQ	3FVZ	1KPG	2G98
1HOW	2DEB	3VCO	1GDW	1ZL0	2O8S	3DT5	3OEP	3LWT	141L
3UK8	3I77	1G4R	3ZQ7	1NMN	2G78	2O6K	2V77	1X23	1GXM
3L15	3ASH	4IM7	3QCM	2WBK	1H76	1CAJ	1ZZY	4DBR	1UA3
3A62	1EOE	4GVW	3M7A	2V0S	4I65	3F4T	2ESC	2FN4	4EVW
2ONU	2V5U	4HJF	1P9Y	4DGF	3IG5	1QXA	3US6	1J8M	1XO7
4GGA	3IOX	3URD	2TIR	1IT2	2CJZ	119L	3N12	3OE2	3LPP
1OLL	3FWG	1MKU	4E2T	3A38	2WA7	4DFS	2O7K	2SQC	1MNC
4FL3	2XY2	2BPO	1ECL	2ID0	3IXB	8AME	2V2U	1LN1	3KKW
1HIB	3D4E	1S8E	4F1M	1JBK	4J3D	3K7I	2C3Z	1A0P	1LSN
1JWQ	2RH1	3SK6	2RES	3RIO	3GF6	2ZQV	3URZ	2GVX	1IQQ
1UGI	1G0P	1CS8	1KXH	2XTL	2GBA	1N97	4A2Y	3IB4	2PGG
1RDS	3FGM	2DZS	3NO2	3H3E	2YJ3	3JYB	3QE7	2BIR	3UYW
3DSZ	1IH7	3GRC	2HW9	1VA5	1F0B	2IHR	2BF5	4FQP	3BFO
1BUE	2FVC	1MI0	3LHI	3E9F	3GVA	1G7N	1ATR	1WCX	3BD9
1LZG	2J6A	1VQR	4G4G	2QGQ	4H6W	3K2T	3SHT	1E2W	2Z0T
1LLT	1TMY	2I1S	3ZV2	3TU8	3KST	1VR8	3IHC	2Q4M	4DWM
3BCY	2E6M	2VQP	2NW0	1GMQ	2YZU	2W2S	3H3D	1FZU	3ETZ
3QFT	1DSE	168L	2V33	1HB6	2OH9	3FXB	1QBA	2ZFU	2R48
1GV2	4IGZ	1WLZ	1KYA	3C8U	4IFA	155C	1ZIN	4I6K	4B0R
3PXA	1Z7L	1PCH	2V14	1QGZ	3MIX	2WCQ	3BVU	3QZ4	4EK1
1OUJ	2QOR	1J09	4F8A	1JQE	1AOW	3B1Y	6CA2	3NEZ	1KXE
2NQ3	2I6V	1LWB	2XKA	2H2Z	3TGA	3CKK	1UKR	4G0H	4F72
3OGN	2R9U	2RI3	3E0S	2VJ2	2R4N	1NG6	1QV1	3IEH	1LIT
2FPO	2F8P	2XXQ	1R19	3UUE	3AWQ	2JG6	1PEX	2VSP	4I3B
4BMA	3ZIU	1IFQ	2EJX	2H3L	1D6Q	1MB3	1PMP	1AXN	1JOS

1K6X	3K29	1VMB	1N4A	3MWU	5CCP	2C1G	3IRP	1YSC	4HLB
2XUF	3FJF	2OUD	3AS2	3ZJK	1NKR	2OEW	2PZU	3VUO	4GL6
3FHR	1MYT	1TIB	1D4D	3SK8	1WK8	160L	2I7F	1FO3	1WZZ
2VZ6	3TRG	2BMF	1KDB	1ZX5	3OLC	1GCP	2H8H	4F8X	1H4A
1PM1	1C2R	3VCF	2WFQ	1B3E	1CU3	3NW7	2F4Q	2DIE	1SJX
1JU2	1HO8	3EE1	2ON8	2P7S	2Y4Z	1H71	3SXS	3SZ6	1B85
2Y8P	1BX4	3ZVR	4KPP	3UVH	4DVY	3JUD	2FZL	3RDD	2QLH
1CSX	1SMM	3SO8	3RC9	3GIA	1BRI	2JLI	3P75	1QL6	3HOP
2YQC	2RKS	8CA2	2YHG	3O0W	4AX0	3OPN	3II7	2HPS	2WVI
3PBT	3AII	3MCY	3RZE	1NAF	3BAG	1CH3	3L6A	3DYJ	3FJW
1B5X	1KSS	3DHN	3DEF	2XTZ	4F7X	3SU0	4K5X	1G0X	2HZ3
2FN8	1R6B	3K1H	1HBK	4B88	1VJD	2EWK	1HQ2	2J4T	2Q3H
2RG8	2WE1	4B8R	1EDG	1QDE	2EF1	4HS9	2A0Z	3W6B	3ZKP
1LAR	1CZA	4EV8	2QJC	1Q1C	2RA1	3FJC	2H4T	1QME	3F8Y
2PNY	3NWO	2PYX	2Q1H	2WM5	3LIF	2IW0	1NZ6	1N76	1HYF
3BHH	2YW2	1BA2	2P5P	1CIY	4AP1	2JIA	1QCX	2C2P	2ZJK
2WTB	3H79	3B9P	2V72	3V8E	4FKE	3CWG	255L	1SGV	2YHV
1JZB	2X9H	2QP4	3UN6	2O8L	1BTI	2KZZ	2ICY	1FLJ	3VFI
3MB4	1L45	3HN4	2PBL	2C4P	122L	2YIH	2TPS	1N3X	1F6W
2EAE	2Y7O	4FD5	3VOC	2Z63	2PTV	2H72	4AVA	2BSP	2G09
3IR8	1XWL	3OH8	2O1W	2I0Y	1A2A	1EAZ	3UGF	2CXX	3ESY
1XO1	256L	4F5U	3BPT	1QHU	1ZOA	2ABL	3QR5	1Z57	1BY2
1PZ8	3S25	3TSV	4K9R	4IUP	1V0A	2W9M	2QHP	2WJ5	3S44
1HEC	1T2U	2CGH	3FLG	2F68	1QTW	2WZB	4JNC	1NZK	1E4V
2VM1	2X2O	3H5L	1EUF	2FY2	2PKW	2VC8	3GPK	1L07	1USH
4FET	2FDN	2SHP	2DW4	3M6C	1SAU	3ARX	2OFZ	1M1Q	2V73
3B5L	1NO1	1Z2U	2Y7S	1TJ6	1HEK	1XFJ	3Q5I	1CY5	2ZNB
2HWW	3JYR	3V3L	2A1X	3DVD	1PTF	3PT9	2NSM	1OIW	1G0M
2XMP	1UXZ	3CUI	1MRG	3IIG	2PWX	1SR8	2X4M	1T85	3KHQ
1B1X	1FEP	3VLG	2NT4	3KLQ	2PC1	2GKO	3ETO	1IRX	3D09
1MRJ	1SAT	3REG	2XZV	1WYK	3FMZ	1SQY	2QYK	1SH7	1TTJ
3W06	1V2O	3O7P	3B21	2YV4	2OOG	1ICN	1FT5	2JHM	2Z5G
3II1	2XBK	1EOD	2PRB	4AMS	2I5V	2XVM	3QEE	1L14	2V5C
3L5K	4GZL	3PZ8	4HTH	3BC9	3L8E	3ODZ	4EMQ	4EYC	1HU8
3QLE	3RG9	2AHF	2HDF	3TBJ	1WOL	1EGU	3GUE	2GRJ	4KG7
2YPS	4EL4	3OP8	3QIA	3PE0	3SHL	2AFT	3URG	1TJL	3L11
1L21	4E2Y	3F2E	1IZD	1KOE	1QOK	4A4Z	1XR2	3RA6	3FQ6
2Z15	2FDQ	3H2I	1WBA	3SAO	178L	2II6	1ATT	1CPQ	3BUS
1IIU	1WL8	1JN3	1SKJ	1XMD	165L	2AGS	1AF0	4JGT	2R9F
1XMT	4DC3	3Q8E	3B5P	3S5E	3VL9	2H6Z	2WBN	2BV5	1Q5D
4I3F	1C7K	4FK7	1GCY	3K2D	3FK1	1T2P	1YXS	3EAZ	3EDO
3ZH3	3TVV	4F9T	2ZQS	2EYJ	2VGE	2WH5	2PGF	3OK0	3UMR
4GD5	2VQD	1FW8	1TA0	4A6P	131L	3QYZ	1QUA	2BWK	1Q4N
1ES3	2IWV	1LSL	1K2Y	4FDQ	1JFH	1URT	1MR3	3M0O	3BT8
4JQR	2Z81	3FB4	4L6E	1GOZ	3QKY	4DMV	3Q9O	2FW5	3NBX
3PXJ	3R9F	3T6P	1E7U	1Q20	1AH4	2EYF	2H57	2JAY	4AHL
3RGZ	2BKG	3FRZ	1HXI	3AHN	2CY3	3NQT	2QGZ	2X9O	3OZZ
2OUV	4AWD	2BGV	2AC1	3TYQ	2ACT	1WDP	3B2G	3LCU	3A9G
1T9Q	3FQG	1EAN	1DCC	3KM5	3FGQ	253L	1PCA	1RW9	4HD5
3VSS	1KB3	3RSW	4AUK	3JZ9	1JPA	3PB3	3LOV	1QUK	3H4N
3SK5	1Q79	3F88	1ZIW	4GC3	1CDZ	3B79	3AQY	4AQ4	1OFD
1J3A	3QA9	3LX6	2WE0	4H2C	3OJ1	1MNJ	3ATR	4FOS	3E11
3UYN	1XTF	3A72	3HL9	3VU0	2AIA	1UOZ	1XKN	2OY8	2DJH
2ZXJ	3NIX	1T4O	3AA5	1L5J	1HYQ	2O3S	4IPB	3BWW	3SLS
2Z8Z	3OOI	2AAE	3A0S	3JW3	3L2M	2RIN	1L6P	3RVO	3CBZ
1M4A	3TNX	2Z4S	3V0S	4A67	3HJK	2XWI	3AL2	1T2O	3GNL
4H1G	2I27	1IU5	1OCB	3O26	1JFM	3P2A	1DI5	2QLU	1TQ4
1HPL	4EYU	1XWT	3AFG	3CW0	3TX1	4E93	3ZYN	3W30	2PSD
3R5Q	3LQ9	4AFD	3M4F	1EQJ	3HMC	3PT5	1KSW	2UYR	3FYS
1TAY	1BEA	1BEQ	2JC4	1Y1P	2A3M	3GH3	3LIG	1WNH	1FRL

2RDV	1KGF	4K6D	4AW0	3ELG	3GON	1CRB	3HY0	2H1Y	3APO
3IV0	3MZY	1MGT	3B0H	3S8S	1YD4	1OXX	4E76	2MEC	3D59
1VFF	2LBP	3BUJ	1V3I	2Y38	4FL2	2PC8	3P97	3A7N	2HB5
1JUG	2I74	3DKR	2FAF	3OOU	1NGB	3ZVS	1EYC	4AOS	1D5A
2QO6	1CUZ	1DTI	1IZQ	2W8D	3OSO	1ZZM	3K50	2ODH	3PS9
1Y0K	1QYR	1DF7	2CZW	1M2J	4AZC	3VOR	3IIJ	3CNY	1QHO
1ES5	4GNQ	2GS5	4AMF	1GAO	3ZMM	4GU2	1OI1	4KVP	2PQX
1FPZ	1KQ6	2HKO	3V3N	3ODR	3MZ4	1WKU	1FQE	3BSQ	3D2L
2YPU	2XEE	3RPM	3CBQ	1GIT	2ZBI	3CFZ	1GSW	1GHL	1MMQ
1DL2	2V3V	4A1Y	2C8E	1N3Y	3TW5	2PHC	4EID	1PUB	3CB9
1DD5	1TCA	3LOD	3GU4	1I5I	3DW0	3IEU	2BTL	3AFH	1FND
3S5Q	1CNH	1TEN	3DMN	1LOX	2CWY	1Z06	2QGU	1C8X	2F0Q
3A4W	3FRH	1XW4	3KIV	3VHQ	1ELV	3UJK	4GXR	2H5Z	2V3U
2MEH	2IUL	2XEV	3JWH	3ODX	1IRK	2G29	3SG4	3EOD	3UW0
1YHW	3OFE	2PDB	1OEN	1B24	1CRM	2FDX	1NB9	4E19	2IIP
128L	1RQW	2P5I	3O49	2RJB	2AIF	1TLY	2O37	3LRA	3KPK
3BSH	2IQJ	3NK9	1BEM	1BHE	2X6T	3QXF	3ICO	3ZRH	2FM9
3U1L	1L1L	1LMH	1HMY	1IIB	1W66	2FWH	3DIT	3BCN	3IUZ
3FEO	3SK9	3FAC	2GAI	1CH0	2V3Q	3B9E	3EGB	2R2J	3CHM
4GVI	3AS5	3TH5	3MSV	1LSM	2KFN	3UTO	1I1O	3ATG	1WOJ
2W9H	1DYM	1PQM	3ONJ	2H6O	1RDB	1WVN	3CUZ	2YLR	2PSO
4EJ6	1LS6	3C7Z	3SI2	3HAV	4ANS	2HDV	3NZ0	192L	2XZI
1H6Y	3MSI	3U4V	1RNF	1FLY	2ZWU	2PXX	3GC7	3VSF	2WEI
1CH1	1GE4	3VN9	1SGW	2AR0	1AKE	3A7R	1CVD	2WEE	3F95
1GV8	3ERB	2O85	1CVA	3B89	2H76	1MJS	1JF8	4H24	1RH9
1CEX	4KEF	1XNB	1CIG	2VWR	3I7J	2D0F	3NCX	3OVO	2BPS
1HDO	3NPL	4IIP	1C66	1QQL	3V8U	4PAZ	4IH9	2H0K	1IWD
3NQ8	3QOJ	2FQ3	3VHU	3I1A	2MEB	1CVH	3PD7	2R39	1VKQ
3ESL	4AAJ	1Q4E	2GGT	2HKE	3CE7	2VRI	2I0W	1C8J	1CJ7
2WHH	4DTY	1BUD	3MCS	2E4L	3MDZ	4EPZ	3CGN	1TOV	3ZPP
1L55	3T02	3N2T	4DKX	3HR8	3C1X	2H18	3MEM	2ES7	3RLH
3V70	3TIH	4IB4	2UCZ	1LVA	3QR6	3C0L	2XES	3B0O	3TLK
3VHE	1GFV	1LOW	149L	2DUK	1WNA	3POM	3AHX	4DGK	1CDU
3KIJ	3DQW	2C60	1P99	1FYS	2Z37	1FTN	3GM8	3FJD	2JAK
3GPG	4DDU	2AME	3ON2	2GW2	1VJZ	3PGV	4JER	1LSG	2BX6
3QUF	4DQK	2BLN	3CXU	3V7N	1RU4	4IC9	2Z22	3B1Z	4DZD
2IRU	1JX6	4JGL	3KQ0	4FCA	1A62	3FC7	1DKI	1MOL	3N7M
3TXA	3ORJ	1CV7	1XAW	3FTD	3SLJ	2VFR	1YPN	4JCP	2B7J
3A5E	3M4H	2BZG	3QHB	1OA4	4GQO	2WD0	3B40	3UMJ	1QN2
3AKB	3TKI	3M95	1LRZ	1SDI	4HQF	3VM5	1W3E	2ODL	4GFI
1ZHM	1N7O	3NX0	2DYK	1BK1	3DHP	1Z1N	3W9A	1L99	1H45
2YJL	3VGP	2PD7	3CDV	2C9C	1GZJ	1HFX	3CJF	3EUG	1YX9
3NY2	2B45	3F7J	1K0E	3I2D	1CGY	2CJ7	1LFP	3K7B	4GER
3V3J	1UCH	3BRN	2DRW	3DLC	3GLE	3PY7	1HNN	1LLP	3E6Z
4GT6	4FO1	3EXN	1XA1	2Y2X	1KFD	1FD2	4AS7	2OVA	1RCY
4ABN	4HV4	2J7K	3H8M	4FOO	2QIM	4KAI	2YV0	1LS9	2YKH
2NMS	3VNO	1DYC	4DNS	1E2V	2O1A	1QI4	3HD1	3A21	2B8E
1EK8	3GYP	1ELK	3TFG	2WBX	1U06	3FIA	1JF2	3JUY	3ZH8
1YH2	4HJC	1RTP	2QQU	2QCP	2ADM	1RTZ	1YKS	4HJI	1XC1
3O8T	3UQA	1L7P	4H2K	1EHB	2XR6	4GDN	1DY5	1YAQ	1S21
1XR5	2BO1	3VHV	3GG6	2V76	3BUV	1Z8Q	2P4F	2R0O	3US7
5PAL	2HUC	2WDC	3V3I	2VU4	4EQ8	2Z16	1TZV	2OEG	3ZU3
1C4W	1ZPU	2AFY	1XMJ	1TIE	1JI1	2BSA	1IR8	1NO7	3P5P
4EPC	1B59	1AF7	2QHS	2NS6	3GQO	2HS8	1MHQ	1U9R	1PT6
2E8B	1M48	3FEW	3RSK	3LYW	3U2D	3M7O	2CM5	1U1J	2ZAY
2HEF	2P6O	3FQL	3QFS	3ZTF	1Q74	1HD2	2GPS	1MH3	1TUX
1J7C	1AM5	2DG9	3SJI	3TGZ	3JZN	3E13	3C04	2A0J	4FEH
3BMV	2XST	3Q5E	2H79	4RXN	3FJJ	3P24	2H6V	1UGA	1BX1
2YHB	3FUQ	2BBO	3UMT	3LFO	1R8I	3FPX	3R5E	1CU5	1PK3
2Y09	3KWI	1XCH	1EP8	2J89	2AAM	3MH1	4JM1	2OBD	1YT4

3O8Y	2W9S	5PTD	1S0L	3LI4	1EJ0	3SKQ	1V2B	2CIW	3ZFC
1ISE	3W3E	3PPQ	3DUE	1WKA	1JV6	3TA9	3DGI	2X5O	3LMI
4KM5	3BKR	1Q6D	1KEB	1N4W	1MS5	6CGT	3K0Y	1NM9	4IXG
3MB3	4BQB	1Z0N	1UH9	3FPY	2QW4	3K3O	3PY9	3AG0	1OGO
2J4D	1KWM	3UN3	1L52	1H6M	3PG1	3ODW	3A7U	1VK1	2Q3W
1EWF	1SS9	2QY9	3IUT	2RA2	4FE9	3RLB	3CTK	1Z8F	1XHB
1XEU	3IK3	1RMO	1MGY	3G1N	3BF8	3PPG	1HJJ	3RSN	3ILO
1GYU	1ZG6	2XRU	1D4O	1MK0	2SN3	2OYB	1RDR	3C65	1UOL
1CH2	3FYM	3UP2	2PLW	2ZA9	3FS7	2O2X	2YOL	1D1Q	1KU3
1YS1	3IBT	3CV9	2G6D	146L	3BZ5	1VYR	2NXP	4AXK	2OP6
1LFG	2CLD	1YFQ	1MBQ	3EOP	2VX5	2ORE	2XQO	3R4L	2BEM
3QTM	3GCE	3EIG	1QE3	1YAM	3CMI	3GS6	1GIL	2F3L	3APQ
1L01	2AGC	4JMW	1J48	4JND	2X8F	2AH5	1S2X	2QY7	3MYN
1WC2	4DNL	1CF5	3PXH	2O1M	3SP1	1I0V	2VXP	2B9L	4GRA
4I9D	1NL5	3KX5	3LAX	1KL7	1KGG	2OFR	1A40	2NTE	3DK7
4FWZ	4GI8	1LTZ	1TLA	1AEP	3ZY3	4IBN	1VH7	2C43	2JA4
3UD6	3MR0	1ZUR	1XVL	3CX2	1GMY	1Q6E	1CAI	3EC5	3N26
1DMR	1QFZ	2JLR	1KAA	2PPN	3TES	3N0D	2HWM	2QVG	1XZL
4FYJ	1IJQ	4GND	2IWC	1TA8	3F66	2YL9	3OCJ	3IPL	4FYE
1B0L	2WFP	3PTW	2GQX	1YHH	3HT0	2BGI	3RJV	2XFR	1B2R
4EXZ	1H44	4G54	3FTQ	2X8A	1EVS	4ATS	2W5R	2E7V	3F7Q
3PXN	2BIP	3ON1	2WEW	1CB8	1H7C	1GSX	3AT7	3H10	3HAL
1GUU	1BLI	2WPW	2FHP	2WT0	3VZB	1MNK	2YV6	1FRQ	3BX8
1GKK	2VGQ	2JHJ	1R7S	4ETL	3QG1	1XKR	2OG5	1FMX	1EFD
2CDP	3QN2	1D6A	2QOK	3DOB	2B3F	1PML	1QQK	2GRV	3P86
1H3L	2FEZ	2W7Q	2RKV	4BBQ	2PDI	4KFU	1OTD	2VOB	3PVE
3EJN	3U74	3QBN	1SMI	221L	2DY1	3F5A	2IN0	2H0M	1WAR
3C8F	4BBF	4K7D	3AMM	3P84	3MHA	3T8Y	8TLN	1CUG	2NWO
2GRY	3FWX	4ES8	4K0C	3SQC	2Q9H	1LST	3GBW	1K0F	4B2Z
1DX4	1B0B	2APF	2YOY	1I5N	4AEF	1W8Z	2BIK	7CCP	1MKP
1PQ4	2XWK	1OIS	1JQG	3HIH	3ILF	4FXI	3QI8	4BGJ	2YHC
1OGQ	1IGO	2HEJ	3FF4	2IQC	1S1P	1KT1	2AK3	4JHY	3IBD
3EAC	2O0Q	1Y6W	1FZQ	2BQH	1XPH	3NKQ	3BIQ	2ZD2	1ZI9
4B29	3G87	1SP3	1RU3	1TD6	3S7R	1QI5	3E0C	1UX8	3NSZ
2I6X	1STA	2VTV	4FD2	4BCY	3LYI	1B2V	3MYI	1QOG	4EBY
3LMC	4EZI	2Y35	3MM1	2FUP	3G66	2FCN	3LP5	2NR7	2R49
3GLK	4JPJ	3EK9	2C4X	2GHU	2AEG	4HDJ	1S5J	2F0U	3EA6
3MPL	239L	3ISG	3LNU	3S24	2HE7	3A10	4AUA	2YPJ	2ANP
2X2Y	1F32	2WWE	2HEA	4FCS	1NI2	2DPM	1ZI8	3LM3	2B9R
1MD7	2HSI	4DP1	1JXK	2QK0	1DJX	3CPK	3VHO	3ZUD	2ZGC
2CDT	1JDR	3R2I	3KN8	2O2G	3ETV	4DXZ	4DZT	1XOD	3S69
4J7M	2EHS	3W3U	4E0W	3PNZ	1CH9	4IU6	2YYS	2GIQ	3GIR
2YHA	2XLY	1ZYT	3RLO	2RF5	2IOY	1IZ6	3LLP	1CUJ	3F8K
2AC2	1N6R	2IQX	3EUN	2FY7	3QHO	3OTG	2P9T	1T1I	3TT9
3ACP	3N3I	3L9P	2QV3	2EJ1	1ZAG	3LLR	1KQZ	4DNP	2FAU
3SLU	1ZRH	1A2Q	2A0N	3CFX	3DTC	3GIW	3OWC	1EQ5	1OG4
2Q6P	2RDQ	2W79	1XU8	2H1G	1QS5	1QKR	3NQP	2Q9J	2BKE
2W47	1POT	2ICJ	3N0G	3L8Q	1U0J	2GHZ	3QZ1	2V6C	3T5O
2BBS	3BIA	1RJQ	3CFO	1TDH	3EWK	3OHW	2HFW	2O42	2R0C
4GF4	2BMM	3RCG	3RD5	3LD7	3MYM	3V3H	2OY5	1RGP	1NQC
3MX6	1QAD	1XZI	2ZOU	3P2M	2A2C	1I6I	1UKC	3T7F	2IAO
2Q52	2PYQ	3T6M	2D0W	1GF6	3QFK	2Z84	1UOC	1H49	1R2G
2W5Q	9AME	3CKH	2NSF	1GYC	1EA8	4IAU	2EYO	1JF4	1DW0
2A8B	1T1E	3TVQ	2X8T	3OEZ	1U04	1SKF	4FSU	3EGN	1SYF
1IZ4	3FLV	1G62	3JUC	3PXD	1FRD	1GZ2	1L19	2CTB	1JQF
1FK5	1H42	1T16	3O00	1H8V	1S8I	1B6V	2IOI	1CCS	1KB0
2ZZR	1KV9	2BRY	2Q5X	3DZO	1CAZ	2QS9	2Y24	3ODS	2WV3
2R4P	4DAW	1T9O	3B0L	2DHO	1M1S	1K59	1LJ1	3MER	2I44
4EMD	2HXS	1YA9	3Q9V	1CMJ	2XXD	4GF2	4ANR	2AYD	1A87
3FIF	3KXW	3BA0	3ESV	1YAO	1BWK	1UZ2	1O8P	2IWN	3GA8

2JLQ	3VXD	1NUI	3PYJ	3D06	2Z1J	3F2G	1O7F	3SWL	3QCP
3OF7	3C9H	1DYB	1H0J	3RUZ	2ZJL	2EVE	9PAP	1QO2	3IJC
1PW3	4E6I	2BSC	3P0H	3QIO	3K0O	1PHK	1W4S	4F53	4FTF
1XRT	4AWE	2PG5	3RON	1GW6	3P9N	1Z3S	1NQ9	1HDE	2W7A
2JAD	2OY7	3ZME	1RWJ	3CDU	2GSJ	4DDD	2XNC	3C7A	2F71
4HHU	4FXC	143L	2NSQ	4GXT	1THM	3LXI	3T87	1NTP	1OLQ
1OSE	3BJ9	1PBK	3B2F	2NX2	1LUZ	2JIN	2QNO	1ZAP	3H8S
1TQO	1VBU	3IIP	2X1D	1KVB	3PIL	4A87	1OLM	2C8G	3ZK6
3TUT	2ZQ7	1D3C	4HD1	3VQT	3I1E	3B8C	3I35	4L7M	2RFA
2CAL	3OZ2	1G8I	4MH8	3QUQ	2QPZ	224L	2G10	1BIA	1DAR
4HSE	1YV7	3A54	1WB0	2JGN	1J0O	4G8Z	1JYH	1FRB	4HG2
3A9B	1CX4	232L	3P8J	2DBT	2WA6	1JMW	2E3N	1GWU	2WAO
3HXL	2CM0	3HOL	2D0T	2IPN	1HK5	1YZF	2VZP	3O5E	1QOF
1N6N	1MPB	2ETB	3RHA	2CG7	3MP8	1P4D	1U0L	3HBE	2ZG1
3KL3	1QVA	1JG9	1K58	1CUU	2PYG	2CSG	3BGV	3MN8	1AAZ
4DLH	4EQL	1J1F	2IN8	1L9L	4F2F	2PCX	1DRK	3AME	4FEF
2ZYR	1VYK	2IWR	3GWI	2WFI	2F2X	4H23	4B89	1W45	1B12
3R62	1OG2	2P39	3E7A	3EQA	1D3F	1A21	4HHF	3EKH	1NWU
1U2K	1QGI	2QQV	1CPR	1L31	2VG9	2W6L	1CSK	3LKZ	3G3L
3EA3	2R99	1YJF	3VF4	3BER	1Y4W	1C44	3N7C	1IXV	2C9Y
4BN4	1GYE	1HST	1CFZ	2COQ	3F04	2F5U	1G1V	2QB1	3BDV
1UID	1EH1	3FLA	3PLW	1L51	1EOK	3QMX	3EF0	3NJ5	1RRO
1YWM	3RMS	2Y9U	2DCY	1SCA	1NZ3	3A09	1VR5	1CUP	3P8A
3A07	1C25	3QVV	3K5O	1OAF	252L	4GA2	2H73	3JU0	1AMU
2ZUI	2H17	3OMB	1R8N	1HUF	3VW7	3EAF	3RRW	4JNJ	1H4D
2D59	3SBD	3PIC	2DZV	2YU1	3LJU	1YXL	1JMY	2X7F	1BOX
2QUP	4BH5	1URV	3EML	2JA2	1J6O	3QM0	1BB3	2NWF	1MW7
3MLM	1VJF	1HQC	3I4H	1FR1	1WQO	3EHM	120L	1KGX	2WAB
2YK9	3BOP	1NN6	4GVF	3D7M	4FMV	1L17	3B3V	1C4R	1FC6
1DTP	2OV3	4IB2	4ERC	3BB2	2F1T	1JMS	1GFR	1VOM	3S9U
1HJ9	2I9V	2RCT	2Z3T	3SQE	3KVD	1GMX	2YA0	2ZR5	3RFE
1W1O	2PBE	3IG3	158L	2P2Q	2BSY	3OXH	2I61	3DWT	3LHS
3EKD	2WB0	2P31	1NM8	1H98	4GUC	1BEK	3GMT	2GWM	1CZT
3GQX	3PMF	3NKU	1KG7	3G9T	1MUK	4F69	2YLM	1TT2	2RKQ
1XE4	3RG4	2PK0	4DOV	4F34	3DDJ	1WCH	1MJ4	4AWN	3Q2B
1ZS9	2BJE	1MZ4	4E7E	3MOE	3AZS	2WAC	3QR1	1SF3	2IYV
3MM4	1SHU	1IKP	4EJ4	2IP4	1EK0	1IN4	3A0O	3RCQ	3TQH
1LYC	3K54	3PMQ	1IWK	1MN4	1CYG	4F5S	3DFG	2X9Z	2P0Y
2XKI	2D4X	1L93	1BSB	4AJW	3S26	3LG5	3I1C	2G43	1J96
3W5N	3K2R	2W5Y	2YWJ	2Q40	2WA2	3LLT	1MVE	2OHA	2DPE
2QPU	4G38	3SV9	1FTC	4E7W	4I1H	4EQO	3BZK	2R4H	1WXQ
4FBO	3GKR	1YDB	3USW	2EY6	3TC3	1UF9	2ZJ9	2EYL	3A1W
3EWA	3L42	3S8R	3BSG	3K66	3BYV	1DDG	3VO8	1NUO	2OL7
3IQC	1ZKC	2DX6	4J94	1B7S	3FDR	4ALU	2FKM	3ZH9	1L16
3BB4	3Q6C	3T0C	3VUU	3REN	3VOA	4K44	3L4R	1JXB	1UAX
1I7P	1KR3	1KAY	1HEY	1CNI	3FL2	3NFQ	1YU8	4ARS	1IUH
3EQ1	1D1P	2QVK	1YT8	2X3N	2NAP	1RGZ	1WD3	4EDK	1CXC
1SQH	1P5X	3LOO	3MFQ	2F1W	3C5E	3VPI	2F9S	3TO1	1Y79
3SR4	1Q1W	156L	1RIL	1T9P	3POK	4KIV	4JUI	4DBB	2YY6
3A30	2OXN	1X9Y	3D9Y	1MGR	4IZ8	1T7Q	3DDL	2APL	1TGM
3I0N	1X3S	3D3Y	3D8T	4ESQ	1LLA	2Y3I	3TLM	1FFD	1CV1
1WUP	3GDQ	3AKE	1XKW	2AYH	3TX6	1NLI	1LO6	3E0R	1YP7
4E7G	2ICU	2GKE	2JG4	3LRG	196L	3C5N	4H0I	3MT0	1URS
1E25	1MNH	1QCQ	4IEU	1KXR	4IRT	1Z3H	1W08	3H7R	3HWO
1UG3	1KU0	3FM0	2W45	1GPC	1E64	3GZ9	3O22	3AQX	1RM9
1RB0	1RZ2	1RGV	2CZL	3FEV	3JS8	1X38	2IXO	1YY3	2VP1
4DCC	3ID4	3QRY	3NXH	4IFP	4BG2	4H0S	3ZNX	190L	2V5O
4B6D	3FNK	2QTP	4GPR	3MGZ	4DSH	1EN2	1DGS	2X3U	3B98
1S94	2OJR	1JTE	1H6R	1B80	1M4W	2YOK	3RAG	2PG7	1LW9
3CKF	1QK2	3QKZ	3B87	3I9Y	3G3T	1L09	1TVY	2X47	1OGI

1XL7	3V97	2ZAC	2OPC	1K5U	4KEE	3HVW	3I99	3IQT	3R6D
3G4G	2QOL	1CDG	2WXZ	1U70	1HTW	2BFW	3BQG	1FO8	3P1F
3H57	3Q7C	4EQ5	1E5P	254L	2NOT	1LBB	1VKK	3QHM	1EHD
2QZW	1B7I	4JXJ	3NUF	1NND	2ZID	3NEH	1JI0	4A5G	3TWL
1MN8	1R1C	1S4E	4GCR	3U0V	1OT6	4EAW	1I22	4HCU	1RSS
3EH1	3Q85	1ACF	2X03	2ZBY	3OPU	1X42	1QB0	1BSE	233L
3G5T	2P0T	2FDR	4JG2	3HPC	3PWY	2W86	3RR5	2BZD	2NV0
1QGV	2YPD	1PDA	3LF9	2J1A	1FY3	3KUE	2QIZ	1D7P	4B2I
3P63	3JU1	2XL4	1NTY	3QE2	1E5K	1L70	2OPW	4F9V	3LV4
1IUQ	1IN0	3T92	3H5Z	1U7P	3SY7	1Z3Q	4FML	3MJE	1V5D
3BD2	2IJ3	1GF5	1QXY	1GXY	2VR9	1BY7	4A0Q	1LN4	1YD0
3VF3	2PNW	1J83	2O79	1OFC	3ZIM	3A7I	2QU1	3POW	1OKS
1VER	2C6J	2PDL	3TWY	3NF1	3PVG	1IQ7	3VKW	2EWF	3EJA
2O7R	1ALC	1SNG	3S8P	3PFZ	3RUX	1ILZ	2E24	2EX1	4DCM
1G0L	4K4K	3JVE	2OCK	2Z13	1W9K	3ELJ	31BI	2CND	2DLI
2IAU	2Y7B	1RSY	3T2Y	1L84	3E7E	1HEP	1X2J	2QSR	4ARE
3HLG	1E8X	2WSN	3ERW	1AQJ	4A4P	1OEW	2E0J	4F06	1P4X
4ECN	2HHP	2G6E	1B7J	2CVB	2OS1	3UO3	1BPB	1JHJ	3DHU
2I9W	1OOH	3S6M	2F21	2BF6	1JNX	3OSR	3NJU	2P2E	1HFP
2QCU	1OPC	3DB4	4GOU	1U8Z	3F0Z	2YXB	1RBR	2VGA	3FE1
2I7X	2CIU	2I9A	1UA4	1BPI	2VAN	1WU6	1OT2	2IDV	3DZH
3DWN	3USZ	3QXT	1IUZ	1KYP	1EMY	1QSB	1YLT	248L	2A2N
4EUG	3RGK	2WO1	3E9U	2RAA	2IX0	2H19	2P18	3N94	3RC3
2AWF	4IZO	3V85	2BEN	3GYK	2YLH	4FRU	1PII	2HDO	1GQV
6PTD	3PF0	2C0H	1FAA	1F9I	1PAM	2HQ4	1V3K	3RNJ	1DTJ
2J5Y	2R88	2IYE	1OHU	3PPW	2ERX	2BVF	1F94	1ZDR	3D9I
2J1Z	3E5Z	4ESR	4HPZ	3CXM	1NXC	4GMO	4F6F	3LR1	3PM0
3CQL	3BLK	1QFT	2B1E	4A41	1KXX	1KNM	3U5M	2Q17	4DZ5
3HMR	3ZD1	1LPJ	1O88	3CBD	1OLT	3M4U	3FRV	2HZ7	1O6F
4HUA	2AG9	3CNL	2CIT	2EBS	1AG6	3DL0	2AYV	3NP8	1GW1
3Q3F	2A1K	3LPZ	4FXT	3S6V	1LF6	2RHZ	3M1N	1OTN	3OSQ
1NZJ	2V05	3NCL	4FEM	1CR5	1ESU	1YC1	1ATS	2QGO	2FD4
1GAK	3MXP	1I2G	1H0P	2ZBM	1L24	1L87	3L6G	1LE2	3RRQ
3BFE	2EEK	2WZA	3REZ	3LXP	2Y28	1XWV	3EWH	4EU0	2REI
1S29	3HGP	1GX3	3LIU	3ZUT	1PJA	2CVH	3CVZ	2YHF	2DZW
2C77	2HVM	2NVG	2HL9	1HH8	2V25	1M53	2X4L	3FG8	4I7I
1B7Q	2H74	3UE3	3O59	4ERT	5NLL	2IFD	2A6Z	2J1Q	4B0A
1MZU	4AXZ	1UVX	1YMA	3MDQ	3ZYM	3T1O	3B1L	3COB	1GC7
3RPW	2O3E	3Q7M	4ES5	1NE6	3T0F	4ATJ	3MH9	4AXX	1FR4
2XU0	3L63	1BIK	1BN7	2IS7	1BNG	2HRB	4AF1	3QR3	3FJE
4AKM	2ZIC	3DAI	3DGS	4GCN	2P74	3G0V	3N18	3F49	3FIQ
4J0W	1MLS	3FZ4	1ONR	3PZ5	2W9Y	3FJA	4B0E	2F0W	1L94
1ODF	1LF4	1P8L	1BCX	3HZ3	2A31	3S1F	2JEM	2SPG	4EPX
2FG1	2YDP	3TFA	1NAT	2LHM	1WQQ	1TKE	1L91	3EBM	3ECQ
1L59	3BWY	4IMO	2PGE	2I5L	2VKQ	2BXW	3ZYP	1LSY	1H09
3LW6	2O6W	3FHH	2W4F	2XIO	153L	3R6N	3F9B	2CLS	1US0
3MA0	2J92	1NL1	1I27	2ZEY	1N9B	1APA	1VG8	4J2K	3PFQ
1YUY	1HXC	2XRC	1O8X	1B6I	3S63	2FWQ	1HVF	4EIP	1W79
2O04	2OKW	1N0R	3P3G	2RFJ	1JT2	3EZP	3M31	3TK2	1RYL
4HLN	1KA1	1PYY	3W3V	3M2M	2VQ8	2OCB	3W04	1LZE	3DSI
2GKT	1J3F	2W0D	4I62	1U7L	3FY4	3M1M	3RC1	1FLH	3FIB
1VI7	3IUJ	3TTM	4AB4	3O96	3BU9	1CU6	1L0B	3CON	4A2R
3CMD	3QFU	2P5K	3GHM	1G87	1YMN	3FZE	3U42	1CPN	2XW9
3OLN	2HGY	1WCS	3AKH	7MSI	1GPZ	3RJO	1L39	2WQ8	1CSP
1BLP	1I1J	4JLX	1GFY	2OSV	3EU8	3K6O	4FIW	1YDN	3RFJ
2ZAN	3K7H	3G7U	3TS9	4KNW	1EB6	4EUL	2JJ6	1OJO	4F24
3VZL	2CJQ	1S2B	3LH4	1I3I	2WE6	3K5R	2Z0S	4KY5	1X3O
4GA1	2FDV	3I5V	3W9Y	3PMS	4ANN	3NIA	1F4U	2J63	1SSY
1BA7	3AQ2	1J7X	3OEF	3MFB	3D7R	3PWT	1RC9	1U13	1QL9
2ZKM	3IGS	3FDN	1ESF	4K92	6CCP	2FFB	3MOK	2QDI	3KC6

1NC5	21BI	3DTB	2X46	2WV9	3E8V	1W41	3LI5	2WHL	3OOX
4I86	1TW4	3OU2	1K9V	4TNC	3SEI	2B5M	1MW9	3KJE	2QEB
2F0P	3NUC	1CBX	3VTJ	2HE4	4FVA	3S4O	3OM6	4ABL	3NI2
3SIE	4GL3	1RW1	3AZO	4GOX	2I1Y	1O9G	3PB0	4FFF	2VCH
1WL7	3DFR	4GTW	2NN4	3MN0	1BR9	1IP5	2H0L	3AMC	1W4T
3U01	4G4K	3R6B	3CG4	1Y93	2HB0	1Q4A	3TYW	3U99	4AQG
1Z93	1CBO	3OPR	3UFZ	4K6N	1AIM	2IDU	1H3Q	1M9U	1DBO
3Q2P	3PNV	2NVH	3IHS	1PIE	1BCW	3C3X	1B2J	3FOT	4H7M
4IS8	3F61	1GH2	4FB7	4DUB	3CX3	3C8Q	2ID7	1Q0S	2Y3C
2AAK	3EWL	1QT4	1S8N	1W0P	1IOO	3U9H	2R0I	2FE5	2P5D
1GFT	1VCL	2CZT	1DD9	1DLS	1VFE	2D0S	2JKP	1KVA	2JJR
4H6X	2G7R	1SY1	1XMK	3Q7Z	3BJH	2ZR4	2X1B	4E1T	3SQX
1DLC	2C8J	3L29	2VBU	3F7W	3NEA	4NUL	1RLM	4FO5	4FHG
2YQZ	1OCS	3C4A	1U32	2JC1	2QP9	2CCQ	3JWI	216L	1QQO
1IUE	1DFA	3T3K	2OAY	3O3X	3P6D	2HTS	2J0O	2I2S	1OWE
2QZB	2GH9	1A58	2EA9	2AUJ	1CAM	2UZR	1GNT	3NOK	2OYR
3VN5	2E09	2W1I	1MH0	3EVQ	3DVB	1EX3	1IP6	2PWA	1KJW
191L	2DRS	1HVX	2X3M	3BOV	4B6C	1OUD	3TPB	1YPC	1MGN
2JGU	3DWB	3RSC	3M4X	4G0X	2PB1	3SMV	3RCW	1US3	3GKJ
3LD1	2NOO	4DR8	1EY9	3BWM	3RE1	2HP7	3S79	3H5J	1GS0
3PX8	2VSQ	4GCO	2SLI	1JY1	2ACU	3H9C	2ICA	2QLT	1C8Z
4EN1	3C4S	1GUV	3BPB	3FQ2	4FBC	3EQN	3K6J	1R1Z	1YNV
1WAC	1SBP	2HWK	2DUH	4NLL	2QHT	3ETY	4AX8	2POF	4L2M
3VPY	4LO9	1L8J	1KWI	3GLD	1QMP	2G7B	3P82	2YJ6	1L65
1CUQ	1A8H	1DTG	1BAO	1HRM	3GBE	3CWV	3ZH5	2G5X	1BAV
197L	1TKJ	3LNF	2ID1	2JEX	3VUV	3AXC	1WS6	1KS9	3W4Q
2Y7N	1FSU	3UJC	3IKJ	2I6Q	1OP2	1Q0R	3SK0	2QN4	3RC7
1XOV	2BBZ	3CSQ	1VFY	3BYN	1A6M	4AP3	4HGH	3ZXS	3S0A
3UQ9	3K6R	3G06	4AKO	2NVF	3QRA	1ACC	1DUR	3OW8	2R3A
3TEU	3MEH	1MB1	1QYQ	2PFZ	3CFW	1BIO	3MWX	1P9R	2GPW
2OC6	3N1D	1P2L	3DG9	1KCL	3V0I	2I62	3UXF	3NA7	2QIK
1TQH	1HP1	2AEW	3EG0	2ZXC	1DLY	2B4W	2G7N	2QJX	1PMI
1NZD	4GAX	1NXB	3GU8	2CBR	2WG1	2HKJ	1OG3	2Q4Q	2GF9
4ARV	3UE2	1VBL	3VZM	1C86	4IIL	2FCT	2P2B	4H6V	2IQI
1PEZ	4JDU	3QQM	3HR9	2AJ6	2C9E	1P14	3B9U	1AKW	2POE
1EKL	1YQS	1ZO4	2Q6U	2VWA	4JB3	4F3N	2IY9	2CB9	3G5J
2QDM	1FNN	1OH3	1DYD	1Z5R	3QIJ	3O5V	4GZC	3KVN	1L96
1CJL	1L73	1JJ7	3K9L	2GAE	4F6L	2XAU	3CG3	3AMG	2X9Q
3BBA	2ACF	2GIZ	2ZTG	3ED3	3ERQ	3A3R	2ASC	3US4	4IKD
1O73	1CYC	1S69	3MSZ	4I2S	2E8G	4GA7	2I4G	1ULV	1TH9
2ORX	3RJY	3NKY	1IAV	7RNT	1W18	1VDZ	4EJN	4DF3	2GZB
2R8A	1GS4	4DIA	3HI9	3NSJ	2PY7	1UNS	3TQ8	1BCO	4E5G
2QRT	3U26	2O7I	1YPA	2IYA	1YZT	1L0K	1ESP	1YJ2	2VY6
3F1Z	1HFU	2G7E	3HZT	1IXH	2V6G	4HP9	2F0I	1RF6	1WAM
2GU1	3D0N	1ZBP	2O6I	1WDY	3U0J	3MW2	2EIO	4G29	4HBK
1MQV	4BQY	2W39	2EVT	3BA5	2PY0	3UY8	2MHR	1RNI	3D8G
3A0V	3UIU	1H4X	3BYJ	2FNQ	2DB0	3RJP	4I3C	3N7O	3QPI
2O71	2DCK	1GSK	1CVF	3TTQ	3MVN	4ELC	1UMI	3TFM	1XXL
2IVX	3HQP	3NXW	3EAD	2D4Y	1LPY	3BH0	3TTC	3BK5	3E4G
3VX6	3NYI	2QUG	4DKR	1MVY	1UKS	1ITW	3N4V	1HBQ	3SZV
2NQ5	2O6R	3HUI	3H31	2BVY	1DQ3	2F2V	4GAC	4B8J	3R1P
1SRV	3V9F	3VOT	2CFE	2BJV	2ZP4	2XJA	2CWN	3V0J	3JSZ
1SQT	1HDK	4IGD	2EVK	3PTJ	1Y6I	3AQ5	2A0B	3KHK	3BAQ
1K07	3OIZ	1HNL	3BAX	169L	3T58	2V3K	3H96	1G4I	2CM4
2YXL	1Z3Y	2EVA	2C53	3G9X	3BWD	1B13	1KAX	1FJR	4F0V
2WXX	3RI9	4H1X	3TR1	2FFQ	3IEZ	4HP4	1WU4	4FEU	2MEE
3PPX	2AWJ	1YO7	3PHS	1OM0	2REM	1L10	1NPU	1EXR	2FSO
3KCI	2GNX	1IOA	1DUE	1VK4	4GYJ	2WNK	1CE2	4IKY	2BQ4
2HSG	3U3I	3ZDJ	3QB3	1CVK	1M1H	1L44	1AHC	3K45	2F6H
1CX7	1V9I	3UOR	2XGV	3ZUC	2GAR	2ZPT	4HL0	1RZU	1GC5

1Q8C	4FH3	3DC0	3HGO	2R0B	1N05	2G5F	2G8S	3VRN	3IXL
3DYR	4G8B	1QS1	4E5V	1LYQ	1CC8	2CCM	1RCF	3O0P	1O3X
1JB3	4DLP	2FA9	1JWF	2IJA	1GG4	4BCX	1DYA	3DJ3	2WZF
2GEK	2R9I	1AK9	3P1G	1WKB	3OAJ	8ACN	3RJ2	1YTL	2QGM
3GUX	4L8A	2ZBA	1MF7	4HBU	3GZG	3LCY	4KY3	2FEA	2H6X
4J88	1JLN	4E9B	2W5U	1ZUH	1OQV	1I8A	3A4C	1CQA	4H59
4GAB	1BLF	1OUA	2JIK	2YCX	1P7J	3TQM	1FCK	2GZX	3EFC
1I5Q	2WKQ	3D1J	1JFB	2CXH	3TAH	4GC0	1DLR	4H79	3OJB
1FLQ	2QB7	3IU5	3NHI	3VM8	1BKR	4GMR	3LL0	3O8V	2XVS
2VCA	3HZJ	2P0D	3DP5	3QE5	1PDU	4FD9	3AWE	3KKQ	2POM
3SVJ	2GTQ	1YLP	1DZ4	2BIW	2NT0	3GA2	2PST	1IU4	3V7Q
4DD8	3IED	4ESP	4BCE	3ZKW	2YV7	171L	3UJ4	4FMU	1U9P
3SWR	3SDU	1L29	3A2E	1IQZ	1BT3	1P5S	3FE2	3R5Y	3RYM
1ZR3	3CV3	4I5T	3VSN	1MJC	2WAZ	3TIG	2FJM	2PBN	2WIB
1QUG	1NDU	1RL6	2OIL	1LRV	1HED	2RFW	3VV5	1P5D	3RQ0
4EVF	2C1I	3P26	2PI6	2CDV	4E30	1ZAR	3PV7	3MJT	3V3T
3DAD	3CRG	3VEZ	1SPJ	3KNE	1SM2	4EO1	1YIQ	3NH6	3SY1
4I0N	2DZU	2GDM	3HJT	2PNE	3LX0	3S94	3A3J	3BVZ	4A6Q
211L	1YMW	1L80	3I6Y	2ZKG	1MRQ	2H75	1MDB	3KFO	2OGT
3TGT	3LIM	4DB9	2JL9	2G5D	4JOD	2JHK	1UR1	3GEC	3O7Q
2GMF	2P3K	2VYW	3GYI	3G5B	4EKJ	3LOG	4B9X	3BWZ	2VI6
1R6V	3GOQ	1HPI	1CYO	3PF2	3UOP	1TU9	3VTW	4KV6	2WQB
4FD7	3PKD	1X3L	4FNW	3KZ7	3FJ9	3COP	2V5T	3SUF	4ANO
2ANL	4FO9	3BW7	1V2N	3QR0	1I1Z	1XA6	3EJG	3CHY	3PFY
1YCD	4GVP	3IAN	3HUN	4DUF	1L23	1O6Y	1XIP	3KT2	3SXH
1JDL	3OPE	1WS0	2NSP	2O34	3MPX	4DWN	3SU1	1JGI	1BU2
1HAY	4E31	4F7H	2CUL	2AU1	1YUE	2OFK	1IR9	4I59	1XS5
4HE6	3CW8	1SL7	3OC9	3A0U	3B0N	2ICI	3K2P	2V7K	1T5I
3NZU	4HLT	3DVC	3NPW	4HAS	3S9V	1RH1	3SC6	4EDL	2AX6
4HZS	1AYE	2YMV	2NVE	2WZE	2C6F	1C75	2IFV	2BGS	2QQR
3P1U	3KEW	1CXY	2OX9	1HZO	3KP9	2BIB	3B0Y	1ECE	4I4D
2GML	1WSJ	3UH8	1AOE	1QY3	1S3I	2YMU	3V55	2O9S	4EPQ
4GMV	1XM6	4HJZ	2FST	3U07	3HO6	2GPI	1MZ5	1WKY	3CP5
1A3Z	4AVO	2VS6	4MSI	2NUM	4ES7	2BQL	1OIT	2HBG	2WM3
4H6I	1C1K	1ERJ	3A5I	1ES4	451C	1K2A	4HNI	3UMH	4HET
1SUE	3R5Z	2F0S	1NY1	1EY5	3TI7	3HX4	2BWF	4FBN	3TKT
4JSZ	3HN5	3UJ1	1L74	1UX6	1RL9	3FI2	1SSW	2R7D	2XN4
3TVA	1VLM	4DF7	3B34	3TMG	3CZG	4DPB	2ATB	4DXB	3VNG
1E21	3PZT	3SZU	4JWO	3V5U	2J5W	3KLU	3VLA	1LML	1C7J
2H2T	4EX6	3DML	4HTS	1J0M	1GGL	1YIO	3IU0	3BND	1U0Q
4J3K	2BMV	1H4U	3G39	2PJJ	1M40	1F25	226L	2PAG	3O8M
2II0	1XWW	1GOB	1A44	1AKQ	4KD4	2WZD	1JBE	1JCL	1YO1
3ZUQ	2BDV	1CID	108L	3RBJ	1ABE	3KEY	3W5O	4KIL	4F60
3PFS	2W3Y	4A4J	1B7O	3S0K	1OUO	2YJS	3QY4	2A98	3H08
3DCD	3G7C	2RJO	2WCO	3NHH	1WKC	4FUT	1WJX	1DCB	3S4M
3CLM	1UWV	4AZF	3RPD	1W2D	1HDH	3DRU	1Z3D	1L58	3HZ8
3RZ3	4E94	3MN2	4GQR	3AOK	1B8S	1U4G	2FRX	3L2X	2XYK
2YH5	2ALF	3LFJ	3JXF	2PPP	4JHS	4EYZ	3ZYK	4AI4	3G91
4FBR	1J16	2JD4	2EU9	3CN9	1OYG	1NKO	2YOQ	3ZHN	1Y8C
3I33	2INW	1INP	4DZG	2PQ6	1TCY	1WN7	2VEK	1V3Y	2OU2
3C4F	1WG8	3QQI	2JK7	3DNU	2GGD	1UHN	2G9D	3N91	2XR5
3IC5	3E0Z	3A8Q	1SN7	3L0S	4EF1	2G4K	1AYZ	1WOS	3RLK
3S95	3F45	1GRW	3POZ	3MYZ	1KG6	3FLN	3Q5M	4IJB	1DCM
2X1I	1ZHR	2ODU	1F9V	1OOW	1KAO	1BLU	3GBJ	4AR7	3VQF
3W24	1RE8	1P37	1CGE	3QD2	3LG4	1U17	3TOQ	3NY4	2QBM
3QC4	2WNP	3DON	1NG5	4EPL	3AZC	3U7C	1RY6	2FQX	1Z1Q
3F7N	2V2K	1KN3	1A6L	2OKC	2QFZ	1EP7	1X6O	1OAP	3FT9
3E9T	1H7I	3L5I	2HW4	3NBI	1OUC	1LYE	3FES	2FVY	1FDD
1ROS	2QI8	3O4S	1MJ5	1MZR	1OVB	3ZBQ	1OWQ	4ACO	3PQS
4AME	2OC3	2BNJ	2EG3	1BJK	3T0G	1KBK	1YLJ	1YYH	2YDV

3PPV	3EO5	3L78	3K0M	1YN4	1LL6	1KH0	1TUC	1RK6	1M8U
1PPN	3OBY	3FT0	3L4H	3R6U	3PSX	2IAR	1PVX	4JKU	1DIW
2WW8	1M9Z	2PN5	3Q27	3TIP	3VW5	4K5V	3PWZ	3CZL	3F1W
1IIC	4FHD	2ERY	3ADF	1JFU	2H70	3TP5	3FBY	3DSG	2PMA
3AW5	1OEJ	3H8J	2Z1I	3CM0	1Q6X	3HLR	1JL7	1ZGK	4GB7
3MPP	1F0L	3HPR	3MAX	1LJL	3O8N	2YGF	2XFA	1Y9U	4AFA
2OFJ	2QYW	1BPT	1NB8	2H7O	4APL	1ZMA	4IH4	3PJC	1GNU
1P7I	2GOU	1AQZ	1CO8	3P1H	1ISP	3F9M	1FNL	3CGX	2R24
4H5I	1NP1	1Q2B	1QNX	1L98	2OH8	2GKP	4ENF	4IRM	3QZQ
3OUV	2QWL	3RJU	3TEK	1IQ4	1CHZ	3LPA	2IXM	1V2L	3BKP
3NF5	2FA4	1I06	1KK3	2BT2	1TG3	3M12	3PR9	4KZK	4IQY
2JJN	3T8O	3F0L	3AU5	4GES	1BYB	1JTC	3LO7	4HBD	2JFR
2J13	2IWI	3R9M	3T3J	3KLJ	3PES	4J8A	2OY3	2J4R	1CIU
3EZI	3KNV	1LJY	2XEH	1LOV	2V2Z	3I10	1HJL	2VLU	4ARX
1IJ5	3ZC4	1IY2	3NE0	1FX1	3SWV	2A91	1XRL	2OJQ	4IKV
1ZDY	1MDC	1MO3	3UGK	1YW2	3GG3	1SUB	1FZY	1BZC	2YL8
1XND	4G3C	3SD7	2OV0	1Z0M	3LXQ	2VOS	4B1M	1U9K	3B8Y
2VE8	3BHK	2YLN	2X25	1P4P	2Z83	4HCI	3BVS	3LAF	1H12
2X10	3BYD	1PXZ	3GEQ	3GNF	3HL8	4DOE	2I0K	1JRX	3M8U
1F26	1Y6L	2FRZ	4BJU	2J9O	3AKA	3C7W	1QZ3	1IWM	1N7Q
1WR5	3U7B	2QZP	1H5Z	3AJZ	1BJ7	1ZTH	1IXZ	3EBE	2JII
2FFY	2ZC7	4CCP	3TSS	3QWP	4A0E	3G8D	1YM7	2WNG	2F26
3ZLI	1D5R	1JBW	2VOW	3RVQ	1GNS	1CUO	3ND2	2GA8	3PVK
3BAV	1CKL	2OIV	2P26	2YXF	1PC3	4B99	3V6P	2B4Z	3NHZ
2DDF	4AWT	1HNF	2JCQ	3ATS	3N98	1HCV	4AUT	1CPT	3EY5
2P17	4ANL	3BRY	3EAE	2QFD	3OSY	2BQG	2J0J	3P1A	1EQ6
1OSS	2BRF	3FE4	2O55	3KFA	2H1B	2XC3	12CA	2XD7	1CJ8
3QUT	1DVO	161L	1H4P	2EY5	2FK8	1V9M	2P2F	3EMC	4HAA
3DKM	1DYP	1QVS	1YHT	3AJ7	3KFL	2Z66	1A39	1JX1	1RP1
2Q3G	1LYI	2CMT	4FLE	3TBD	1GY1	1FRX	1IZ2	3OH3	3P0L
3BB3	3C1A	3LEP	1ZH9	3KJT	3EJ5	2ANY	1VHH	1KI0	1PY0
1V10	3AIH	3KKO	1BWZ	3H4G	3FSO	1AKY	3QWL	3KTZ	4F21
4D8N	4DVI	3R0F	1I20	2CKE	3S9X	2UUS	3NJE	2GV0	2NRJ
1TIF	1C3D	2ZE7	2BQB	1OYW	4FD0	2VM5	2WM0	1GCA	1P0H
1L68	3P1W	4KKU	3W52	1CZR	1KW7	3IEG	3BOE	2B29	3HWJ
3H4Q	3O5Q	2W3T	3AQ3	2GW5	1I9Z	2X27	3BIS	3E9O	4ELG
2OSB	1Z67	3TDW	3VGO	1FNM	4GXW	3G4I	1K6A	1ZX6	4HZH
3FVY	2HYV	2E3H	3SNX	2FCF	1UM8	1QJP	1SQM	2GC5	2R0V
3LUA	3SD4	2CYG	2YZV	3SBG	2QVB	3TG8	3JUG	2GP0	4A5T
237L	1FGQ	1FAN	1GP6	3M23	4A6Z	3MUI	2YB6	3EJI	3QM9
3S6B	1IXI	2Q4W	2Z2I	3LAH	1VS0	1S0U	1NAZ	2XY1	1L15
3L0B	3EP0	2ZFY	2WA5	1WDR	1BA1	1SNO	2JHS	2I4S	1LL7
3JSR	3PAF	4AZZ	3ZSU	2OXP	1ETJ	3A1B	1LFI	2R51	1YJB
2J4B	3AV3	3U9W	1E63	1GA8	2SNM	3KAD	3P1X	1OZ2	1JCI
1KCT	1POO	1P09	1QUF	1DVM	3T7Z	4A2V	2P65	3CVM	3SIJ
3H2H	3TO5	3V2W	1J97	2Y9R	3D2N	3HD0	1LKX	4HQA	3NQV
1ZLM	3A1A	1NA0	2NNB	4E9O	3UDD	1LG2	1TXJ	1ZU0	3D4G
1OZL	1WNX	3HSU	3U5L	3EG3	1J87	1IAL	3B5Q	4GXP	1QNR
1U71	4KE4	1FNI	4H27	2BCE	1OSH	1RRV	2WTW	2C71	1LII
3Q48	2QZ2	3HJZ	3PE9	1SDW	1FMK	3KD8	4F3S	2O0J	238L
1ANN	4ADY	1KMQ	3EIE	2DDX	4DVZ	4J8P	1CKC	3ZS5	3W3T
1SZ9	1PE9	2OS0	4DI9	3D5Z	2ODJ	1WDN	1SY3	1MFW	1VCC
2HY7	1WY6	3V33	1T77	1YAN	3KHW	3BCZ	4GSJ	3DCN	1KY1
3IDV	1KMO	3OG5	3QOK	3QD9	2FYE	2VFK	1T1G	3E5M	3OIO
2BQO	1C92	1CPG	1M8N	1PSN	3GKK	2BVU	1GOL	1MIL	3VC1
4G3T	1V4Y	2ALR	1Z81	2A39	2IGD	1BNF	2Q9V	3F0P	3BHW
4I1Z	2WBO	1A8P	3APS	3D21	3AY2	5CA2	3BON	4FWI	2NTH
2IA7	3S4L	2GBM	3QYA	1W3H	3A50	2VUP	2W23	3QZU	4BKW
3OWR	4JGB	1BRK	2B9E	2P1G	4IL3	2Y6O	3MHD	1FE5	3MC1
2FZ4	3Q6X	3JZT	1UTM	3BS7	1R5L	3S5M	1BOQ	4EIS	140L

3AAG	3SA0	2HI0	2GQ0	1QUB	3PAG	1M8Z	4FCZ	3BDC	3ZH4
3UW2	2TSS	1XKT	1O1Z	3IN0	3G6S	3HZX	1Q4C	3GA3	4F4M
4H09	2QEQ	3FOG	3OPX	1N7P	2F46	3AUM	1ERM	4ILJ	1TSF
3C81	1L32	2Q4F	1AQL	1VAV	2FBY	6CP4	3EVF	3HBZ	2VMH
1F20	3NXE	3RPT	3OUI	2X6R	3P8W	4DOO	2IM9	2E56	4HS5
3R8D	1QSW	3LFF	3MFC	2QET	1JTI	1YPY	3PPH	4DND	1YHG
1QDD	2YEQ	1HFC	2I47	1KDA	1CHD	2V9K	4JID	3D3Q	3HBN
4E6Z	3ZVV	2PNU	3NNB	4HBR	1GXO	4E2B	4KQZ	3F06	2IN3
4DV8	1B9A	3S6W	3ZCW	3NOL	1BZ4	3PSF	1KVC	4AIE	1QYO
3DEO	3PEB	2WE2	3NIS	1J8Q	1AQB	3A1U	2J1V	1KR0	1RJB
1RDA	1L37	1W8K	2PJD	1DYE	1YHV	3HGL	1GLN	4EFG	1N44
3S2J	1K40	4K61	2V1Z	4I6I	1AOA	3OYO	4FDC	3ZPY	3OQ4
2ERV	3B2M	3SBS	1W8A	1AMO	3AWP	3OF1	7ACN	2GCL	2P6W
1ZWP	3LJW	2PR7	1L0Q	3OLX	1QIB	1X6L	2H5U	4G7W	2QT1
1ZC0	1XAK	1P15	3DXY	2IMQ	1W8J	1GB6	4JCC	2B3Y	4E9E
1FQI	4AIW	3B7N	2GT1	1NQ6	1GPL	4FXV	2IAT	1GEN	2V9R
3O3U	4G57	3D6J	3U45	2AWL	4BGQ	3VKI	3E73	3CMP	3PPY
1ID2	3ZD9	1L82	4BI4	3FAJ	3HNG	3VKN	1XQO	1AF3	3STO
2ZFI	1R2I	2ESA	2HBJ	2QRI	2A3U	2PZ1	2ZV7	2O9O	2XXZ
1IR6	3QOY	1AH7	3AB5	2JGP	1DSL	3DH0	1RHS	3O6U	1VPR
3PL5	3C8Z	3ZBO	3NVS	3COX	3LHA	1SVS	1G9X	4DRZ	2JBV
1B1J	4ATW	2JKW	1IG5	1SOG	2PI3	3IBY	3PDV	3LHO	1U6M
3A45	2D3I	1N1T	1JHN	4AW5	3SH4	2R65	3BVG	3FFZ	1LCT
4DRV	3ZUS	1CTJ	1D9X	3PLY	2SNS	2BJW	1DXJ	1YU7	3AXI
3RMY	2W2I	1B5U	3EKJ	3UC9	1TQG	3RLD	2X9X	1R9L	2V75
1DBP	1L95	3HOJ	4GS5	3M7V	3N3C	1NPH	2IY7	4FEK	3VVJ
1G66	1ZE1	1UN3	3IDP	2V1L	2APT	4I8I	3RJ0	4GNM	2EI9
2WQ9	1JM1	4HLX	4EEJ	3U9O	4HGT	2IEW	2QC5	2NN5	4H1Q
3HRA	2XJU	1T5L	1OFF	3OD6	3B6X	3AML	3KWL	1HXN	2X30
242L	3VOO	1YN5	1IZJ	1D0B	2FH7	2IC9	3UMF	1IS1	2F1X
2Q4C	1IST	3MTY	2X55	2ZRM	1EZ6	3UR6	3IA4	2EWE	3VDH
1I9E	4IG1	3RMQ	4F9D	2AF5	2Q83	4AYJ	1VZ2	3DAO	3IGN
1CMN	1LJU	3H0P	3BGI	3L4E	1G61	3U50	3H0O	1OI8	2YT4
1B20	4B4R	1J9M	2W1S	1QUL	3N71	4DE9	1V2R	2NQY	4AFK
3ZZO	1IEJ	4GX8	162L	1VZT	1OHT	2CKX	1MSJ	1UN2	3UHP
1KMT	2H3W	1TL2	1E43	1L0J	2GRC	3GSE	2JLL	4H0W	152L
3RGQ	3M7R	1J2L	2J0I	2X12	3PDT	1VYV	3QDP	2Z8G	3H4C
1GGV	2Z1G	1BQB	2B43	2GFT	1T3Y	3HSG	1HMT	2NS0	3H63
3PM2	3N0K	1YAT	3FBW	1RBU	3I78	3IHV	3O3R	2H5O	1BOO
3UZX	1JYJ	1VR3	2Q3U	2F4J	3U0K	2ZP5	3HNR	1Z6Y	2OGS
4ECF	3FIX	2CWS	3T5A	3PG7	3MYK	1HVE	2JE0	1G8P	1BS2
2XSG	2XTV	2H7J	3QL1	3LBL	3U16	2Z1D	1EM2	4CLN	1B2M
2B2A	4GIE	3ZDK	2OCE	3EZO	1AVC	1SL5	1GX2	1LXK	2WQX
1QLJ	3BN0	1S50	2Z79	1AKR	3G3X	2JJQ	2FSL	3RIJ	4ANX
2F51	3LJN	4DGP	4AR9	3K7J	2E3Z	1UMZ	1OP0	1WJ9	1ZC2
3NFH	3KNQ	1ZLD	1RYQ	1X9U	3NIY	2A90	3ZS3	1KRH	2QZI
1TS2	1F21	3RQ4	111L	2JEP	1N6K	3PXC	1LS1	1T7N	3RSM
4KF2	1KXC	2O3T	4D8O	4FWE	3D3A	4DSU	1SRP	1PZX	2BRA
2WUT	1YQT	1T6D	3IVF	2R58	3VTU	1YD3	2YD1	3N15	164L
3LVR	2DZP	1RA6	3Q0I	3H4Z	3LI3	1TP0	1YK3	2AX0	2VZN
4IIK	3VOG	3UAD	2O6S	2G3H	2F37	1CKE	2QV5	3C6A	1N5O
2WLQ	4FCW	3EM5	2F0L	3GMU	1PGS	2BOG	1L88	3VPE	4GKP
1L30	3RQZ	1TCM	2BER	1Q04	2QHE	2CNQ	3BCH	4E1B	1XRR
3GEN	3UY6	1RYB	3UR8	2CDN	1GBX	2OIB	1F9T	2IXG	1R6X
3D80	3K7X	3RBS	4KKV	1Z4R	2XU3	3FM6	3UCP	3LQ0	2BAQ
1GVD	1C90	4ECO	2BOO	2BSB	3AUK	4FS0	1L48	3LDA	3EOX
1ZA4	3O85	3C4C	3OGB	1LVK	3TOW	1GEB	1IO1	1FXI	3G9Q
3S8M	1O7O	1LP8	1ZUU	1BA0	1LYY	3B6H	3HJR	4B3F	2IAS
1YXA	1DZF	3F10	3SV7	2V8O	2D3N	3ECM	1G6B	2VVN	2I1U
2ZD8	2GY5	2MSI	3LVU	3PZ6	3GAX	1OIO	4FNV	1KG4	2YHS

1PGX	3IUG	4IQZ	3A27	2ZP3	3W37	4FCN	2PUS	3I8A	6MSI
2G9N	2BHU	3EDH	2HZH	1Z97	2OLG	2VLC	1RIE	1H4C	2W0A
4F6P	2XAN	1NFO	2CY2	2JE8	2C3G	3DXQ	3UQ8	1Q5M	2BK9
2YV5	3PL1	1MKR	3G01	2CIS	2Z8L	3DU1	1DWU	2PWQ	1KSU
3QAP	3RHX	2IC1	3EDY	1MCX	3OY1	2OXC	1L46	2FMJ	3C7Q
2RJP	2OLU	3LF3	2VLW	3OMX	3Q71	3SST	1T6C	3AHR	3UL7
1LBU	1LQT	3D1R	3ST0	3SOJ	2ZZE	2R0J	3AA2	2B4Y	2IGC
1ET6	2CFM	1JIH	3F9R	3O5Y	4DA1	3JR1	1CFY	1G3Q	3LLB
2JHZ	2V84	2NNJ	1NAA	4ALY	2QGG	4AWU	1GOD	3L2A	4EGV
1W24	3T47	1DBX	1VZW	1TQ6	3O3Q	198L	1UGD	2QTZ	3PSP
1G7F	3F9D	1L25	4KTA	3F0I	4EWN	3K8G	3HYN	4A29	3OYV
2PH7	5EAU	1J8R	1F5C	1G1W	1CV0	1E30	4EFE	3LY5	1W3W
3KN7	2C6U	1I1N	1HD3	1OYU	1IN6	3VNB	2IF4	2O69	2FI0
3P8P	4EB0	3SDZ	1SV4	3N17	3P2H	3DJ9	1I3S	2DSX	3P2J
3R8Q	3UGU	4DVC	2B1K	1LYK	1KIV	1MH8	1S1E	1TF4	1A7E
1I3P	3PZW	1RG8	3LK7	4FLS	3D5J	3IHO	2NZF	3QYD	1G0G
3T10	4A9V	1EKG	2CC1	2A7B	3ADJ	3E9L	2XNQ	4E7N	4AMW
3L7Y	4B9I	3VZI	3D6I	2W1J	2VYI	3QH4	4A42	1ALQ	3TC6
1ISN	2ZO4	3Q7X	1JCJ	2V6I	3Q2U	1ZTQ	1JWT	4FSX	2YV2
3DKV	3LMM	1GBS	3QUB	4GBF	1P2X	3DKP	1G0K	4KAC	3F9L
4KPG	3B5J	1RO2	1LGY	1T00	1TJY	2VQX	3TK3	3JTY	2B9F
2ION	3CDT	2RBI	2X5N	3I8S	3KAP	1TEG	1QQ4	1M5I	1KQW
2F60	3V45	4FIE	3FZ9	1U2X	3D22	3SG7	3GMF	2XDE	4GEI
3U0D	1WNS	1KWF	4ELN	1WCW	1PKR	4F11	1O8V	7CA2	1NUK
2XQX	2FNC	1MSK	1XQG	16PK	1NJF	1L04	1O71	1L13	1GS9
4HOR	4GKF	1E4Y	1EH3	2QFT	2IVD	1MYM	3H8Z	1XQW	2ZHN
3MYE	2X1P	3DRM	1OGW	4HK2	3CEG	1PVL	1SBX	3Q1C	1J6U
4ENZ	1DI4	3G0M	3PF7	2QZE	2IBJ	1D9Y	1IA5	3L6N	1FRH
3H1D	2OB0	3LOQ	4IQA	1LAV	3KW7	1CH5	1D2W	1C3P	1ROC
3ERV	1PYL	1CWV	3RY5	3MWI	4FCH	1ANX	1GYV	4E1Z	1H4Y
1EG3	3HVN	3RL5	2BZL	1P9A	2WBF	3OS4	1HVD	3M88	2B50
3PSI	1NAG	1V0B	3G3O	1YX2	3LL7	3UAN	2EYP	3N9B	4FOJ
3QPA	3QY7	3AKS	2RE2	2JDF	1TGO	3GJW	2VCS	2RCA	3NPV
3O33	2EHG	3PE7	4ET7	1B5Z	4H6T	1G2R	2YE7	3VA7	2P3J
2F0D	2XPZ	1Z16	4FVQ	2QC9	1BYW	2VQW	3M6W	3O0Y	2V7Z
3EXY	4AGU	1L56	4DTF	2OSH	1FOT	1MIJ	3CTQ	1WCU	2CUA
2OR8	4ESS	3DMU	2REP	3PIW	1HAR	4AP6	1US2	4H3X	1T7V
3HR6	1FGY	4HOU	1FLP	3FA0	4ENG	3PDS	1ZIU	1GD8	1VEO
1H6T	2NV5	4H3T	1Z2M	3NKG	2BDZ	3TEG	126L	4IPF	2B7U
1L61	3DMI	1ODO	2HED	3B0M	1O9Z	8I1B	1D9W	1HJ8	1D2P
3Q6O	1V6S	4B50	3BYK	3QC5	2WML	4DWO	2BH4	3AWG	4EI2
2HXX	1T2X	3BQW	1YMK	1KG5	3MHW	4HWN	2YA8	3UQI	1FVJ
2RFB	3RLN	2ZOC	1UJP	2I75	3TVR	3KA0	1F2X	3FYC	3LGK
247L	3NXQ	2VWB	4I37	1JBZ	1WVH	3FQ1	4AHI	3TW0	3ALG
2I6G	2QAJ	1GLO	4HVL	1WLF	1L28	3LXD	2P14	4AWK	3E8T
3QMM	1XKG	2RAV	4EQM	3RL3	1ELT	2IV2	2WRY	4ACJ	4GC8
3JZJ	3SIQ	3AHQ	2X80	3O6N	2WAU	1ZCM	2MED	2IW1	4IOY
1CP0	4H6H	3EH2	2AJG	1K7C	3I36	3RLG	3AJ4	1VPE	2BQI
3FV5	1PBT	3FOA	3TT2	1R6N	1JT7	213L	3SK4	2QPQ	1W8T
2YEX	3QYT	3ZR0	1IYS	3DKB	1OQG	2F1J	1Y54	2J0R	2ZZA
2WI9	3ZZP	3BFK	4FIB	4J7O	3UX7	2CIG	2ONQ	2P0B	3CV6
2OPJ	3F57	3UNX	2YA1	4I6G	4KF0	3G9H	3SUK	4GF6	3BJN
1OJJ	1LIO	2ZYH	2CJL	2QEP	3IV4	3TCH	2RIK	1G12	1IDJ
1L9Y	2GNO	1K8Q	1KXY	2DEJ	1WZV	3RMX	1I2H	3OQC	2DVV
2XEN	3AS8	1FUK	3MYD	1MGP	1R9H	1HCU	3LTI	3EWS	2BEB
1M15	3BM8	3FKG	3ND9	3D4M	1EUP	2DYI	1ZBM	2OJ1	2GNC
1L2L	1GII	4LOE	1Z3U	1CVL	3DGN	3AJC	1IRW	3M07	3RNL
1SX7	1J77	1L27	3NE9	2Q7S	3FGX	1UG6	2FSJ	2Q00	1W8G
1YRV	1KGS	1I77	3AQP	3LLK	2Z62	1O0X	3I5B	4E57	1EY7
3NZT	3Q8G	3O4P	1ST3	2HRY	4A1H	3D2B	2Q4B	3A56	2ESK

1QH0	1QGY	3HIS	2D7J	1S1F	2VU9	4F91	1FAO	3KG4	1M2H
3I5P	1G55	1EFC	1M0Z	1GBW	1LHT	3SHF	1L6J	3LUC	3TX2
2EY1	4KND	2HCZ	4IEH	3M5L	2I5U	2P87	1O4R	2VWK	3KJD
3BO5	3NZZ	1HXG	3IWI	2FFM	1Q1H	4HDE	3F59	1CKF	3I2V
1A7V	1YPB	1EY4	3Q93	3LFT	3GL9	1YZ6	4HB5	3SNG	2BME
1YGE	1GE2	3BKK	4ILV	1OXZ	4ELJ	3QGI	4EFF	1V9F	1NM2
1T1F	3AY5	3P90	2WNO	3U65	1Y0M	3PJQ	3CBW	3AX1	3EEH
5CYT	1E6F	3FE5	3VYC	2O89	2X07	4IJX	1E5N	3S0Q	3AOX
1GE1	3PIE	2AKJ	2GUY	2BW0	1IN7	1PB7	1NWA	1QD5	2ONS
1TBY	4FDM	2OQZ	1Y81	3K77	2X0C	1RGK	1PC9	1CJ9	1UWY
218L	1TUO	3JTB	1NR2	2ZBX	2BQC	1R9O	1GGZ	3W25	1R6F
3QOL	3UGC	2IMR	2W3L	2VYQ	3UN7	1HKG	3TBK	2BM3	2XBF
1PZT	3MKL	1RI6	3G4N	1CHK	3FWK	3UPA	1E61	4K73	3DF7
2XML	3ZFN	2OZF	1HH5	4DGD	2FVG	2W3J	3FQY	1UN5	3RAB
1OBA	2VSY	4BF3	1ZR6	3TEV	4ARU	3PME	1ZUA	3HRG	4A8U
2XUB	3DOA	2EID	2BR6	1GMI	1SPV	3QCA	1PHP	2GZS	1Z8P
1H4H	1BC0	1CK3	1FKW	2XO2	4F1J	3DOU	2QDC	2YBU	3DAE
1U4Q	1XT8	3U5R	2RDG	2F57	1KSK	1R3F	2FVL	1BRY	1QFG
1OCN	2ET6	1GWZ	3BB7	2FNN	1RYO	3I86	2EQL	2J8K	1B7L
2ZXT	1R2H	3EO7	3M3P	1ONL	1KHO	2F4W	4DGM	2IA8	1ENK
1QHT	2JDC	1TMM	3MVI	3PBH	1IYO	2IMG	3CR6	3EZT	3CH4
3CQF	4EOE	2ID9	3CMN	2PNQ	1PBG	1KTE	3OPH	1XP4	2WMC
1KRN	2DYT	1L53	1KVX	3SRY	3FK2	2OHW	1F2M	1DXS	3MX8
1MOZ	2BCO	3TU6	1TZQ	4J8D	2BMW	3E1S	4GKC	2XYD	2HZQ
3IMM	1YZH	1DPO	2GWN	1G02	3MDF	4GQS	1K1B	3B7Y	3AQ7
3LY6	4AX4	3FAW	2WKR	3S6E	3IHG	1BFG	1YZQ	1GZE	2CBP
1UCD	2X48	2GPR	3RR6	3AJD	1JVN	2QQW	3CFY	3RCB	3P4H
4K3F	1GAI	3N6M	4B4O	1EPW	3DHM	4E15	4E1Q	1BEB	2DT8
2HRX	4G2A	1KID	3GHC	3BHD	2QPN	1A6Q	1Y02	1KO3	3RRX
1VZO	1DLW	2OWE	2ZW1	1O9H	2B49	3DZX	3D2Q	3M4Y	2OAJ
2EWU	2P6N	1TC8	244L	3ESC	1DL3	1QPG	1HKM	3SW0	1SBI
1JL3	2OG4	2XEX	1R85	2O14	3U47	5CGT	3D6E	2I1M	3AQV
4J5T	3C71	2XBI	4EO6	1OZ9	1L0F	3IM7	3L4Y	7FD1	1GWM
3ILC	3TYI	1SGT	3OZX	1H1N	4GNI	1W0H	4EYE	3CPI	1P5J
2A1V	3NRN	1NDB	3LNB	2F6B	1N1X	3VFD	2VZT	3IXM	1N83
2FBO	4G31	1B9K	3NWM	2AL7	1GCI	2QX5	3Q69	1V76	3W4T
2GQP	1XJZ	1SBT	3UWB	1CUB	2CWD	1W74	3NLL	1CCJ	1G8A
1Z2N	2GJJ	2GHS	2BBT	2J5X	2HUJ	2CBZ	4EXU	3H7U	1NA7
176L	2PTK	2NQW	2ODP	3F85	2A28	2VWX	4GRF	2O5W	2W7T
4HW6	1TAQ	1V9H	4CGT	3Q6Z	3L4N	1QTD	3L86	1FGM	3PUC
2F2W	2RBM	4EPR	1QJW	4KVF	1TXX	3C8Y	3SBY	1F4P	3RH3
4ATE	3UF8	1F5B	4EYT	3DS8	3KPA	1KAB	1I3U	3EAP	4HB9
1PSW	1B04	2CN3	3GD0	3HZL	3S4E	2Q8W	2VX7	3KX3	2O5F
2F0T	1LY8	1RUT	1KXO	1XNC	3FK8	1KTQ	3FJO	4EEP	1MWC
2ZNR	1HPT	1PMH	2IFY	1MEG	4JPQ	3EFL	3QRL	3IVE	1U29
2ZQU	1JIX	3UON	3MMD	1DJA	3HX3	2C7X	2EPG	3ASM	2YXP
2AA2	3SIM	2J1Y	2Q7T	3P5S	1STY	1IRV	3L87	2OML	1ILE
4KK3	1R9W	1FCA	1MDP	3EUR	3GC8	1JYK	1GQ5	1I4N	1EX9
4IKM	1BF6	1PC4	4HJO	3LIJ	3V2I	1ZD1	2Z6O	1OS8	1M6I
3E1T	1G6I	3LS8	3OLV	3OLI	3Q6B	2HKL	2DZT	1XHC	2QFL
3BIR	1OQH	4ATF	1L33	3PE5	3SU6	3HY5	3FG6	1EUE	1ND1
2YJR	2VNG	3U81	1X9Q	2Y23	1G6S	1P50	1NH1	1RXD	4FSH
1LJ5	2QC3	3RJX	3HRP	1DPP	3IAR	2BK0	3TKM	2PX2	2IAX
4J37	2CTH	1R8M	3QJ4	2WZH					

Table 10. List of PDB IDs of all new homodimers (Test-set 2)

4D28	4QDS	4OIT	4IZW	4UTT	4NEI	4NEX	4PUA	4QGI	2FHE
4TVI	4BOQ	4QOA	4C6E	4I9E	3W5X	4GTU	4J72	4I02	4C6U
3WFI	4O5F	2GR6	4OGD	4V0Q	4NUR	4RA5	4XOX	4Q1V	4NOG
4LUK	4MO2	4RZ0	4OMZ	4PTS	4L1A	4BJP	4NFH	4W72	4ISY
3WSC	4Z04	4NYF	4HGD	4YWQ	4RUQ	4OUN	4BT7	4O4O	4QEZ
4V2K	4QL4	4OBU	4S2R	4MYP	4KA6	4OAA	4Q0C	4JEM	4D1Y
3ZF8	4C9X	4C51	4LQB	4PBS	4RIT	4KQF	4WXG	4RUF	4P5E
4NPB	4PUC	4Q1Y	4RXO	2YNA	4LA1	4KV3	4KES	3WAF	1BAI
4MA5	4K5S	4RDZ	4LNS	4OM8	4K8O	4OML	4DMC	4J7R	4NEL
4Y0H	4PIB	4NZ1	4L51	4WP0	4O52	4O2D	3VY5	4UTT	4BAT
4QDK	4YL5	4CVS	1NJU	4XA8	4TM5	4LMI	4JTU	4L3V	4O4V
4MYZ	4UX7	4RMM	1F08	4MKI	4BI9	1KEQ	4C0W	4K2A	4IY4
4V12	4CW5	4U0W	4WOP	4KGH	4CN6	4S0M	4RA8	4CN1	4KU3
3WYO	4CF3	4M2M	4GS1	4N2M	4AZR	4IY3	4JHB	4CXT	4LTA
4LHD	4M6Y	4PH9	4CX2	4Q1X	3W91	4NZV	4BT8	4MF5	4S1H
4PDX	4L2C	4CZ8	4QF2	4W66	4LJ3	4O18	2YNW	4BYY	4V23
4LAM	4MUZ	4OQP	4MF1	4N12	4CGX	4CMZ	4MRI	4AZO	4L4J
4QGR	4PL0	3WW8	4K59	4LWO	4Q04	4CJ7	4LUM	4US5	4M71
4FDA	4O5A	4MPF	4JEN	4L0K	4L28	4KEU	4RK0	4C12	4MAK
4I6T	4NAO	4W8J	4TR8	4CBS	4H4I	3ZGY	3WUR	4OUO	4W4N
4PVV	4TKZ	4WUJ	3WJ8	4TL3	4DK2	4BMX	4L0D	4LI1	4NLJ
4QGK	4CMD	4Q69	4WNY	4MAM	4RL2	4L3K	4BG9	4K5E	4MF3
4YER	4RL6	4MZM	4PQA	4C1B	3WBH	4LE5	4Q1E	4UPA	4R5M
4WK5	4L7B	4UAK	4IV8	4BGA	4Y30	4KQD	4LOJ	4NUQ	4NMH
4UNU	4MAF	4P1V	4O19	4GWO	4NOZ	4MUR	4RYK	4ZAS	4P0E
4MYJ	3ZIG	3WNZ	4D3U	4UPG	4L06	4WPC	4L6Y	4C5W	3WMD
3ZI0	3ZIH	4R2V	4I5Q	4N2C	4KG1	4CNN	4LJI	4B96	4Y0M
4O98	4RT5	4RCT	4O50	4NDK	4C2H	4BEI	4CBR	4C5J	3ZCD
4P96	4HHV	4NB1	4LUY	4XI2	4PZ1	4RRX	4YYC	4LDI	4Q6R
1ZPK	4MEB	4JDA	3WAD	3WXY	4CVR	4O7J	3WYO	4Q1W	3ZBG
4KMM	4N06	4Q9V	4P22	4INE	4PW2	4COB	4Y67	4EIL	4LFU
4Q5F	3WF3	4K3G	4JAU	4QVS	4C8B	4LDV	4WPE	4ILU	4I6U
3X0V	4H0R	4YLT	4NVR	4D0A	4X7V	4NN1	4UTU	4KFG	4GNJ
4P61	4CWZ	4GKM	4WJY	4TNU	4XVV	4CET	4S39	4D5J	3WCO
4L95	4WPD	4NPO	4OVM	4LGQ	3WBR	4CHS	4LAL	4BJN	4D6K
4CP8	4QR2	1K2C	4KDE	4HTX	4XF5	4PY4	4N1D	4LS6	4L9W
4QXW	4USS	3WD8	4CJX	4C11	4L1C	4MUM	4O2H	4OPE	4PZK
4KM3	4Q9C	4NTE	4KCE	4PH6	3VY2	4LDU	4IYS	4FRA	4S28
4CKN	4NV4	4KC1	4KXV	4KJD	4D7M	4L76	4HZI	4LE9	4RHA
4QGT	4L57	4OQY	4YSW	4BKQ	4LDP	4XD1	4XK1	4OVY	4WBQ
4KRW	4W8I	3W9B	4NQF	4W6S	4C2S	4UQV	4HKD	4NQ1	4GQZ
3W65	4PN0	4O6B	4BWZ	4RBR	4X9K	3ZPX	4LC3	4OMV	4Q0I
4BOL	4PBR	4RMB	4CJN	4KC5	4CID	4W6C	1Z8C	4IRH	4BNE
4U6S	4MDZ	4MJ7	4O15	4BWV	4TR7	4O57	4JAK	4NR2	4BRC
4KZ1	4CDH	4JPF	4C1O	4YN9	4OCA	4MND	4OMM	3ZBK	4P08
4MDC	4QXP	4Q9A	4OP7	4LOI	4TR6	4D3V	4OJR	4RM1	3WJE
4Q5N	4K1V	4NJV	4QHJ	4S2A	4Q52	1K1U	4YWN	4D3D	1VP6
4JEJ	4OJA	4NFL	4QR8	4RDA	4LDK	4XLY	4U1F	4Y2H	4KEV
4BMM	4OK4	4NPT	4RIF	4MDU	4U77	4MG4	4P52	4BW5	4RKC
4IS7	4MPM	4Q5Q	4O1Z	4IK8	4V2X	4TPL	4N83	4XWS	4ODJ
3W79	4Q2H	4O1G	4LOB	4OOF	4QDY	4MF7	4DLN	4RPO	4NG6
4RK6	2I99	4CUL	3WGH	4U64	4OJ4	4KVI	4DM7	4U7X	4OCI
4P7C	4Q60	4X1Z	4N5V	4LDY	4C8A	4BXI	4P4T	3WPW	4N2E
4UDS	4JN9	4LHS	4PUL	4WJL	4KL6	4KQK	4POC	4MAE	4RMF

3WPS	4RXJ	4K5C	4RZG	4QJN	3W62	1Q3O	4IP5	4NEZ	4MB0
4XDA	4X0R	4MNF	4O5V	4OFX	3HNO	4I97	4IT6	4OV4	3W6P
4BS9	4KPR	4MHX	4NV1	4KLA	3WLC	2WLV	4QKU	4W91	4O87
4CVP	4J2P	4IBG	4OW8	4MH4	4PY6	4HR0	4Q34	4O5Q	4R9X
4OY3	4R33	4OK5	4PN6	4C7L	3TLD	4BF5	4MYC	4MRS	4KYX
4MVC	4LP7	4W97	4YN7	4L9M	4O6Z	4UVQ	4D02	4Q25	4HO1
4O1D	4UC1	4WD2	4PET	4WUM	4UMP	4M9D	4MZE	4XEU	4H5U
4BAZ	4M03	4Q2Q	4W7P	4CU5	4O1B	4MKN	3VWR	4LU1	4QAR
4WD9	4BHW	4CV4	4JZP	4KM4	4N9U	4L2B	3VWL	4XDY	4LJL
4YR1	4RG1	4N18	4S1A	4N2K	4MMN	4BMZ	4O0G	4OIV	4UZV
4M6A	4LW8	4OQ4	4X84	4V0P	3WGQ	4KMH	3W36	4NY2	4K07
4M0I	3ZHR	4QRI	4JEL	4GIT	4QGE	4C98	3WXL	4WJA	4L0M
4IG2	4QYR	4WX0	4CUC	4KP7	4BEU	4LIR	3WMW	4G1P	4U04
4Q6K	4MUD	4UQU	4NSQ	4HEG	4HA8	4LMB	4JS9	4KN8	4V0R
3WRA	4Q9M	4PTE	4W82	4K2M	4JVI	4OMN	4UR6	4NAX	4QV2
3WF1	4JB6	4CNG	4LH4	4QAV	4LCJ	4RP3	4LMW	4RT7	3ZOM
4K4Q	4IAN	4BSW	4MCJ	4ULV	4BX2	4YFJ	4RYI	4KCC	4YTY
4OOX	4P9G	4HWS	4N2F	4K6K	3DQZ	4KY9	1TO3	4WKY	4PFH
4K7N	4ICS	4X7U	4Q6U	4L75	4L9T	4L56	3VZ2	4KLR	4RI6
3WA3	4NDS	4QVB	4MO7	4KQI	4CX5	4KR5	1DAZ	4DMH	4CVQ
4MCR	4CE0	3DCR	4NUM	4ICN	4NUB	4O96	4UXL	4PXB	4ON3
4ZAH	4OKY	4IZ6	4WHJ	4RKQ	4O60	3WQF	4OBQ	4J0E	4LRQ
4OMP	4WJI	4WZU	4PU7	4OMH	4MN7	4NJH	4IQL	4CXJ	4POB
4C11	4TV7	4KET	4LQD	4MF0	4UZI	4LMA	4IFT	4NJY	4CS3
4OPH	4O17	4OAH	4N20	4LZV	4PZU	4KGO	4OT9	4IG2	4LFI
4MQB	4HNN	4K2E	3W63	4BKD	4M5P	4K09	4G64	4C6N	4LL7
4LU6	4CWE	4HQ0	4UNK	4O0D	4POD	4Q7E	4XIW	4TRH	4MEI
4NA1	4R5K	4MQ0	4BKM	4RK4	4P5N	4M9U	4TSX	4QS6	4IG4
4UXU	4OWY	4O7U	4LDQ	4PPY	4YET	4G9U	4NNZ	4Q75	4M8Z
4BQE	4JIS	4KZP	4IX8	4HZF	4C50	4V03	4LXX	4PBC	4UC3
4M37	4Z0P	4S3M	4U0S	4O1E	4TPH	4JM0	4Q86	4BJA	4MXU
4RXK	4JGX	4R82	4KP9	4XUV	3WG3	4Y13	4RY8	4BMU	4YRU
3W2J	4D5Q	4WSH	4HMW	4FQW	4NUA	4USA	3WXO	4QNC	3WVA
4C6P	3ZOL	3ZPN	4FZO	4OMG	4TN4	4NPU	4N1Y	4PXD	4KQE
4ME9	4O1W	4P3M	4W7C	4UPD	3ZOA	4UNL	1ORF	4Q0J	4NL5
4O4W	4M51	3WXZ	4PHC	4FRH	3WAN	3WJC	4TPT	4P82	4Q9B
4N6Q	4C72	4DMF	4UVJ	4CKI	4BMY	4MIS	4CTD	4WBD	3VWN
2J9K	3W1W	4JC2	4QFH	4UCH	3WU0	4JZX	4CUR	4IQB	4Q7O
3WJK	4US5	4D3T	4NQU	4PV4	4P4U	4BWI	2AZB	4IGM	4RDW
3WYW	4IPP	4MYY	4UP7	4RDL	3WCN	4MK6	1V3Z	3ZG1	4OMQ
4QMK	3W77	4X8C	4MJK	4P53	3ZII	3WJG	4L03	4MB6	4KOA
4NVT	3WRW	4MPL	4KPD	4OTM	4OYH	4PSH	2WLV	4WH5	4N7J
4WZI	4C8P	4ICQ	2YOR	1HEG	4GQM	4FQS	4JO0	4LIT	4W5K
4OOE	4WCW	4W4R	4WZ8	3ZCP	4BSW	4S1W	4U9A	4PCL	4L04
4NVQ	4LON	4M57	4CIJ	4LW2	4CUO	4BW9	4KLC	4URP	4LS7
4C08	4OTN	4KPW	4FYM	4WER	4RJV	4D1P	4G09	4OM7	4MID
3WQM	3X16	1C1X	4Q4K	4PGG	5AK2	4CVT	4LMV	4RKK	4P3V
4KPO	4NHF	4RPI	4R9L	4O0F	4Q62	4D5S	4C2E	4N8P	4CN0
4PYQ	4KZN	4PW0	4IJY	4OH3	4P80	4GCV	3DCK	4UUY	4XVZ
4LAN	4XLV	4MP8	4OR8	4XYZ	3WY7	4BTX	4L6Q	4J8Z	2YOB
4RFA	4OO0	4XM5	4NHW	4XJQ	1K2B	4PNI	4PCV	4XEA	4FRO
4GRC	4U3E	4NSR	4D5I	4NAD	4O6I	4LE6	4K26	4OJZ	4UNV
4L4U	1A94	4QQ7	4JU5	4BJ4	4OA8	4RNA	4OOJ	4GHO	4R5F
4C91	4KFA	4KV2	4MAQ	4MJF	4KPJ	4QHR	4KCX	2GSR	4KER
4FRQ	4C0N	4RQT	4QLY	4M4X	4TKR	4JEF	4N4W	4OKE	4PXE
3TM3	3WOK	4TWE	4RG7	4LQX	4NH4	4TQ2	4XCV	4D3S	3WQN
4MQD	4TTB	4LZA	4TVO	4Q05	4O1U	4WAI	4R2K	4R0B	4HA7
4JE6	4KIB	4LDR	4W9R	4O3V	4CUD	4O7H	4NOC	4Q2N	3W6S
4BEX	4RI1	4RHP	4R8Z	4MZA	4NPX	4L7E	4DMK	4L69	4PHJ
4XIN	4WII	4U5H	3WVO	4KT8	4CO9	4TOP	4OQZ	4C5C	4OPF

4X7Y	3ZBL	4R14	4KP2	3X1B	4RT0	3VWM	3WO1	4RP9	4OO7
4LUL	4YIC	3ZIE	4GT2	4QIN	4IZU	4HHD	4KUK	4WIS	3WJF
4O8T	4X1E	4N05	4MUO	4PDT	3WJD	4LE0	4PON	4ILF	4N9J
3WOJ	4R7Q	4CVY	4M0Q	4RCV	4NQI	4GSN	4L96	4M23	4RNI
4WSE	3W8Q	4KY4	4X4W	4CL7	3ZOK	4OOV	4YF1	4PW1	4NTD
4G2R	1C1X	4NJS	4M8M	4JT4	4IZH	1GQP	4FFJ	4D1E	4Q3O
4BOU	4X26	3WDM	4PPU	4UBT	4QC2	4MWS	4CPK	4K1U	4OJX
4CF5	4BNB	4L9J	4UUU	4KP9	4O9K	4OVE	4P5X	4S24	4BMK
4OAG	4IA8	4P6D	4YFV	4MPC	4Z1I	4U9N	4CII	4CF4	4L74
4M8Y	4BRK	4X54	4HK7	4XCZ	4NMC	4N0R	4PZJ	4W6D	4XAR
4BY5	4IX3	2YPY	3WY0	4CVH	4MTS	4ND6	4RJ0	4OJT	4RGP
4I6R	3ZHC	4RD7	4LOA	4XGO	3VWQ	4MMO	4RI7	4BVA	4LW7
4JH2	4OOA	4OB6	4OEJ	4COG	4O0E	4I71	4NXI	4KEP	3WWV
3ZKB	4RSW	4OH2	4IJ7	4KBX	4BJQ	4C7V	4M73	4KGB	4X51
4QBO	4W99	4MZ0	4JBR	4MY2	3WTD	4NKE	4O6M	4BEQ	4C18
4NMI	4N81	4PXO	4XIY	3WWO	4MZW	4CHL	3WHB	4COO	4RIG
3WX4	4NE7	4P5W	4IZS	4O53	4PMU	4PBB	4XDZ	4R1H	4Z24
4MCS	4HS2	4QND	4R1I	4MYB	4NVS	4MSO	4HSL	4NFR	4NDA
4R4Y	3J7T	4Q16	4XLL	4QBA	4WWB	3WFW	4TMC	4HPW	4WUO
4MXT	4RK1	4IGN	4TS5	2YQ1	4NQT	4Q0M	4XLO	3WK6	4OL9
4KV9	4CO8	4RNH	4QA0	4HAR	3WRV	4NK2	4R9K	4PFZ	3ZML
3WGT	4CI9	4BIA	3W60	4Q97	4RGD	4D6Q	4NWS	4HKE	4BZP
4RYB	4N1V	4IZV	4RZE	4PR3	4CND	4KUH	4OAQ	4LBU	3WWM
4JBL	4CNQ	4B8V	4MDS	1K1T	4M7X	4LSB	4R2J	4JEQ	3VWP
4QHG	4MNU	4M6X	4PUP	3WRB	2AIG	3ZDR	4LH8	4QYS	3WYD
4R8E	4OHQ	4Q5R	3ZEI	4NEW	4RAA	3W5Z	4LLE	4M9R	4MCB
4LDS	4KTW	4WUB	4MXM	4N2H	4CP3	4O0C	4O47	4Q76	4HMS
4QFJ	4HWT	4RRW	4MCN	4N0W	4O8B	4O54	5AIR	4LOW	4PB4
4CW4	3ZCS	4JPH	3WKX	4RGK	4BRD	4WBT	4PWY	4R60	4OW5
4RZ7	4MK3	3WFZ	4MJZ	4Q51	4NAS	4XR9	4QXO	4TRT	4N0V
4U6D	4PUM	4NES	4R7F	4LTB	4RZF	3WG4	4YVD	3RVB	3W8S
4CHI	4IRG	4NAZ	3WJP	3W20	4HEH	4S0K	4LIS	3WML	4MTC
3WO8	4R9Z	4RFQ	4NZY	4C6S	4MYM	4RZ3	3W8G	4KTI	4LN9
4YEA	4MZV	4TOI	3ZHK	4MEA	4RMJ	4K87	4PWW	3W61	4Q5O
4M0K	3IFD	4OI3	4UBW	4LLS	4XHC	4HHP	4LUI	4Q7I	4N4C
4L9E	4M72	4TO8							

Table 11. List of PDB IDs of all new monomers (Test-set 2)

3W42	3WDQ	3W5F	4MSD	3WO6	4YZ8	4HAL	4J7W	4V16	4L6P
4Q9H	4FXY	4EQC	3W0S	4XFK	4TMD	4O6E	4LGJ	4H34	4NNR
4PJR	4N7A	4K91	4QXL	4L4H	3WBY	4CL2	4CFS	4PP4	4C2Y
4N1X	4NMY	3MDJ	4HQZ	4RLV	4IK5	2YP6	4K11	4UYE	4MLA
4POI	4PIP	4ILY	4QBE	4TS4	4N01	4KUL	3I3J	4L4G	4TPN
4IFJ	4CIT	4R1J	4BYM	4NV5	4B5Z	4MLV	4BM8	4OY4	4UR4
4N0N	3VXK	4NU2	4ROW	4O25	4MNP	4MX1	4PX9	3WT9	4P29
3WPF	4KF4	4O2X	4OO3	3W8K	4PQO	4OY8	4LT9	4LND	4RY4
4O8G	4PQJ	4MJE	4C75	4NGU	4K0N	4L3L	4R3C	3WZ1	4M7V
4CIZ	3ZBU	4RXT	4OEA	4M68	4QLG	4N0Q	4OH0	4LA3	4QTB
4C7D	4WO5	4QHW	4IUG	4QY4	4CZN	4OAN	3WEI	4N14	3WH1
4Q4N	4U3V	4TLP	4KWE	4JCN	4MX8	4IC4	4PIO	4LU4	4MI5
4KYC	4K33	4PZV	4K5Q	4M1V	4K19	3W8M	4PMA	4LR2	4LIM
4G4X	4LZL	3WOV	4IRL	3WUQ	4RH0	4HRA	4M4Z	4LAA	4TY0
4R6D	4Q0K	4QPM	4UP5	4JTR	4ROV	4RCI	4PQ0	4BM1	3WLT
4NYZ	4BTV	4BMJ	4OO4	4OMK	4P56	4MQU	4YOK	3W9V	4CDJ
4U9Q	4YFS	4NSS	4QDJ	4WE2	4LTZ	2YPM	4C4M	3WSP	4LDJ
4RBM	4MSX	4I2Y	4QLH	4CQI	4IZE	4MNR	4I04	4QJL	4BX0
4KA8	4BR5	4KRI	4MBR	4O8O	4QK3	4O00	4NSP	4LQZ	3WWC
4NLY	4NS5	4Q63	4URN	4MKH	4MRU	4L5V	4D6G	4LPC	4NON
4UF0	4PIV	4H9W	4LHV	4JS1	4PQ6	4M90	4I5X	4O3X	4P09
4QEU	4C0K	1A5I	4G3N	3W2Y	4LK4	4MAD	4R98	4PVA	4TN8
4PO4	4RWH	4NFE	4ME8	4WVL	4UMG	4OKR	4O7I	4UIT	4BQ2
4BXQ	4RTI	4P2F	4CJ9	3WA8	4L15	2YNU	3WR2	4M4P	4GT8
4K82	4OZX	4I4C	4N9P	3WF7	4POY	4LJ9	4LZ6	4LW3	4JA7
4C2X	4CFI	4MMG	3WLU	4HP5	4KLZ	4K47	4R9F	4TR0	4B5E
4O3F	3WHP	4BG4	4P8O	4G32	4WBY	4B5N	4XVH	4KKY	4M82
4LE8	4MIW	4OTF	4RUN	4C8D	4P15	4RI5	4Q5M	4BZY	4UMT
4CH7	4XE4	4U0P	4N4B	4R39	4L7V	4MFJ	4PPJ	4N6T	4O33
3WQ8	4P0C	4MFH	3W8L	4B4X	4QYN	4QTZ	4BSX	3VVG	4CRR
4RSH	3ZK7	4G97	4Y9T	4MD6	4MTH	4ME2	4CRS	4PU5	4MLL
4BGF	4WSO	4NBO	3WT1	4W59	4KR1	4Q2C	4L3W	4PHF	4WK7
4Q2W	4LW5	4JB1	4KW3	4LTT	4J5L	4X55	4Q88	3WD1	4BG5
4V38	4RX8	3W0Q	4CK4	4NFB	3WNO	4UZL	4QUK	4LUH	4L6G
4CHH	4YAH	4KFW	4MEW	4YGJ	4QS9	4QA1	4LT6	4C29	3WCQ
4QGW	4XP8	4R6W	4BQN	4QWO	4MXO	4QQS	4LZC	4L1D	3WWL
1FH0	4QQT	4PAB	4MYU	4WGI	4BJK	4RNL	4IEB	4TMT	4KNN
4PBH	4XJ5	4BPC	4MQ3	4M4U	4L6R	4IL8	4HRO	4RY6	4D1S
4P7Q	4JRU	4HQS	4CJM	4PSU	4KMA	4NOJ	4KIA	4MM0	4BUH
3ZIX	4FDT	4COU	4X92	4L78	4M2V	4MJB	4OHI	4P8N	4KP1
3WEW	4P9K	4WQK	4UMW	4QAL	4OVR	4CP6	4ORN	4BF7	4Q07
4MO1	4P55	4NV0	4LEE	4O1S	4Z02	4LZD	4UU4	4NFM	4O5T
2Y72	4JHO	4O0S	4C2O	4R2B	4GQK	4D0P	3WDW	4N6X	4JR6
4P8V	4CEK	4RYA	4QAW	4V2U	4NI6	4CZW	4V2A	4I84	4WGH
4IQ2	4JOX	4JWJ	4KNK	3WP4	4MXX	4C23	4KRG	4KEI	4L0F
4N82	4KE6	4O4C	4PTB	4PF4	4OZ5	4NZQ	4NOV	4YOD	4YPJ
4LII	4MR0	4QLE	4UUC	4P0G	4J3B	3W81	4GO8	4JL5	4LOT
4R61	4ODG	4Q4G	4ZBG	4LSA	4Q91	3WQ6	4NCK	4K84	2O40
4Q53	4RK9	4M01	4Q9W	4CSS	4LZI	4LEG	4I08	4L3N	4MX6
3ZJ0	4N57	4QBI	4RWZ	4O6G	4BQS	4I2D	4MVF	4WUI	4D6E
4QAU	4HZG	4ULW	4RV5	4OUJ	4MGJ	4RKF	4LGG	4BKO	4P5S
4WA9	4Q8G	4NUX	4KEK	4UBH	4OQR	4X96	4O29	4MZC	4O9L
4O08	3BFN	4LEW	4BHR	4NUY	3ZIV	4PA8	3WJU	4BBP	4W8B

4UM2	4N08	4NJC	3WNK	4CB7	4OZA	4Z7X	4NUI	4PPT	4JCG
4LGN	4IJG	2YNX	4UWR	4QQ5	4M8I	4KM7	4KMY	4R12	4YBG
4C1D	4L4E	4KYV	4Z48	4J3V	4O5P	4CSV	4QNY	4OFL	4P0O
4KFV	4JMQ	4QU3	4MJA	4MKM	3WWG	4BN1	4WOK	3P9U	4C4D
4PM8	4BIN	4XHK	4C9P	4RV9	4PGR	3ZFY	4QCJ	4WEI	3WBJ
4RLK	4KRU	4POW	4QY7	4CUA	3WDV	3ZGJ	4BVW	3ZLE	3WHK
4PF6	4RV7	4MHQ	4WZR	4M5E	4CSI	4XEQ	4KZS	4WBS	4CC0
3WKZ	4K3B	4LRC	4YJW	4CBT	4TUU	4Q2T	4D6H	4CTJ	3WJ9
4WOH	4IBY	4M9Q	4W7O	4LJ1	4HIC	4CQE	4MQL	4OLK	4D6S
4JP6	4R9V	4PVE	4M00	4LEF	4TYX	4GN2	4IFG	4QBU	4RDB
4Q03	4YUD	3WW9	4WJU	4O2T	4LQU	4M8U	4PUI	4R80	4OD6
4PJL	4K7J	4Y0G	3WP3	4JU3	4O8Q	4TN0	4PHG	4PJK	4QPW
4X8E	4CVK	4LZR	4CJB	4NRV	4OY7	4YO7	3WVS	4C4A	4B7B
4J0M	4LQ4	4MM7	4NKL	4JZ5	4BTK	3ZK9	4M7G	4CIK	4O6U
4CGE	4MLN	4MYV	4NNG	4M5B	4K6E	4BEC	4QYG	3ZQ3	4Q67
4XPW	4UWH	4MU6	4PJ3	4KYQ	4LU3	4K3A	4M7R	4QBW	4PGI
4BRS	4PSF	4MDA	4PF3	4O3Z	4KEX	4MFI	4L4I	3ZOO	3ZFZ
4NQG	4H0J	4IF5	4GO9	4NAC	4LG9	4V2B	4CN9	4BG4	4MRY
4HLJ	4WFX	3WBD	4TWC	4XXL	4OAI	4BVN	4LTW	3WAO	4M6J
3WA7	4KAG	4PDH	4IFS	4H7V	3WL1	4OGW	4RG3	4NIV	4LEC
4Q4B	4PLM	4N5Q	4JJJ	4LYQ	4QAP	3ZM7	4LYP	4BMD	4PD3
4NXW	4CU7	4IOB	3ZFK	4NJ6	3X0D	4P40	5AHO	4P6A	4LLA
4QBH	4RU0	3WI7	4C7M	4AZN	4N1I	4PII	4C41	4N17	4UOS
4YWH	4PEW	4N3T	4S19	4LJN	4NWF	4V2E	4YU8	4TVW	4MAI
4MDT	4M1R	4LDC	4R58	4NNB	4CI4	4TVE	4OQ2	4PMH	3WHN
4CZE	4C5F	4LD6	4N4G	4MRQ	4O0Y	3WCX	4TLG	4NUZ	3ZHL
4N6C	4U2F	4N3E	4GZK	4N6K	4UWD	4Q1U	4KOB	4OBY	4MWI
4HPH	4CIL	4JGW	4UR9	4JGU	4BQ4	4G78	4N9W	4J2R	4NOF
4MIJ	4BJX	4C1I	4BY0	4BME	4NS4	4HKH	4N73	4OPI	4CDN
4XCT	4IUH	4QUC	4K55	4OO9	4C2R	4UA8	4TLW	4NET	4RK2
4NMU	4BA7	3WDH	4U63	4LW9	4P68	4H0F	4J1L	4V1S	4TWL
4R43	4O3W	4N4U	4CHE	4JZ4	4MQ1	4M7T	4CVI	4M16	4LX9
4BXV	3VVH	4BFA	3W0O	4W88	4WFI	4PNY	4QRL	4KYS	4LGX
4BVJ	4JOU	4XPX	4IM4	4PYG	3WJV	4YYL	4CB8	4OW0	4YGT
3WVN	4RUU	4X0J	4RVQ	4L8L	4Y2C	3ZPJ	3ZJ7	4KPB	4YIJ
4X3J	4TR5	4PZS	4BVK	4XPZ	4USR	4R0E	3ZK8	4JKQ	4LHL
4NYH	4WKQ	4QBF	4GNU	3W57	4XCU	4J18	4N7E	3W6R	4HKG
4WPZ	4QVR	4NJ5	4N77	4QRK	4ORL	4NCN	4L1P	4MXY	4U28
4BPF	4Q4T	4OR7	4OQ1	4PWN	4KC8	4RO9	4REK	4QPC	4UBS
4TSQ	4OW2	4OF0	4HCW	4PTX	3WPZ	4MMI	4MPZ	4LE3	4J8Y
4JGG	4PYR	4NPK	2YN8	4PVH	4CB5	4O2I	4K13	4C09	4W7G
4H5Y	4Q6O	4MUP	4KG3	4PM0	4OLL	4LNV	4XJ6	4IP1	4WF2
4OHH	4NA6	4NLS	4LPQ	4CCW	4W7N	3W2X	4R3F	4PX7	4KYP
3WLD	4MKB	4KSB	3WNY	4B41	3WN6	4KMP	4V33	4IYT	4UAV
4U86	3WKG	4IBZ	4NRP	4K6X	4ULX	4CIU	4OX3	4NBS	4MNK
4LNY	4C9O	4WF0	4BIH	4V24	4QSG	4LHK	4W6T	4L6X	4NF0
4BPR	4K1S	4C1S	4NWK	4U1D	4Q3G	4ONZ	4N6H	4J1Q	4I93
4KDC	4QNX	4BIG	4KIE	4C6Y	4WA0	4P0Z	4IBM	4NPW	4LXD
3ZHI	4LX8	4NLU	4M9O	4Q46	4D2O	4NWG	4RS2	4HH8	4LRJ
4RDP	4L5G	4IBT	4LZ3	4HMO	4RXU	4LHF	4MT5	4QDZ	4H1B
4RKB	4PNL	4R6F	4UQX	3WR0	4WXE	4GWF	4MMB	4HNQ	4L7T
4P0L	4MAG	3WGK	3WO9	4LJS	4TLR	4HLL	4QXD	4JXH	4P0K
4OGH	4X00	4NCH	4NMZ	3WCK	1CVR	4KG6	4WXQ	4S1E	4OAC
4P1T	4WTX	3WBG	4UZJ	4M5X	4LV7	4M4V	4K6G	4O6K	4COS
3WI3	4BX8	4WOD	4R9N	4OHN	4OG6	4Q11	4QAJ	4Q98	4PQ1
4JLV	4OGN	4C43	4I9L	4OHD	4QAH	4PQQ	4MYT	4WED	4INN
4O64	4OIY	4C84	4OVQ	4QB4	4L4P	4RN7	4OPB	4B46	3WGM
4BY2	3WMY	4NLB	4TX2	4LS3	4QPS	4L54	4OBC	4R1K	4LGC
4BMB	4US3	4M2W	4ITC	4NLP	4WJS	4WXS	4M9K	4N8I	4CNM
3WBE	4BZS	4L3F	4QEF	4JAC	4TXD	4RGJ	4OT7	4RGI	4OAE

4N1G	4OJ3	4Q7F	4FRX	4N19	4IL1	4W81	4PYP	4QKS	4O6X
4LQ8	4KCA	4CRH	4P2K	4LG3	3WIX	4WSL	4NQR	4JSD	4XDQ
4XX6	4KCB	4OCV	4M56	4LA9	4LWE	4JXD	4PFW	4LA2	4IXA
4NPD	4L5W	4IBQ	4WCK	4PTG	4NZ6	4GFX	4NQN	4C3Q	4OA2
4R1V	4RWE	4IYA	4HK8	4KDW	4C2P	4O9D	4INK	4WWP	4O89
4WUN	4NL4	4R8N	4M4N	3W9F	4CMP	4CJ6	4L60	4JCM	4IIR
4C7G	4IPD	4XPL	4CCZ	4WH9	4U0L	4CMT	4LTY	4V0K	4OCJ
4RYO	4QL1	3ZLB	4OLT	4MU9	4KBK	4YHC	4PGN	4OTK	4PRJ
4JG4	3W9R	4N3S	4R1U	4WEV	4KVL	4BKR	4DA2	4IRF	4XDO
4XAP	4GOH	4QDT	4GRJ	4LSY	4NMW	4O5J	4D6I	4S1P	3WMR
4P5U	4NAM	4D2R	4ID4	4NH0	3X0W	4RX3	4PFS	4UDR	4M1K
4G3A	4O94	4NT1	4O4U	4LDG	4P0T	3WEB	4U7L	4BHL	4KGT
4QFU	4P3L	4WD1	4QFL	4OZS	4CD8	4PMB	4HA2	4PQH	4TMX
4UV6	4KQP	4JWF	4JL2	4OHX	4NS0	4MBS	4OVK	4QT9	4CAG
4NXJ	4MGQ	4QMH	4MB1	4Q6T	3WDO	4BZX	4D77	3X2T	4OZW
4PSR	4YEO	4O2L	4XRE	4CG1	4P10	4RV1	4NW6	4CGS	4IP6
4U3T	4U3H	4PQK	4KG0	3ZNY	4LVQ	4LY1	4U7K	4CNL	3ZJA
4OJH	3X17	4CSO	4RS3	4U4E	4RSX	4C16	4RJ8	3WYA	4JXE
4W4M	3W1D	4X90	4QU4	4INO	4Q3X	4G4P	4R2F	4YHS	4BVC
3WE9	4P0D	4L3U	4X9P	4JR7	4L2H	4M8V	4URL	4LXW	4R7R
4HSC	4PMK	4K89	4JJO	4PEU	4PQI	4M3O	4IK1	4IZX	4OLI
4QK2	3WLI	4RPV	4LWM	4KRN	4MDY	4QTC	4UWP	4X56	4RNZ
3WCZ	4N0P	4X7K	4CT2	4W7D	4KZG	4NV6	4PE2	5AF5	4RZP
4KBO	3VXG	4R0Y	4MES	4PQ9	4MN6	4NPS	4GL1	4U2D	4JDG
4N8Y	4QI9	4BW8	3WIV	4PCF	4PPL	4RUW	4B8E	4PHR	3WH9
4P47	4PMO	4U0T	4Q28	4O4P	5AJ8	4CDO	4K7K	4KNH	4BFM
4LL9	4OPL	4QDH	4TT6	4RHO	4R1S	4YYF	4HOZ	4D72	4Q9P
4OKO	4XXR	3ZFX	4M5I	4TRK	4TTR	4CYF	4WE6	4LJ6	3W0P
3WV4	4OF6	3ZNT	4W9S	4M6H	4PVJ	4L9O	3WAR	4H48	4C2N
4MNC	4IYC	4TJV	4PPK	4FF5	4PNE	4R6J	4M3A	4M83	3WEC
4JMN	4MRH	4N6W	4O8M	2YN1	4N67	4WSQ	4D1T	3W53	4PBQ
3W94	4P4G	4HDH	4OCF	4CVB	2JAC	4R3V	3WPY	4PXG	4N8H
4PFI	4JCL	4CSB	3WZU	4LBA	4D7R	4RWR	4BOK	4QTD	4J9B
4KMW	4LH6	4LRD	4PQX	4C7P	4J9J	4OXI	4BS6	3WIB	4RQR
4W7M	4C0P	4U5A	4LYR	4AYS	4TYP	4O95	4P32	4IDI	4KF3
4HCS	3WFL	4PQZ	4OVJ	4QS8	4LBL	4Q3Y	4KI9	4Q65	4IK2
3W7X	4GBO	4HIN	4M0N	4Q6Z	4XIJ	4PY1	4R7G	4Y2A	4D01
4JV4	4Q6V	4N71	4HAK	4GYD	4QPN	4OI9	4J0O	4MDP	4OL7
4PAA	4N03	4J51	3ZCF	3WWA	4OFF	4LES	4CU9	4WRP	3W9U
4BVV	4J3W	3WRZ	4OEN	3WGJ	4L1I	4KUI	4OTW	4KG4	4TPW
3WPQ	4ME3	4PT1	4IPG	4NPH	4CDB	4Q24	4LTU	4LAX	4IQH
4OF9	4OOD	3WNP	4BMC	4W65	4OW1	4LRF	3WUZ	4P48	4D1U
4C54	4QTP	4C0T	3ZEW	4OXW	4N8K	4CS5	4OVX	4U0I	4MPS
4V2Y	4MLZ	4PWZ	4N6L	4MJC	4L4O	4KEJ	4LRE	4P6G	4MYD
3W1C	4ONU	4QBV	4Q3H	4LM9	4LD1	4MCK	4YAY	4QJK	4PQD
4NLC	4CD5	4NU0	4KB4	4KYR	4WHI	4L6S	4X9T	4ODD	4C6H
4CQH	4O0W	4MKF	4LIK	4CNR	4PFX	4XP7	4C97	4OOK	4BXM
4OGE	4OZQ	4HSQ	4LG8	4BMH	4KN3	4I35	4YGS	4OZU	4MFZ
4CDL	4K1P	4TXA	4XRV	4MT7	3WDB	4LIZ	4FX7	4YLE	4WCX
4GVJ	4LSO	4IED	3WAJ	4XS5	4NNO	3WYH	4RSQ	4KP0	4PVK
4OMB	3W7W	4Y7U	4LJM	4MXN	4NN2	4IKG	4PYS	4CXK	4HH5
4NLR	4PYT	4OXP	4KKF	4FMP	4MO0	4C1U	4PMQ	4QHE	4NJ8
4KL0	4MO9	3ZOZ	3WPU	4KO5	4C3Z	4QDX	4W5B	4PZ7	4J4J
4JRN	4B97	4JHV	4I05	4ON1	3VZ6	4J5M	4BCT	4RJZ	4CNX
4PL2	3WT0	3WG6	4LDF	4MZD	4NF7	4LPS	4X2R	4C49	4U4J
4OOS	4MDW	4Q3Z	4LPI	4MEZ	4Q5W	4ONO	4L1L	4OLJ	4NXT
4M85	4IGI	4Q5I	4BQH	4QDR	4NX9	4Z2X	4WV5	3WVF	4LMH
4RNX	4BZ7	4INZ	4ONY	4PZ0	4MTN	4MXG	4Q1Q	4LUU	4GXZ
4QN1	4L1Y	4QD4	4TKO	4Q7G	4H05	4XUY	4GQP	4J6P	4MHP
4L77	5AGD	4M02	4KB2	4QKT	4RZ2	4D7Q	4Q6B	4CGO	4BLU

4RXM	4PEF	4NCI	4UDQ	4Q9G	4OGM	4LYA	4PC9	4ORK	4OPG
4X2Y	4OOY	4MZI	4OGP	3ZFM	4RKL	4HOW	4W7J	4C7A	4YME
4P8B	4UAT	4B4L	4UWW	4LAE	4L5U	4RY5	4KM6	4LCB	4RPC
4R3A	4Z0N	4LRU	4N49	4V32	4K68	2YN7	4D6V	4R6K	4WZZ
4PF8	4NOL	4OTP	4RCG	4TYZ	4OBM	4S0V	4GQG	4NOB	4OZD
3WH2	4O8H	4ME5	4BQ1	4HYJ	4QP5	4NW4	4OBE	4CYS	4UOR
4WDC	4BOJ	4D0Y	4UMQ	4JXF	4QKR	4QB6	4JO5	4KLX	4XDI
4OA5	4COT	4CMN	4HQ6	3WQT	4KL5	4LJY	4R5Q	4NTS	4R84
4B5C	4CZL	3W0A	4Q3I	4LJ7	4M2B	4IJF	4TRD	4RY0	4CQ8
4OVS	4YYX	4MMP	4XE0	3WL0	4UY6	4TKB	3WIW	4P7B	4RCW
4D0Q	4PKX	4OFQ	4S1J	4MMJ	4OPT	4LE7	3WHI	4V2D	4L2L
4QM9	4N6M	4Q6X	4CHJ	4CT5	4N9T	4Q5T	4QP0	4XA7	4LEU
4PN7	3WRT	4JA2	4REZ	4N0L	4KSE	4NLT	4QHQ	3WEK	4N75
4LUR	4BPY	4OON	4CYR	4ONR	4JZQ	4LWL	4PSC	4RKE	4LWK
4O7K	4BUP	4S06	4YMX	4NA5	4CO7	4BLK	4Q6A	4XPY	4NH9
4Q6W	4IZL	4I2U	4RW0	4K2D	3WMS	4JZR	4Q2S	4TSM	4U5X
3ZGH	4BTF	4RJ3	4NOA	4TXP	4C6R	4V3C	4U0N	4MAZ	4D1A
4P9J	4PQ8	4XTL	4JS7	1SCN	4GWR	4Q8I	3ZL8	3ZMR	4XY3
3ZM3	4TNS	4U98	4UWJ	4M1B	4QKN	4TZG	4OYV	4WQN	4MZ2
4QDI	4CSH	4OQS	4D04	4U8Z	4Q8K	4D6D	4QX5	4BPZ	4UML
4FSO	4P3K	4WMH	4R4G	4MQM	3K8L	4TVF	4J1P	4TN2	4PM4
4TPK	4H5R	4LGM	4ORW	4Y93	4CHD	4MST	4TZ1	4PC4	4OER
4UF0	4Q7Q	4PS6	4B2N	4Q3J	4U9U	4BJI	4LWR	4LKP	4H8H
3WL5	4NLQ	4M8K	3WPN	4R0V	3WBI	4XUZ	4MNO	4Q2A	4BJ0
4QIM	4LQT	4LJX	4C5P	3WPV	4XXN	4BZ4	4PO3	4PAG	4LUA
4PPF	4NZ7	4J0L	4PP0	4M0H	4JPM	3WAL	4OZB	4MYR	4IMH
4D40	4JFN	4PWP	4OGZ	4Y95	4MC3	4WCM	4N1L	4UXV	4L21
4R6Y	4UTO	4PEV	4QA8	3W0R	4RVA	4NG0	4N13	4H9J	4NDC
4YMQ	4UXQ	4QME	4PM5	4O2R	4RGL	4X2J	4H7U	4QNW	4UYN
4BSM	4BEB	4M7O	4QQC	4WD6	4P1E	4PQB	4TYJ	4XUF	4R03
4QUN	4OF5	4RG8	4O9F	4QGL	4R9P	4M46	4UYD	4LUN	4RTH
4M7U	4WHZ	4ICU	4B9P	4H60	4L4F	4KDR	4PQW	4UY1	4OR1
4JRB	4ROB	4CIH	4NQ0	4R6G	4URO	4RM4	4NLW	4IXL	4YS6
4IV0	4XHM	4UIX	4UBG	4OM9	4P27	4OCC	4CD3	4N5C	4OSN
3WDF	4BOE	4TR3	3VW8	4M29	4MXP	4O7M	4JMP	4QX0	4NDX
4MYG	4D1V	4NPM	4WCV	4V20	4LAB	4QI0	4UYB	4OEB	4HKL
4TUM	4NCE	4YDW	4UON	4OPU	4NS2	4M2L	4QKF	4NZK	3VWO
4OCE	4PK1	4MYQ	4J7Z	4NRU	4U9B	4PT6	4WHA	4JFS	3ZBT
4RY1	4U2E	4URG	4KJT	4WUT	4Q59	4P5P	4R3H	4FSP	4NQ8
4C0M	4R0M	4N8L	4QOS	4Q4L	4K1X	4KW4	4N0K	4X2Z	4CP1
4P5Q	4LSC	4PBD	4Z1F	4BE7	4S1N	4RKS	4WT9	4QRJ	4LGY
4NPF	4QGO	4QDG	4QC6	4BOB	4PE6	4OHL	4RAW	4NFC	4LRM
4IM9	4CEM	4L4D	4ORI	4OTH	3WOB	4NUH	3WIJ	4M1N	4RA4
4JD0	4BN2	4RFZ	4R5C	4OX2	4H1V	4MEC	4R7S	3WPT	4O5S
4M66	4TWP	3WGE	4D4B	4M0M	4XVO	4B9C	4PYH	4TZA	4UY3
4BZF	4OXX	5AF0	4UBQ	4WXT	4OBI	4PSJ	4C9Y	4CUF	4QB0
4R1B	4NFD	4O4X	4RY7	4R6H	4LQM	4OU0	4TMV	4RSM	4OMO
4N7X	4V14	4MY4	4WEK	4QEY	4PDY	4D4Q	4U9V	4JD9	4O06
4P84	4TQL	4BZA	4N30	4IJT	4OGB	4OA3	4CRQ	4PLA	4LPL
4IMB	3WL6	4L5Q	4TYQ	4MMH	4PC8	4B6L	4RVZ	4W9T	3WBW
4RXL	4LX4	3WFN	4QAN	4QQ6	4OPW	4L7G	4JLF	4CNK	4P9A
4N5X	4LM8	4N0D	4NY6	4ONQ	4PXA	4N6D	4Q7Z	3WAQ	4OJG
4K3C	4NX1	4NXC	4U3D	4PWT	4NOH	4QQJ	4KVX	4YCS	4NZZ
4MT6	4N6V	4CFP	4PX6	4M97	4IO0	4I5P	4QQG	4PQC	4PKW
4OHE	4W7K	4WUY	4QB8	4D6J	4NNK	4W5W	4NLX	4W8F	4R3E
4BEW	4OEE	4LWJ	4PYI	4BVL	4UW9	4NXY	4OJB	4XQ1	4PQG
4WCJ	4JLP	4WEE	4NG7	4UVK	4D1W	4LOS	4ILE	4PGK	4PXW
4D71	4NDB	4OE9	4W79	4KSM	4PRW	4K9F	4KQ8	4MCO	4U6R
4LZ2	4HU8	4KRS	4N5A	4QLR	4XAH	4KS7	4MAA	3WRH	4PFY
4TVT	4GAZ	1BDA	4QA4	4UT1	4ORF	4XKK	4MFK	4IWB	4PAO

4CNW	3W95	4QEK	4WAT	4NA4	4OY9	4PV6	4JPX	4RXX	4PUT
4LCL	4B62	4QT4	4NHX	4PFO	4KZV	4GGV	4OVT	4WXJ	4C1R
4P8S	4CBH	4LZH	4OG4	4WWH	4N3G	4YV7	4TNM	4QTA	4IPU
4MB5	4JVL	4NL0	4WT7	4ICW	4BGC	4RX2	4K46	4JJC	4IGY
4S2V	4CK8	4OR4	4O1N	4NZJ	4M9T	4NDD	4PYZ	4JNW	4PMX
4RXV	4H1O	4QGY	4P66	4IPC	4XFE	4QHF	3WBA	4XXT	4CBY
4OQV	4MUX	3WH7	4NLV	4JP8	4MEE	4W7L	4O0U	4QLF	4KVK
4RDN	4PED	3WIU	4OFJ	4XH7	4OYL	4IDY	4ROA	4Q2R	4PL7
4QAK	3WUX	3WCA	4Q3W	4NHB	4RF6	4J25	4OZV	4O6Q	4NNH
4IHK	4XXP	4C1W	4QK1	4UU6	5AIL	4KX7	4WF8	4C8X	4PH8
4O9X	4R1A	4LKS	4QN8	4XUX	4QYY	4Q0N	4TZJ	4P0J	4U2C
4OOU	4D2S	4BPS	4HBA	4WJ2	4QFI	4KKI	4M9B	4M65	4LR8
4NBP	4TVV	4NLM	4LBW	4M88	4U2G	4L22	4Q38	4CBE	4URI
4XNV	4D5A	4LXJ	4IT3	4KII	4XV0	4NE4	4UAS	4QMI	4LC2
4REL	4RTF	4U70	4U7A	4KGR	4P17	4NLO	4OXJ	4X7R	4QBG
4IVV	4AUR	4UZ1	4KH3	4BK7	4K9J	4CNV	4KGS	4L9H	4NKT
4RI4	4JMU	4K77	4XVC	4U2B	4C76	4X8R	4NCS	4L6U	4OK0
4PAQ	4L2J	4NSX	4Q00	4N5U	4RL1	4UW3	4JWH	4JF5	4KO7
3X1O	4QE3	4RNF	4RN0	4NYQ	3WEJ	4MNL	4W8Y	4TZI	4MAN
4KAW	3WP5	4O2W	4WE7	4KAV	4PWQ	4Q08	4Q1Z	4OEC	4R21
4U9S	3W4R	4QPV	4OZC	4Q9T	4IMM	4MCU	4ITK	4U0M	4O8V
4Q8L	4FT6	4CXO	4RYE	3X2S	4OEV	4PMS	4WNW	4Q4F	4C00
4O8S	4FRT	4MIU	4JP0	4RNW	4PWS	4O7Q	4PWO	4C20	4OUQ
4P2I	4ID8	4LZ0	4PDN	4UTC	4WH8	4MO3	4WH6	4N74	4NEJ
4OKU	4CS6	4CZG	4P1Q	4NN3	4NTJ	3ZM1	4DAP	4XZG	4MI7
4B43	4XQ7	4PZP	4KX4	4V28	4U48	4IT7	4K0W	4LY7	4YGU
4Z9M	3WX5	4P6Q	4MHC	4M9P	4LLI	3WLQ	4JNI		
